# Shape or size matters? Towards standard reporting of tensile testing parameters for human soft tissues: systematic review and finite element analysis

**DOI:** 10.3389/fbioe.2024.1368383

**Published:** 2024-03-26

**Authors:** Alvin C. Lin, Felix Pirrung, Justyna A. Niestrawska, Benjamin Ondruschka, Gerald Pinter, Petr Henyš, Niels Hammer

**Affiliations:** ^1^ Division of Macroscopic and Clinical Anatomy, Gottfried Schatz Research Center, Medical University of Graz, Graz, Austria; ^2^ Institute of Anatomy and Cell Biology, Paracelsus Medical University, Salzburg, Austria; ^3^ Institute of Legal Medicine, University Medical Center Hamburg-Eppendorf, Hamburg, Germany; ^4^ Institute of Materials Science and Testing of Polymers, Montanuniversität Leoben, Leoben, Austria; ^5^ Institute of New Technologies and Applied Informatics, Faculty of Mechatronics, Informatics and Interdisciplinary Studies, Technical University of Liberec, Liberec, Czechia; ^6^ Department of Orthopedic and Trauma Surgery, University of Leipzig, Leipzig, Germany; ^7^ Fraunhofer Institute for Forming Tools, Division of Biomechatronics, Dresden, Germany

**Keywords:** aspect ratio, dog bone, dumbbell, human soft tissue, ISO material testing standard, non-tapered, rectangular, tapered

## Abstract

Material properties of soft-tissue samples are often derived through uniaxial tensile testing. For engineering materials, testing parameters (e.g., sample geometries and clamping conditions) are described by international standards; for biological tissues, such standards do not exist. To investigate what testing parameters have been reported for tensile testing of human soft-tissue samples, a systematic review of the literature was performed using PRISMA guidelines. Soft tissues are described as anisotropic and/or hyperelastic. Thus, we explored how the retrieved parameters compared against standards for engineering materials of similar characteristics. All research articles published in English, with an Abstract, and before 1 January 2023 were retrieved from databases of *PubMed, Web of Science, and BASE*. After screening of articles based on search terms and exclusion criteria, a total 1,096 articles were assessed for eligibility, from which 361 studies were retrieved and included in this review. We found that a non-tapered shape is most common (209 of 361), followed by a tapered sample shape (92 of 361). However, clamping conditions varied and were underreported (156 of 361). As a preliminary attempt to explore how the retrieved parameters might influence the stress distribution under tensile loading, a pilot study was performed using finite element analysis (FEA) and constitutive modeling for a clamped sample of little or no fiber dispersion. The preliminary FE simulation results might suggest the hypothesis that different sample geometries could have a profound influence on the stress-distribution under tensile loading. However, no conclusions can be drawn from these simulations, and future studies should involve exploring different sample geometries under different computational models and sample parameters (such as fiber dispersion and clamping effects). Taken together, reporting and choice of testing parameters remain as challenges, and as such, recommendations towards standard reporting of uniaxial tensile testing parameters for human soft tissues are proposed.

## 1 Introduction

The intrinsic material properties of soft tissues have been investigated and discussed in the fields of biomechanics and tissue engineering, in the development of biomaterials for medical use, and in elucidating mechanisms of tissue injury and disease pathologies (Fung, 1993; Payne et al., 2015; Ma et al., 2018; Garreta et al., 2021; Liu et al., 2022; Zhuang et al., 2022; Adu-Berchie et al., 2023; Biro et al., 2023; Chanda and Singh, 2023; Durcan et al., 2023; Karrech et al., 2023; Nesbitt et al., 2023). For engineering materials, standardized testing protocols are available to evaluate the material properties of a sample. For biological soft-tissue samples, however, such standards do not exist. As a result, highly diverse experimental setups have been reported, which can contribute to systematic bias (Innocenti et al., 2017; Zwirner et al., 2020b; Fischer et al., 2020; Wale et al., 2021; Chanda and Singh, 2023).

Soft tissues inherently come with a unique set of characteristics that influence how mechanical testing experiments can be performed (Fung, 1993; Wale et al., 2021; Chanda and Singh, 2023). In broad terms, soft tissues include all tissues except for bone and teeth, and are composed of mostly water, proteins, lipids, and glycosaminoglycans. The main structural protein component is collagen, and the orientation of collagen fibrils varies across different types of tissues. Along with interindividual variability, the same type of soft tissue (e.g., ligaments) can differ in their geometries based on their anatomical location (e.g., knee versus hand). Also, there are often restrictions in the amount of starting material and the number of human tissues samples available for research, which can further present challenges in achieving statistically significant and reproducible results.

In mechanical testing of soft tissues samples, sample geometries (e.g., sample shape and dimensions, especially in the middle gauge section) are known to influence the stress-strain distribution throughout a sample (Fung, 1993; Anssari-Benam et al., 2012b; Innocenti et al., 2017; Fischer et al., 2020; Wale et al., 2021). For tensile testing, evaluation of the stress-strain state in the gauge section is relevant, since the intrinsic material properties can be derived based on the assumption of a homogenous, uniaxial stress-strain state in this area, with the most dominant stress anticipated in the loading direction. In contrast, for an unknown and complex stress-strain state, it is not always feasible to apply the appropriate laws and theories to extrapolate the material properties of a sample (Horgan and Simmonds, 1994). For example, clamping of a sample can produce a complex stress state, especially towards the clamped ends, whereby normal and shear stresses stack up in multiple directions (Jimenez et al., 1989; Sun et al., 2005). This scenario, in turn, could negate the application of material laws that assume a uniaxial homogeneous stress-strain state in the evaluated areas. By applying Saint-Venant’s Principle (de Saint-Venant, 1853; Toupin, 1965), these loading effects from clamping become remote at a large enough distance away from the clamped ends, resulting in an almost homogeneous stress-strain state in the middle of the sample. This distance or region is referred to as the characteristic decay length (CDL) of a sample. Soft tissues are described as materials that exhibit anisotropic and/or hyperelastic behavior (Fung, 1993), and anisotropic materials tend to have considerably longer CDLs versus isotropic materials (Arridge and Folkes, 1976; Horgan and Simmonds, 1994). For example, a high-strength carbon-epoxy composite tested along the fiber direction exhibits a decay length roughly four times larger than an ideal isotropic material like steel (Horgan, 1982). Thus, by choosing sample dimensions that permit complex stresses to dissipate over a large enough distance, a uniaxial stress-strain state in the gauge section of a sample can be assumed (Horgan and Simmonds, 1994).

In addition to clamping effects, several factors can influence the ability to determine the true uniaxial stress-strain behavior of soft tissues. Some of these factors include: method of determining sample thickness and/or cross-sectional area (CSA) in the gauge section of the sample; quantification of lateral contraction (in-plane and out-of-plane of the loading direction); defining the starting reference configuration (e.g., after pre-conditioning); method of strain determination (i.e., local versus global); and time- and history-dependent response of the sample during loading. All these factors can impact the reconstruction of the mechanical behavior of a sample, and, in turn, the homogeneity of the stress field in the central cross-section under tensile loading, which may relate to sample dimensions in different ways. As such, it is important to be as thorough and complete in reporting the mechanical testing parameters of soft tissues, in order to enhance reproducibility and comparability of the extrapolated material properties.

Some experimental studies have explored the influence of sample geometries and/or clamping conditions on the measured material parameters derived from uniaxial and biaxial tensile testing of human soft tissues (Waldman et al., 2002; Waldman and Michael Lee, 2002; Sun et al., 2005; Sichting et al., 2015; Fischer et al., 2020; Firminger and Edwards, 2021; Wale et al., 2021). Under uniaxial tension, sample geometries of the aspect ratio (AR), which is the ratio of gauge length to width at the mid-section of a sample, and CSA demonstrated a significant effect on the measured material properties. For tendons, other properties such as the failure strain and failure stress were also influenced by different sample geometries and clamping conditions, rendering a comparison of the derived material properties futile, and as such, these testing parameters were viewed as crucial aspects to define in the testing protocols (Haut, 1986; Jimenez et al., 1989; Legerlotz et al., 2010; Hayes et al., 2019; Zwirner et al., 2020b; Wale et al., 2021). For soft tissues matrix-rich in collagen, discrepancies in the derived material properties were attributed to undetermined stresses introduced with clamping and to micromechanical factors such as the discontinuity of load-bearing fibrils (Anssari-Benam et al., 2012a; Anssari-Benam et al., 2012b; Peterson and Szczesny, 2020). While there have been tissue-specific studies that have aimed to examine the methods of measuring sample dimensions for mechanical testing (Griffin et al., 2016; Hayes et al., 2019; Zwirner et al., 2020c; Ge et al., 2020), a survey of the literature on what sample geometries and clamping conditions are reported in the uniaxial mechanical testing of human soft tissues has not yet been performed.

The aim of this review is to address this important and compelling challenge — the lack of standard test parameters for tensile testing of human soft tissues – through a systemic literature review using the Preferred Reporting Items for Systematic Reviews and Meta-Analyses (PRISMA) guidelines (Page et al., 2021a). PRISMA guidelines have grown in use with other disciplines outside of clinical studies, especially since its update in 2020 (Page et al., 2021b; Belle and Zhao, 2022; Kitchenham et al., 2023). Along with importance of reporting sample geometries and clamping conditions as described above, computational studies in soft tissue biomechanics are modelled from experimental data (and *vice versa*); as such, the need to report accurately and comprehensively basic testing parameters of sample geometry and clamping conditions becomes paramount, especially when deriving the material properties of a soft tissue sample. Also, the assertion that more comprehensive reporting leads to better outcomes is a well-understood principle in scientific research. Thus, in this PRISMA systematic review, we asked what sample geometries have been reported, whether clamping modifications have been involved, and how do the reported sample geometries compare against standards (e.g., International Organization for Standardization or ISO) for engineering materials that also exhibit anisotropic and/or hyperelastic behavior. We hypothesized that, for a given sample shape under uniaxial tensile loading, the reported ARs for human soft-tissue samples are significantly different and encompass a broader range of values in comparison to ISO standards. We then asked how these retrieved sample geometries might influence the stress distribution in a sample under uniaxial tensile loading. As an initial first-attempt to explore computationally how different sample geometries might influence the stress strain state in the middle of the gauge section of the sample under tensile loading, we performed Finite Element Analysis (FEA) using a constitutive model (Gasser et al., 2006) that we have previously described (Niestrawska et al., 2016). This model considers collagen fibril directionality and the material parameters of a sample and is valid for conditions such as little or no fiber dispersion. Modified versions of this model have also been used in soft tissues that include the aorta (Gasser et al., 2006), tendon (Akintunde and Miller, 2018), meniscus (Shriram et al., 2021), cartilage (Nolan et al., 2014), ligaments (Shearer, 2015), skin (Alliliche et al., 2023), cornea (Falgayrettes et al., 2023), and dura mater (De Kegel et al., 2018), amongst others. However, the use of this model in our FE simulations is not to suggest in any way that this approach is the definitive *in silico* method of choice to investigate human soft tissues samples under tensile loading. Rather, the choice of such a model for this pilot study is based on the aforementioned points, and on level of expertise of the user. Furthermore, there are several other constitutive modeling studies that consider collagen fiber-distributed soft tissues (Federico and Herzog, 2008; Cortes et al., 2010; Federico and Gasser, 2010; Pandolfi and Vasta, 2012; Vasta et al., 2014; Melnik et al., 2015; Gizzi et al., 2016; 2017; Holzapfel et al., 2019). Also, there are several other strain energy density equations to determine material parameters of biological tissues, as discussed in recent reviews (Holzapfel et al., 2019; Singh and Chanda, 2021; Arnold et al., 2023). As such, designing and performing a rigorous computational study that considers, for example, studies on collagen fiber-distribution in soft tissues is topic of future studies and beyond the scope of this review. Taken together, as an initial preliminary study, we performed FE simulations on a sample of little or no fiber dispersion at different sample geometries. We then elaborate on what sample geometries to consider reporting when performing uniaxial tensile testing of human soft tissues, along with other factors such as tissue-specific characteristics and clamping conditions. A list of testing parameters, towards standard reporting of the uniaxial testing parameters for human soft-tissue samples, is also proposed.

## 2 Methods

### 2.1 Systematic review using PRISMA guidelines and search strategy

A systematic review of the literature was conducted by observing PRISMA guidelines (Page et al., 2021a). This study is registered under the Open Science Framework or OSF (Registration Citation https://osf.io/8h7tb).

A search for articles was carried out in the databases of *PubMed*, *Web of Science*, and *BASE* (Bielefeld Academic Search Engine). The initial survey of the literature consisted of the following search terms:

(mechanic* OR material* OR biomechanic* OR biomaterial*) AND (propert*) AND

(tensil* OR uniaxial* OR quasistatic* OR anisotrop*) AND

(human* OR male* OR female* OR tissue* OR soft-tissue* OR cadaver* OR cadaveric OR postmortem* OR post-mortem* OR autopsy OR autopsie* OR biopsy OR biopsie* OR necropsy OR necropsie* OR dissection* OR dissect* OR donor* OR donation* OR donat* OR prosection* OR prosect* OR intraoperative* OR intra-operative* OR operation* OR operat* OR surger*)

A committee consisting of the co-authors was formed to further refine the search strategy. Two of the co-authors independently screened and identified the articles of interest based on search terms and selection criteria (see OSF, Registration Citation https://osf.io/8h7tb). Any disagreements were solved by consensus, or an independent third-party reviewer was consulted, if necessary.

Titles and abstracts were screened, and duplicates were removed using Endnote20 (Clarivate, United Kingdom) and Zotero software (Roy Rosenzweig Center for History and New Media, George Mason University, Fairfax, VA, United States). If the title or abstract did not provide sufficient information for screening, the full text of the manuscript was examined. All studies that met the selection criteria were included for full analysis and review.

### 2.2 Selection criteria

Studies were included if the following inclusion criteria were met:

i. Articles investigated human soft-tissue samples only.
ii. Articles investigated samples under uniaxial tensile testing.
iii. Articles published in English up until 31 December 2022.
iv. Full-text articles published in peer-reviewed journals.

For the inclusion criteria i), studies that involved a test sample that contained more than one (single) type of tissue (e.g., ligament along with its attachment to bone, tendon together with its muscle) were excluded. Records without abstracts, review articles, conference or meeting proceedings, and editorials were also excluded.

### 2.3 Extracted information from the included studies and ISO standards

**FIGURE 1:**
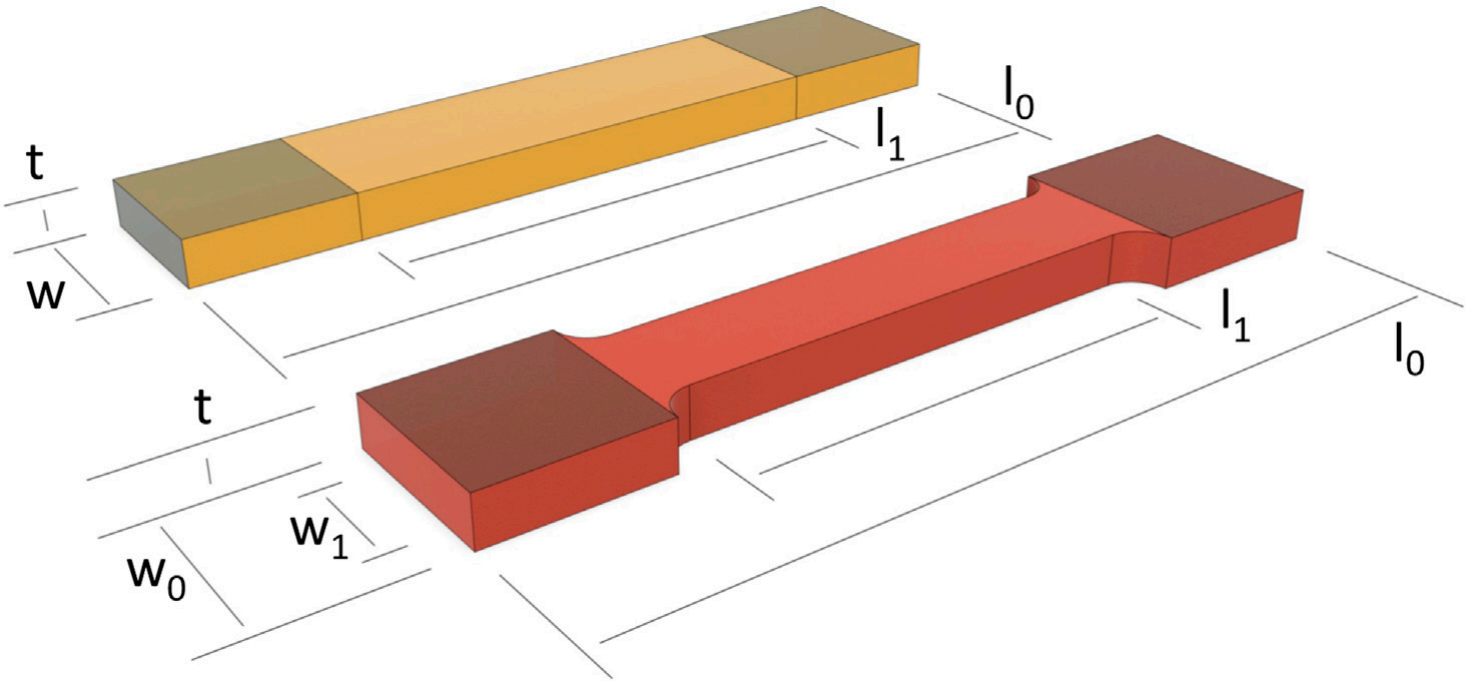
Sample dimensions for a non-tapered or rectangular (orange) and tapered or dog bone (red) sample shape. l_0_ = total length; l_1_ = length, gauge section; t = thickness; w = width, non-tapered shape; w_0_ = width, clamping area, tapered shape; w_1_ = width, gauge section, tapered shape. Clamping areas are shaded (top side), and the clamping regions are equal on both sides; as such, any difference is prescribed to a perspective effect only.

The following information was extracted, if available, from the included studies: first author’s last name; year of publication; type of human soft tissue tested; sample shape; sample dimensions of width (midsection), thickness (midsection), CSA (midsection), gauge length, and AR; and clamping modifications. For the AR of gauge length to width at the midsection of the sample, the width was considered the lesser of the two values, unless otherwise reported. Gauge length was recorded as the distance between the clamps for non-tapered samples (rectangle or strips) and the parallel length in the gauge section of tapered samples (dog bone or dumbbell). The geometries of both sample shapes are displayed in Figure 1.

The same information was also retrieved, if available, from ISO standards for anisotropic and/or hyperelastic engineering materials of polymers, composites, leather, and wood. The ISO standards were obtained from the *Perinorm* database (Beuth, Berlin, Germany).

### 2.4 Statistical analysis

Data retrieved on the sample geometries from the included studies were compared against ISO standards. Gaussian distribution was assessed, and a comparison between groups was performed using a Mann-Whitney *U* test. A *p*-value of 0.05 or less was considered statistically significant. All statistical analyses were carried out in PRISM 9.2.0 (GraphPad Software, San Diego, CA, United States).

### 2.5 Finite element analysis

Finite Element (FE) simulations of a soft-tissue sample undergoing uniaxial tensile loading were performed using FEBio 3.5.1 and FEBio Studio 1.6.1 (Musculoskeletal Research Laboratories, University of Utah, Salt Lake City, UT, United States, and Musculoskeletal Biomechanics Laboratory, Columbia University, New York, NW, United States) (Maas et al., 2012). The *Holzapfel-Gasser-Ogden* (HGO) model was used (Gasser et al., 2006), which was chosen to allow for incorporation of parameters pertaining to collagen fiber orientation (i.e., the in-plane fiber orientation angle γ and the fiber dispersion κ ) and of experimentally corroborated material parameters of the extracellular matrix (c, k_1_, and k_2_) (Niestrawska et al., 2016; Niestrawska et al., 2019). As a first attempt, we considered a sample of little or no dispersion. As such, γ was set to 1° and κ <0.05, with γ indicating two fiber families, each angled one degree away from the specimens’ long axis in the XY-plane and each corresponding to highly aligned fibers, resulting in strong anisotropy. FE simulations were also performed with γ set to 50° and κ <0.05, as previously described (Gasser et al., 2006; Fereidoonnezhad et al., 2020). Next, we sought to investigate whether a small change in the material parameters could influenced the choice of sample shape and sample geometry during tensile loading. As such, material parameters of c (i.e., stiffness of the ground matrix, in kPa) and k_1_ (i.e., stiffness of fibers, in kPa) were set to constant values; and k_2_ (i.e., stiffening of loading curves due to collagen fibers, dimensionless) was increased.

Each sample was discretized by structured linear hexahedral elements. The element edge length was approximately 0.5 mm, which corresponds to eight elements in thickness. The element edge length was determined by additional numerical experiments on the effect of mesh size on the stress norm. The equilibrium equations were then solved using a three-field numerical scheme suitable for incompressible materials. The incompressibility constraint was enforced using augmented Lagrange multipliers. Tapered and non-tapered samples were modelled and analyzed through a range of aspect ratios (i.e., from 1:1 to 10:1). For both sample shapes, the gauge length was considered as the free length between the gripping jaws.

**FIGURE 2:**
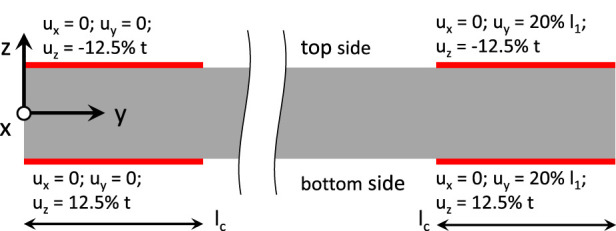
Boundary conditions for all samples modelled under Finite Element Analysis (FEA). A side view of the sample is shown, with the clamping surfaces depicted in red. l_c_ = clamping length; l_1_ = gauge length; u = displacement; and t = thickness.

The simulation, which involves computing the first principle Cauchy stress, was divided into two steps. First, in order to account for clamping at both ends of the sample, the FE simulations were performed with a prescribed reduction of thickness by 1 mm in the clamping areas, which resulted in a thickness of 3 mm or 75% of the initial thickness value of 4 mm. The second step involved increasing strain along the loading direction, which was achieved by moving one of the clamping areas such that the gauge length of the sample was increased by 20%. The clamping and strain conditions were selected based on previous studies (Wale et al., 2021). The implemented boundary conditions are shown in Figure 2. The dimensions of the sample shapes followed ISO standards (Standardization, I.O.f., 2012; Standardization, I.O.f., 2014; Standardization, I.O.f., 2016; Standardization, I.O.f., 2018; Standardization, I.O.f., 2020; Standardization, I.O.f., 2021a; Standardization, I.O.f., 2021b). The resulting distribution of the first principle Cauchy stress was computed and displayed as a color map on the surface. Through a cross-section in the middle of the gauge section, the ratio between the highest and lowest first principle stress was calculated and also displayed as a color map. The ratio between the highest and lowest first principle Cauchy stress allows for a comparison between samples of different sample geometries. FE simulations described above were also performed for both sample shapes on a compressible material by releasing the incompressibility constraints. Also, for a compressible material, FE simulations were also performed with a prescribed reduction of thickness by 0.1 mm in the clamping areas, which resulted in a thickness of 3.9 mm or 97.5% of the initial thickness value of 4 mm. A summary of the material parameters utilized in all the FE simulations are provided in Supplementary Tables S1, S2.

## 3 Results

**FIGURE 3:**
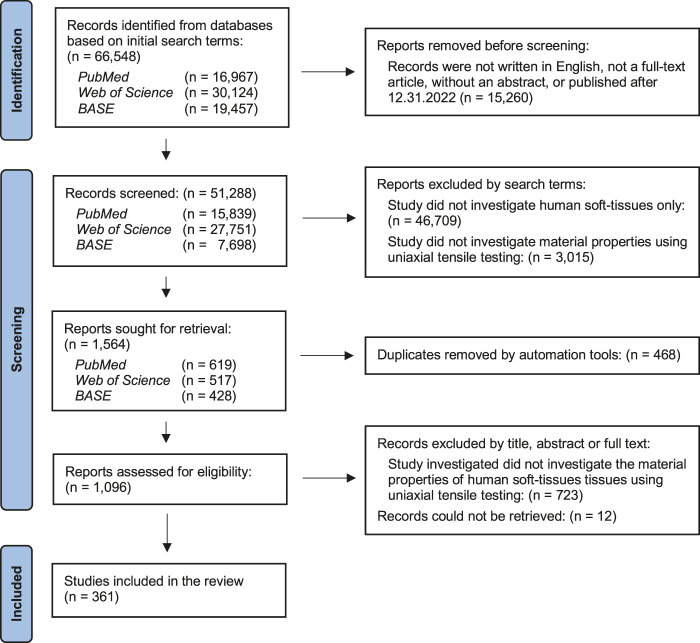
PRISMA flow chart on the screening and retrieval of studies.

A flow chart summarizing the literature search is shown in Figure 3. An initial survey of the three databases generated a total of 66,548 articles. Articles were removed based on the inclusion criteria as described above, resulting in 50,739 records for further screening. After exclusion of reports based on search terms and removal of duplicates, 1,096 articles were assessed for eligibility. A further 723 articles did not meet the inclusion criteria and 12 articles could not be retrieved. In total, the final number of included studies was 361.

A total seven ISO standards were included for engineering materials of polymers (ISO 527-2/-3) (Standardization, I.O.f., 2012; Standardization, I.O.f., 2018), composites (ISO 527-4/-5, ISO 1421) (Standardization, I.O.f., 2016; Standardization, I.O.f., 2021a; Standardization, I.O.f., 2021b), leather (ISO 3376) (Standardization, I.O.f., 2020), and wood (ISO 13061-6) (Standardization, I.O.f., 2014).

The retrieved data from the 361 articles and seven ISO standard materials can be found in Supplementary Table S3–S8.

### 3.1 Predominant sample shapes were tapered and non-tapered

Non-tapered (i.e., rectangular or strips) and tapered (i.e., dog bone or dumbbell) were the two most common sample shapes described, representing 56.0% (202 of 361) and 23.6% (85 of 361) of the included studies respectively (Supplementary Tables S3, S4). Seven studies or 1.9% reported both tapered and non-tapered shapes (Supplementary Table S5). A single edge notched tensile bar, a wedge shape, a square, and a tube shape were each reported once, and 16 studies reported a cylinder, for a total of 20 studies or 5.5% (Supplementary Table S6). The remaining 47 studies (13.0%) did not specify the sample shape (Supplementary Table S7).

From the seven ISO standards, three materials (i.e., polymer, leather, and wood) specified a tapered sample shape (Standardization, I.O.f., 2012; Standardization, I.O.f., 2014; Standardization, I.O.f., 2018; Standardization, I.O.f., 2020), three composite materials specified a non-tapered sample shape (Standardization, I.O.f., 2016; Standardization, I.O.f., 2018; Standardization, I.O.f., 2021a; Standardization, I.O.f., 2021b), and one material (i.e., polymer) included a description of both shapes (Standardization, I.O.f., 2018) (Supplementary Table S8).

### 3.2 Reporting of sample dimensions varied amongst the included studies

Amongst the studies that reported a tapered sample shape, over two-thirds (67 of 92) of the articles provided the ARs and/or details of width and gauge length to calculate AR (Supplementary Tables S4, S5). However, for studies reporting the use of a non-tapered sample shape, these dimensions were seldom described, reported in only 87 of 210 articles (Supplementary Tables S3, S5). Thus, only 154 of 361 studies reported both sample shape and AR dimensions in the gauge section. Several of these studies also reported more than one AR. Overall, for a tapered sample shape, a total of 93 ARs were retrieved, ranging from 0.84 to 13.65; and for a non-tapered sample shape, a total of 125 ARs were retrieved, ranging from 0.20 to 11.86.

For the ISO standard materials, 12 ARs were retrieved amongst the 4 engineering materials using a tapered sample shape, and nine ARs were retrieved from the 4 materials using a non-tapered sample shape (Supplementary Table S8). Overall, the ARs ranged from 1.97 to 7.50 for a tapered sample shape and 3.00 to 10.00 for a non-tapered sample shape.

**FIGURE 4:**
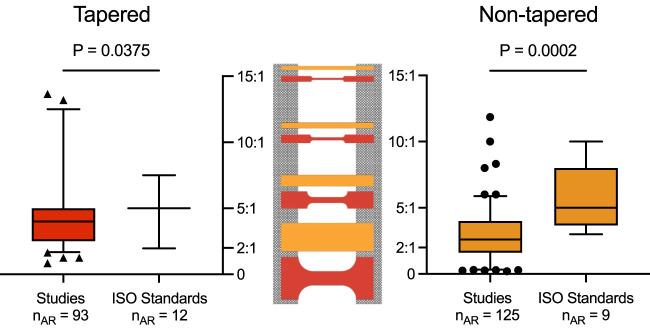
Aspect ratios or AR (*y*-axis) for tapered (red, left) and non-tapered (orange, right) sample shapes retrieved from the included studies and ISO standards. Box plots show the median and the 25th and the 75th percentiles. Whiskers represent the 5th to 95th percentiles, with outliers depicted as symbols (circle or triangle). For both sample shapes, there was a significant difference in the mean ARs between the included studies and ISO standards (i.e., for tapered, 4.57 versus 4.94, *p* = 0.0375; and for non-tapered, 2.94 versus 5.81, *p* = 0.0002). ARs are shown schematically for both samples (center) with the clamping area (grey) for ARs of 15:1, 10:1, 5:1, and 2:1 (top to bottom).

A comparison between the ARs from the included studies and the ISO standards revealed a significant difference in the mean for both tapered (4.57 versus 4.94, *p* = 0.0375) and non-tapered (2.94 versus 5.81, *p* = 0.0002) sample shapes (Figure 4). Of note, the other retrieved sample geometries were underreported and sporadic amongst the included studies (see Supplementary Tables S3–73); as such, no further considerations for comparison could be made.

### 3.3 Clamping modifications reported in over two-fifths of the included studies

Over two-fifths of the studies (156 of 361 or 43.2%) reported sample clamping modifications in their experimental setup (see Supplementary Tables S3–S5). The most common modifications were the use of sandpaper and glue, found in 68 and 58 studies respectively. Polymer inserts with pyramids to grip onto the tissues were reported in 10 studies; eight studies used cloth or gauze; seven used paper; seven used tape (adhesive or hook-and-loop); seven used cryoclamps; four studies partially plastinated the ends of their samples; six used rubber and/or roughened/toothed inserts in the clamping jaws; and six used sutures. There was no statistically significant correlation between the use of clamping modifications and sample shape or dimensions of AR.

### 3.4 Greater stress inhomogeneity with smaller ARs on FEA of aligned fiber samples?

**FIGURE 5:**
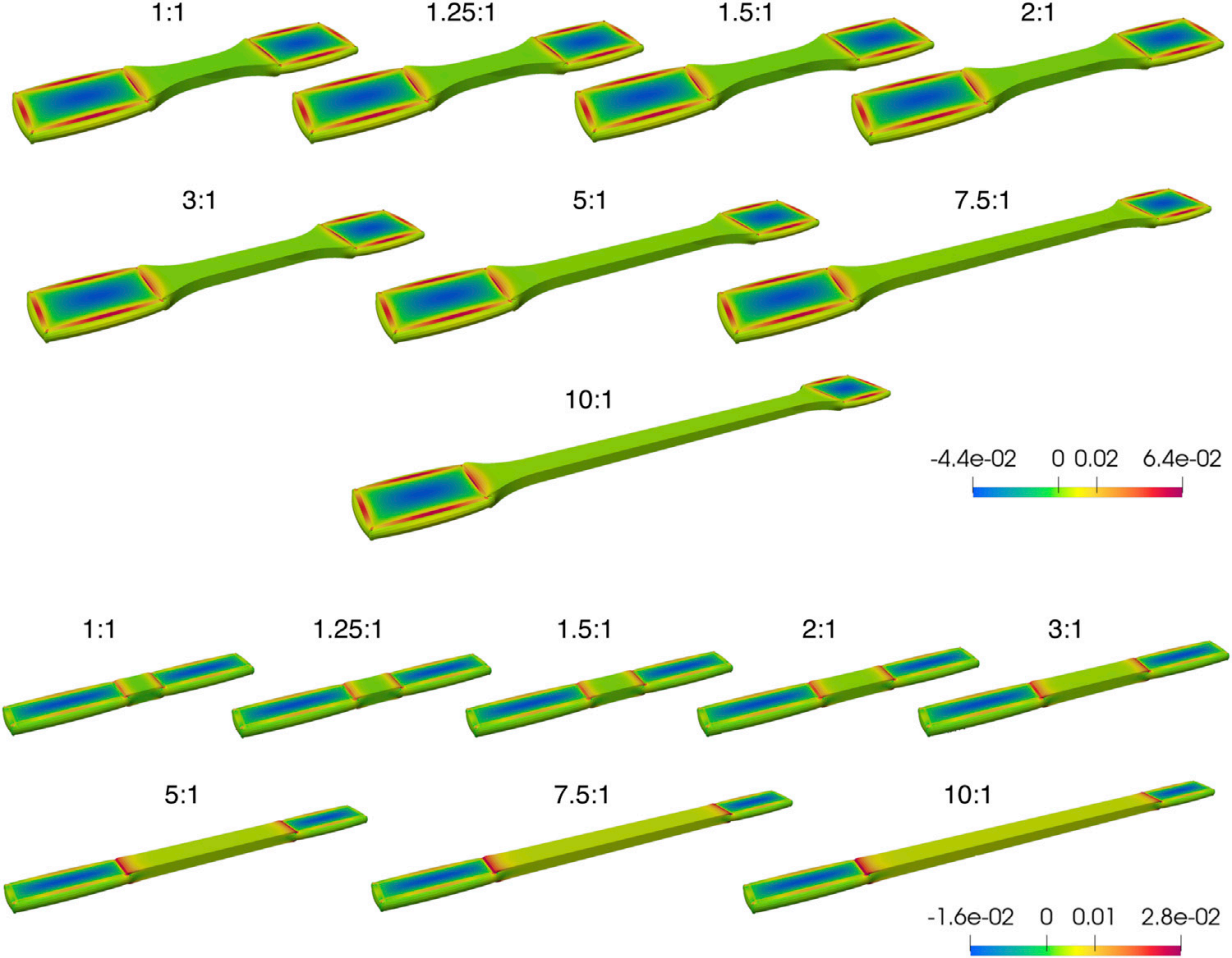
Overview of the first principle Cauchy stress distribution from Finite Element Analysis (FEA) of incompressible samples at a 20% strain in the gauge section and a 25% reduction in thickness in the clamped regions, for samples of little or no fiber dispersion. Although the clamping regions are deformed, the undeformed state (i.e., the state of the sample without any further longitudinal displacement from tensile loading) is shown, at a range of aspect ratios for tapered (above) and non-tapered (below) sample shapes. The color scale bar represents the range between the largest and smallest first principle Cauchy stress (N/m^2^). Clamping regions are equal on both sides; as such, any difference is prescribed to a perspective effect only.

**FIGURE 6:**
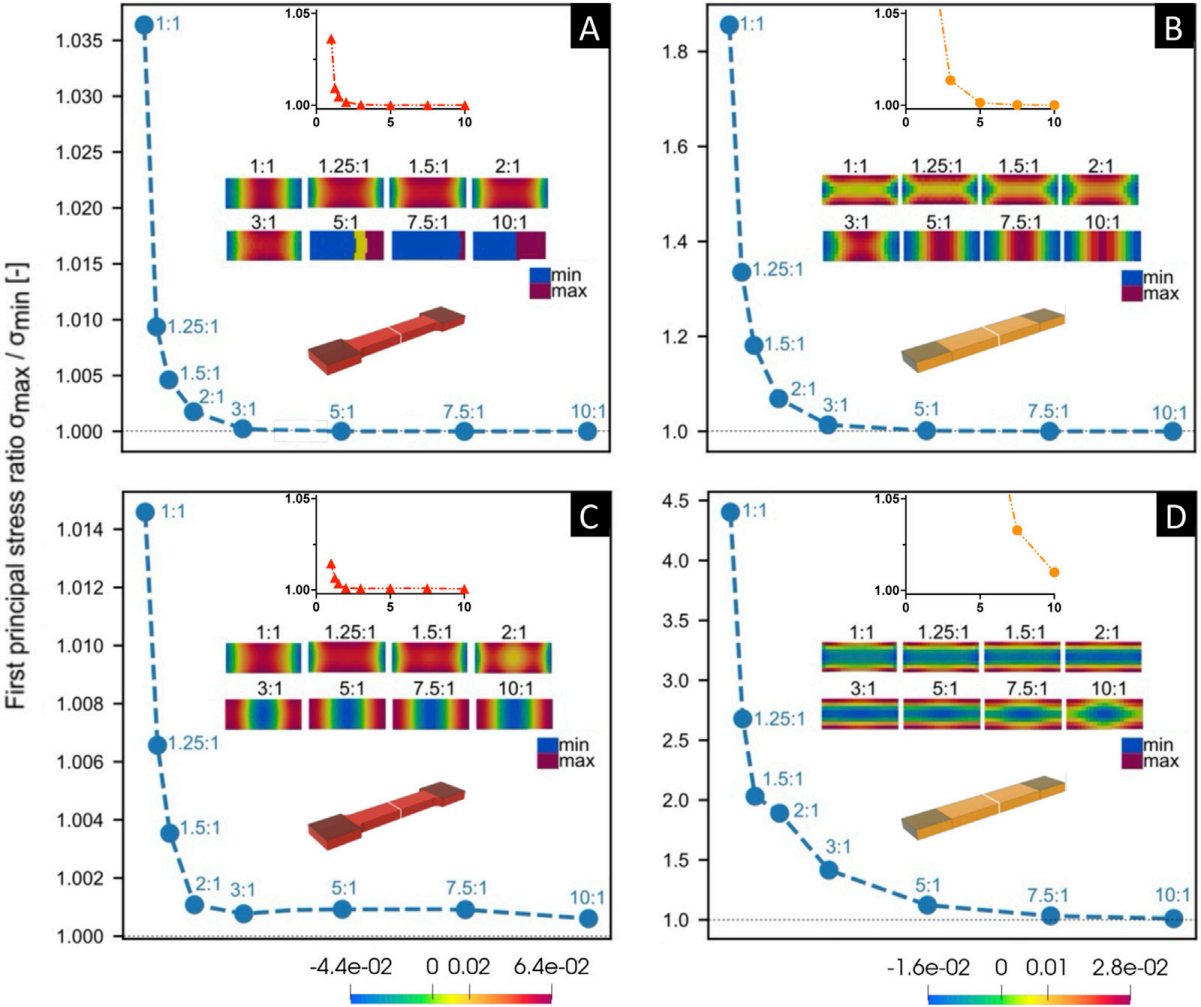
Comparison of the aspect ratio (*x*-axis) versus fold-difference in the first principle Cauchy stress (*y*-axis) through a cross section at the middle of the gauge section of the sample (shown in white) via Finite Element (FE) simulations, for both tapered [left, **(A,C)**] and non-tapered [right, **(B,D)**] sample shapes. FE simulations were performed on incompressible samples of little or no fiber dispersion and fiber mean orientation angle γ was set at *γ* = 1°. Samples were kept at 20% strain in the gauge section and a 25% reduction in thickness in the clamped regions. Note the range in *y*-axis values differ in the larger plots and are the same in the close-up, smaller plots. Material parameters of c, k_1_, and k_2_ were kept constant for both sample shapes [above, **(A,B)**], and then k_2_ was increased for both sample shapes [below, **(C,D)**]. The color scale bar (see Figure 5) represents the range between the largest and smallest first principle Cauchy stress (N/m^2^).

In our preliminary pilot study, FE simulations performed for both tapered and non-tapered sample shapes under tensile loading within a range of the retrieved ARs are summarized in Figures 5, 6. A 3D overview of the first principle Cauchy stress in a sample is shown in Figure 5, and the stress distribution through a cross-section in the middle of the gauge section of a sample is shown in Figure 6. Tensile loading conditions were prescribed at a 20% strain increase in the gauge section in the undeformed state (i.e., the state of the sample without any further longitudinal displacement from tensile loading) and a 25% reduction of thickness in the clamping regions (Figure 5). Also, for both shapes, two sets of material parameters were explored that differed in one material parameter of k_2_.

Two general observations were noted. First, the clamping area extends over a greater length at lower ARs for both sample shapes (Figure 5), which is more pronounced for a non-tapered sample shape (e.g., at an aspect ratio AR = 1:1 in Figure 5). Second, for both sample shapes, across the middle of the gauge section, the fold-difference between the highest and lowest stress follows an exponential decay with increasing ARs (Figure 6).

Upon closer examination at two slightly different sets of material parameters, differences were noted between sample shapes. For a given set of the same material parameters (Figures 6A, B), the fold-change in stress approaches 1 at increasing aspect ratios of AR>3:1 for both a tapered sample (Figure 6A) and non-tapered sample (Figure 6B). However, by increasing material parameter k_2_ (Figures 6C, D), this fold difference approaches 1 at increasing aspect ratios of AR>5:1 for a tapered sample shape (Figure 6C) and AR>10:1 for a non-tapered sample shape (Figure 6D). Thus, with this increase in k_2_, there is a greater fold-change in stress at smaller ARs for both sample shapes but is more pronounced for a non-tapered sample shape (e.g., at an aspect ratio of AR>2:1 in Figure 6B vs. Figure 6D).

Of note, the cross-sections in Figure 6 are representative outcomes of the FE simulations performed, which can result in generating a color map in discontinuity. In Figure 6A, although the color map is not continuous for AR>5:1, the actual difference between the largest and smallest first principle Cauchy stress values is near zero (as evidenced by a ratio of essentially 1).

**FIGURE 7:**
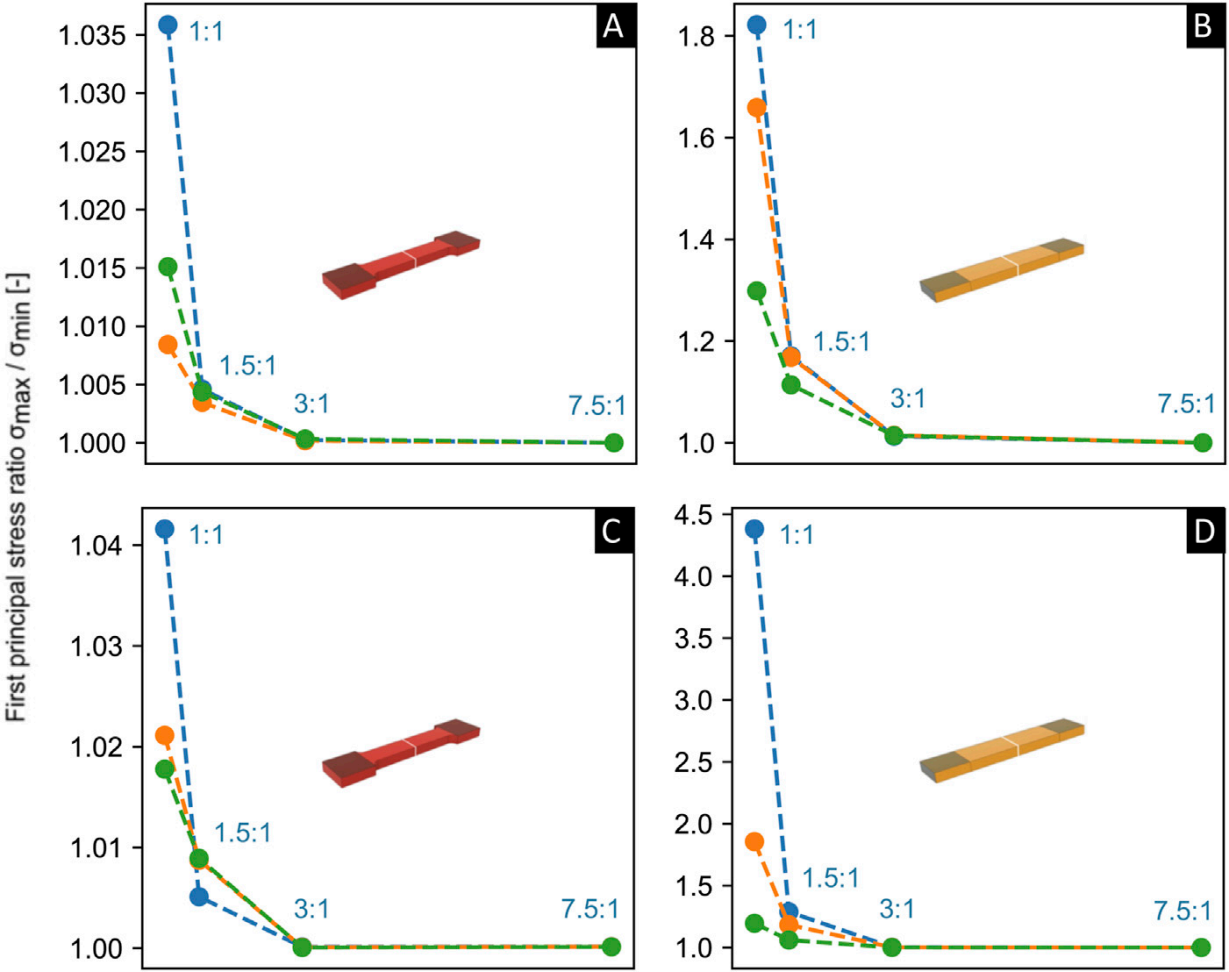
Comparison of the aspect ratio (*x*-axis) versus fold-difference in the first principle Cauchy stress (*y*-axis) through a cross section at the middle of the gauge section of the sample (shown in white) via Finite Element (FE) simulations, for both tapered [left, **(A,C)**] and non-tapered [right, **(B,D)**] sample shapes. FE simulations were performed on samples of little or no dispersion and fiber mean orientation angle γ was set at γ = 1° [top, **(A,B)**] and γ = 50° [below, **(C,D)**]. Material parameters of c, k1, and k2 were kept constant and were identical to the parameters utilized in Figures 6A, B). Samples were kept at 20% strain in the gauge section and at a 25% reduction or 2.5% reduction in thickness in the clamped regions. Incompressible sample, 25% thickness reduction in blue; compressible sample, 25% thickness reduction in orange; compressible sample, 2.5% thickness reduction in green.

For one set of the same material parameters ascribed in Figures 6A, B, we also explored in a limited study other parameters of compressibility with or without reduced clamping deformation (Figures 7A, B), and at an increase in fiber mean angle orientation of γ = 1° (Figures 7A, B) versus γ = 50° (Figures 7C, D). The same results of the FE simulations for an incompressible sample in Figures 6A, B are also included (in blue) in Figures 7A, B for ease of reference. For a sample of little or no fiber dispersion (i.e., κ <0.05), the fold difference approaches 1 at increasing aspect ratios of AR>3:1 for both a tapered (Figures 7A, C) and a non-tapered sample shape (Figures 7B, D)).

## 4 Discussion

In this systematic review, sample geometries and clamping parameters for tensile testing of human soft tissues were investigated using PRISMA guidelines. Sample shape and dimensions in the gauge section are often required to assess the stress-strain state of a sample during uniaxial tensile testing. We found that over 80% of the included studies (295 out of 361) described either a non-tapered or tapered sample shape, or both. However, about 40% (148 of 361) specified both sample shape and sample dimensions of AR in the gauge section. In contrast, all ISO standards of engineering materials included in this review reported all sample geometries in the gauge section and testing conditions. Clamping modifications were reported in over 40% of the included studies, although there was no correlation found with sample shape or AR.

### 4.1 Sample geometries from the included studies differ from ISO standards

Soft tissues are described as incompressible and exhibit anisotropic and/or hyperelastic behavior (Fung, 1993). Since no standard testing protocols exist for human soft-tissue samples, sample geometries retrieved from the included studies were compared against ISO standards for tensile testing of anisotropic and/or hyperelastic engineering materials. There was a significant difference found between ARs from the included studies and ISO standards for both tapered and non-tapered shapes. At first glance, this difference could simply be amounted to the inherent differences in composition of human soft tissues versus engineering materials. However, a closer look and comparison between aspect ratios raises some interesting considerations. As aforementioned, tensile testing of anisotropic materials, in general, require a longer CDLs in comparison to isotropic materials (Arridge and Folkes, 1976; Horgan and Simmonds, 1994). Some of the included ISO standard engineering materials also exhibit isotropic properties. As such, in comparison to isotropic materials, tensile testing of human soft-tissue sample of a given sample shape should require a higher AR (Zwirner et al., 2020c). However, for both tapered and non-tapered sample shapes, the retrieved ARs were smaller for human soft-tissue samples in comparison to the ISO standard materials. This discrepancy might also be explained by extrinsic factors that could influence the mechanical properties of a soft-tissue sample, such the influence of water content (Lozano et al., 2019), chemical embalming (Steinke et al., 2012; Zwirner et al., 2019) and surface coating (Zwirner et al., 2020a). Along with differences in experimental setups, a minimal AR to facilitate a uniaxial stress-strain state in the gauge section would not be the same for all tissues. Therefore, the retrieved sample dimensions and mean ARs from the included studies should not be considered as absolute, but rather treated with caution.

### 4.2 Could sample geometries impact tensile stress distribution on FEA of aligned fiber samples?

The preliminary results from our pilot study using FE simulations shown herein provide a possible hypothesis on the stress distribution in the middle gauge section during uniaxial tensile loading in a sample of little or no fiber dispersion, for two different sample shapes at two sets of soft-tissue material parameters of c, k_1_ and k_2_ that differed only in k_2_. For a tapered sample shape, the fold-difference in stress through a cross section in the middle gauge section was found to be negligible (i.e., a fold change towards 1) at aspect ratios of, e.g., AR>5:1 with either set of material parameters of c, k_1_ and k_2_. However, for a non-tapered sample shape, an increase in one of the material parameters of k_2_ resulted in a larger aspect ratio of AR>10.1, in order to achieve a negligible fold-difference in stress. Thus, based on this preliminary pilot study on a sample of little or no fiber dispersion, for tapered and non-tapered sample shapes of samples with the same starting thickness and width in the clamping area (i.e., w = w_0_ as defined in Figure 1), one hypothesis could be that a greater length in the clamping area could possibly be required for a non-tapered sample shape in order to achieve the same change in gauge length in the undeformed state. Also, a limited study into other parameters of compressibility, decreased clamping deformation, and/or increased fiber mean orientation angle γ might suggest the hypothesis that the fold-difference in stress through a cross section in the middle gauge section approaches 1 at aspect ratios of AR>5:1 (and perhaps even for AR>3:1).

### 4.3 Limitations of the current pilot FE studies and considerations for future FE simulations

As aforementioned, the rationale behind and the results obtained from our FEA pilot study should not be interpreted as definitive nor conclusive. In the FE simulations shown herein, only two sets of slightly different material parameters were selected for a sample of little or no fiber dispersion; as such, the results cannot be extrapolated to account for all different soft-tissue types of, e.g., cartilage, skin, dura matter and cardiovascular tissues. Also, clamping could impose a strong compression force not represented by the constitutive law which is only valid in the tensile state and at low dispersion. As such, experimentally, clamping modifications (e.g., sinusoidal-shaped or serrated jaws, sandpaper) are employed to increase the contact area of the clamp across the sample (Lake et al., 2023). Furthermore, incompressibility as a characteristic of soft tissues in computational biomechanics is not valid if the sample becomes dehydrated during an experiment. For a dried and dehydrated sample that is quasi-incompressible, a porous medium approach could be considered. However, measures are undertaken experimentally to keep samples moist and hydrated prior to and during testing, with accurate and efficient loading and alignment of the sample into the testing device (Scholze et al., 2020). As such, incompressibility has been assigned during computations. In our pilot study, the clamping parameters were designed such that compression in the clamping region is applied in the direction perpendicular to the tensile load and longitudinal orientation of fibers in the sample. Also, we did not prescribe any kinematic conditions at the outside edges of the sample that could result in physical unloading of the interior fibers from clamping forces. There are computational studies whereby the clamping region is not considered, and Dirichlet displacement boundary conditions could be used to bypass such a scenario. Although a rigorous computation study (or a design of such a study) is beyond the scope of this PRISMA systematic review, we note that any future computational study should also consider a range of material parameters (e.g., fiber and matrix stiffness) as to avoid unphysical behaviors (e.g., auxetic behavior) that can arise from hyperelastic anisotropic constitutive models of soft tissues (Fereidoonnezhad et al., 2020; Morin et al., 2021). Nevertheless, since computational study studies aim to realistically reproduce the experimental tests, accurate and thorough reporting of experimental testing parameters (e.g., sample geometry and clamping conditions) are relevant when determining the material properties of a soft tissue sample through mechanical testing.

### 4.4 Tapered versus non-tapered–which sample shape and size to choose?

For tensile testing of human soft-tissue samples, there are some factors to consider when choosing between a tapered versus a non-tapered sample shape. For our pilot study using FEA simulations, we considered a sample that exhibited hyperelastic behavior and of little or no fiber dispersion. In this scenario, for a gauge section of the same width and thickness, a tapered sample shape would require less overall length and a wider amount of starting material (to accommodate broader clamping ends) in comparison to a non-tapered sample shape. In general, with a non-tapered shape, individual testing samples can be derived in succession by stamping out adjacent strips, thereby maximizing use of the tissue sample. However, when testing until failure, clamping-induced stresses pose a problem in stress distribution, especially towards the clamped ends. For non-tapered samples, the stresses around the clamps often surpasses the stress in the midsection in magnitude and complexity (Horgan and Simmonds, 1994), which was also observed through the FE simulations at different ARs. Thus, if the AR and/or clamping area of a sample is inadequate during tensile loading, failure of the sample is more likely to occur within the unknown high-stress regions at the clamps. As aforementioned, the distance between clamps needs to accommodate a sufficient CDL so that the stresses from clamping can dissipate and allow for failure in the gauge section of a sample. In other words, for a given CDL, failure in the mid-section of a sample from tensile loading can be achieved by increasing the AR and thereby reducing the CSA in the gauge section. The forces required to pull the sample apart would act across a smaller CSA, and as such, the differences in stress in the gauge section would be greater than the stresses around the clamps, thereby promoting failure in the midsection. In essence, this reduction in the CSA by increasing the AR describes the intention of a tapered sample shape–a tapering of the sample in the gauge section reduces the CSA in the midsection with wider ends in the clamping area. Therefore, while a non-tapered sample may result in a more efficient use of tissue by minimizing tissue waste, tapered samples would favor greater stress homogeneity and propensity for failure in the gauge section under tensile loading. However, in this oversimplified view of a hyperelastic material, perhaps a tapered geometry could be advocated. In reality, deformation of soft tissues are time- and history-dependent, and the rate of deformation is related to the amount of the stress applied in a non-linear manner. As such, global (nominal) strains (i.e., change in length along the entire sample) and local strains (i.e., change in length in the gauge section) would differ more for a tapered geometry. If a relaxation phase follows the loading phase in a uniaxial test, likely the global (and not the local) strain will be kept constant. For a tapered sample, the result will be a non-zero strain rate in the different regions of the sample. Furthermore, the use of a tapered shape requires a local strain measurement and test control based on this local readout (e.g., through digital image correlation), which is absent in most cases. Taken together, the choice between a tapered and non-tapered sample is not straight-forward and a recommendation for one shape over the other is beyond the scope of this review.

### 4.5 Experimental setup with clamping modifications could help with inadequate ARs

The amount of starting material and the availability of human tissue samples are often limited, and as such, higher ARs may be difficult to achieve for a given CDL and sample shape. By enhancing friction at the interface between the clamps and the tissue, some issues associated with clamping (e.g., a more complex stress state at the clamped ends) can be attenuated. As such, the clamping force, as well as the area of clamped tissue, can be set a lower threshold whilst still maintaining a sufficient tensile load across the sample during tensile loading. Also, with a smaller area of clamped tissue, a greater amount of the sample would potentially be available to achieve a larger AR for a given sample shape. As such, the result would be greater homogeneity in the stress-strain state in the gauge section of the sample. The most common methods to enhance the friction include the use of sandpaper, cyanoacrylate glue, partial plastination, or additively manufactured serrated clamps. Most of these methods are inexpensive and easy to implement and therefore should be considered for tensile testing of human soft-tissue samples.

## 5 Conclusion

Mechanical testing for the material properties of human soft tissues continues to gain wide appeal for its application in related fields of bioengineering, biomaterials, and in health and disease. Unlike for engineering materials, standardized testing parameters are not available for human soft-tissue samples. Using PRISMA guidelines, our systematic review showed that over 80% of the included studies reported the use of a standardized sample shape of non-tapered and/or tapered. However, sample dimensions, especially in the gauge section for deriving material properties, were underreported, with about 40% of the studies providing details on both the sample shape and aspect ratio. Furthermore, over 40% of the included studies reported the use of clamping modifications, which may account for the lower aspect ratios of AR<5:1 that were retrieved from the included studies. As a first-attempt pilot study using FEA, we explored how samples geometries might influence the stress distribution across the midsection of a sample of little or no fiber dispersion. The preliminary FE simulation results might suggest the hypothesis that different sample geometries could impact the stress-distribution under tensile loading. However, no conclusions can be drawn from these simulations, and future studies should involve exploring different testing parameters under different computational models and sample parameters (such as fiber dispersion and clamping effects). Along with the variety of soft-tissue types and differences in experimental setups, the choice of which sample shape and clamping conditions to employ in tensile testing of human soft tissues remain elusive. Nevertheless, given the variety and underreporting of parameters shown herein, accurate and comprehensive reporting of testing parameters should be adopted in future experimental and computational studies.

### 5.1 Towards standard reporting of parameters in tensile testing of human soft tissues

Taken together, we suggest a simple metric of reporting testing parameters when performing uniaxial tensile testing of human soft tissues (Table 1). In brief, three general categories of information are proposed: Tissue, Sample, and Experimental Setup.

**TABLE 1:** Proposal towards standardized reporting of parameters in uniaxial testing of human soft-tissue samples.

Tissue
- Type of soft-tissue, anatomical location and name, other tissue characteristics
- Source of tissue
- Number of tissue donors
- Age of tissue donor(s)
- Number of each tissue-type retrieved from each tissue donor
- State of tissue for testing
- ICD codes of the tissue donor
Sample
- Sample shape (with method of stamping)
- Number of samples tested (for each sample shape)
- Overall length and width of a sample (with measurement method)
- Gauge length, gauge width and thickness at mid-section (with measurement method)
- AR and CSA at the gauge section (with measurement method)
- Clamping area (with measurement method and any modifications)
Experimental Setup
- Clamping method or type of clamp
- Clamping modifications
- Measurement methods
- Testing conditions
- Testing machine

For tissue specifics, these details would include the type of tissue (e.g., connective tissue, muscle), its anatomical location and name (e.g., leg, semitendinosus muscle), and other tissue characteristics (e.g., normal or unaffected versus pathologic); the source of the tissue (e.g., biopsied, operative, autopsied); the total number of tissue-donors; the age of tissue donors at time of retrieval (e.g., ages 55–74); the total number of each tissue-type retrieved from each tissue donor (e.g., two patellar tendons and two medial menisci of the knee retrieved from each of the 10 tissue donors, for a total of 20 patellar tendons and 20 medial menisci); and state of the tissue for testing (e.g., fresh or intact, frozen then freeze-thawed, preserved with method of fixation or chemical processing). If possible and under the appropriate ethical considerations, International Classification of Diseases or ICD codes (for the current ICD version, see https://icd.who.int/en) ascribed to the tissue donor could also be included.

For sample specifics, details would encompass the sample shape (e.g., tapered or dog bone) and method of achieving the shape (e.g., scalpel, scissors, die cutting); the total number of samples tested for each sample shape; and sample dimensions of overall length, width and thickness, gauge length, gauge width, and thickness at the middle of the gauge section; AR and CSA and in the gauge section; clamping area and if/any modifications to the clamped ends of the tissue (e.g., plastinated ends, sutures, cloth, paper, sandpaper) would also be noted.

For experimental setup, details would encompass information on the clamping method or type of clamps (e.g., self-tightening grips, pinching, webbing grips, rope and thread grips, hydraulic grips, wedged grips, pneumatic grips, manual vice grips; cryoclamps); clamp modifications (e.g., glue, sandpaper, rubber, serrated-pyramid); measurement methods (e.g., ruler, calipers, digital image correlation, laser); testing conditions (e.g., samples tested in air, in solution, under temperature control); and testing machine (e.g., original design or commercially available). Overall, such details would provide an important framework towards standard reporting of testing parameters used in tensile testing of human soft-tissue samples, thereby improving repeatability, reproducibility, and comparability of the results.

To conclude, it is worth noting that the findings from the systematic review presented herein, however intuitive or rudimentary they may (or may not) seem, could not have been accomplished without a rigorous and thorough survey of the literature, which was achieved by observing PRISMA guidelines. The advocacy for a set of criteria or standards in reporting for peer-reviewed publications is not novel (Munafo et al., 2017). Furthermore, journals such as *Nature* have their own mandatory reporting summary templates (https://www.nature.com/documents/nr-reporting-summary-flat.pdf). The above proposal on which testing parameters to report in future studies should not be view as absolute but should serve as a starting point towards standards and practice guidelines. In fact, such an endeavor necessarily requires further input from those who are engaged in experimental and/or computational studies in the mechanical testing of human soft tissues.

## Data Availability

The original contributions presented in the study are included in the article/Supplementary Material, further inquiries can be directed to the corresponding authors.
