# Supplementary material for: Shape or size matters? Towards standard reporting of tensile testing parameters for human soft tissues: systematic review and finite element analysis
Source: Front Bioeng Biotechnol. 2024 Mar 26;12:1368383. doi: 10.3389/fbioe.2024.1368383 (PMC11005100; doi:10.3389/fbioe.2024.1368383)
Supplement: Supplementary file 2 [file Table1.pdf]

Supplementary Table 1

Chosen modelling parameters in the Finite Element Analysis (FEA) for a both a non-tapered (rectangular) and tapered (dogbone or dumbbell) sample shape (see Figure 6(A)and 6(B), and Figure 7). κ<0.05 indicates highly aligned fibers, and γ=1° or γ=50° relative to the specimen long axis indicates two fiber families angled one degree or 50 degrees away from the centerline in the XY-plane respectively.

**c** **[kPa]**	**k_1** **[kPa]**	**k_2** **[-]**	**κ** **[-]**	**γ** **[°]**
3.77	0.366	7.18	0.01	1
3.77	0.366	7.18	0.01	50
