# Supplementary material for: Shape or size matters? Towards standard reporting of tensile testing parameters for human soft tissues: systematic review and finite element analysis
Source: Front Bioeng Biotechnol. 2024 Mar 26;12:1368383. doi: 10.3389/fbioe.2024.1368383 (PMC11005100; doi:10.3389/fbioe.2024.1368383)
Supplement: Supplementary file 3 [file Table2.pdf]

Supplementary Table 2

Chosen modelling parameters in the Finite Element Analysis (FEA) for a both a non-tapered (rectangular) and tapered (dogbone or dumbbell) sample shape (see Figure 6(C) and 6(D)). $\boldsymbol{\kappa}$<0.05 indicates highly aligned fibers and a γ=1° relative to the specimen long axis indicates two fiber families angled one degree away from the centerline in the XY-plane.

**c** **[kPa]**	**k_1** **[kPa]**	**k_2** **[-]**	**$\boldsymbol{\kappa}$** **[-]**	**$\boldsymbol{\gamma}$** **[°]**
3.77	0.366	25.00	0.05	1
