# Supplementary material for: Shape or size matters? Towards standard reporting of tensile testing parameters for human soft tissues: systematic review and finite element analysis
Source: Front Bioeng Biotechnol. 2024 Mar 26;12:1368383. doi: 10.3389/fbioe.2024.1368383 (PMC11005100; doi:10.3389/fbioe.2024.1368383)
Supplement: Supplementary file 4 [file Table3.pdf]

Supplementary Table 3

Included studies using a non-tapered sample shape, arranged by publication year and last name of the first author. All retrieved measurements are from the gauge section of the sample. Aspect ratios and clamping modifications are highlighted (light and dark grey respectively).

First Author, Last name	Year	Human Soft-Tissue Sample	Width W_0 [mm]	Thickness [mm]	Cross-sectional Area [mm^2]	Gauge Length L_1 [mm]	Aspect Ratio $L_1 : W_0$	Clamping Modifications
Hoffman (Hoffman et al., 1977)	1977	Aorta (thoracic, descending)	1.5					
Banks (Banks et al., 1978)	1978	Pulmonary artery (thorax)<br>Pulmonary Vein (thorax)	10					
Andreassen (Andreassen et al., 1980)	1980	Cornea (eye; normal and keratoconus)	2.4	0.52	1.25	3.5	1.46	
van Noort (van Noort et al., 1981)	1981	Dura mater (cranial)	5			40	8	Paper
Daly (Daly, 1982)	1982	Skin (abdomen)	1					
Akizuki (Akizuki et al., 1986)	1986	Articular cartilage (knee; normal, fibrillated, osteoarthritic)	1.8	0.25	0.45			
Akizuki (Akizuki et al., 1987)	1987	Articular cartilage (knee; normal, fibrillated, osteoarthritic)	1.8	0.25	0.45			
Sherebrin (Sherebrin et al., 1989)	1989	Aorta (thoracic; longitudinal and circumferential)	5					Sandpaper
Steiger (Steiger et al., 1989)	1989	Artery (intracranial; cerebral; aneurysm)	2 to 5					
Yoganandan (Yoganandan et al., 1989)	1989	Ligament (spinal, cervical; anterior longitudinal and flavum)						
Donovan (Donovan et al., 1990)	1990	Vein (leg; saphenous)	3.7 to 8.3	3.7 to 8.3	10.75 to 54.11			
Oxlund (Oxlund et al., 1990)	1990	Chorioamniotic membrane	4	0.044 to 0.243	0.176 to 0.972	7	1.75	Filter paper
van Alphen (van Alphen and Graebel, 1991)	1991	Lens, zonules, ciliary muscle, and choroid (eye)	5 (choroid)<br>5 (ciliary muscle)					
Rains	1992	Cartilage (neck; tracheal)	1	0.1	0.1	10 to 12	10 to 12	Glue

(Rains et al., 1992)								
Helmig (Helmig et al., 1993)	1993	Chorioamniotic membrane	4			7.4	1.85	Filter paper
He (He and Roach, 1994)	1994	Aorta (abdominal; normal, and aneurysm; longitudinal)	5					Sandpaper
Wolfinbarger (Wolfinbarger et al., 1994)	1994	Dura mater (cranial)	10	0.58	5.8	42	4.20	Sandpaper
Tissakht (Tissakht and Ahmed, 1995)	1995	Meniscus (knee; circumferential or circ. and radial or rad.)	1.75 to 3	1.5 to 2 (circ.)<br>0.8 to 2 (rad.)	2.625 (circ.)<br>0.875 (rad.)			
Pfaeffle (Pfaeffle et al., 1996)	1996	Interosseous membrane (forearm)	4.3	1.6	5.5			Epoxy putty, K-wires
Raghavan (Raghavan et al., 1996)	1996	Aorta (abdominal; aneurysm)	10					Cyanoacrylate glue
Har-Shai (Har-Shai et al., 1997)	1997	Fascia (face; superficial musculoaponeurotic system)	6 to 8	2 to 3				
Roberts (Roberts et al., 1997)	1997	Cartilage (neck; tracheal)	1	0.1	0.1	6 to 8	6 to 8	Cyanoacrylate glue
Afoke (Afoke et al., 1998)	1998	Fascia (hand; palmar; Dupuytren's contracture)		1.5				
Kim (Kim et al., 1998)	1998	Phayngeal tissue	12.79	3.15		40.2	3.14	
Sacks (Sacks et al., 1998)	1998	Dura mater (cranial; parallel and perpendicular fibers)	2	0.35 (parallel)<br>0.37 (perpend.)	0.7 (parallel)<br>0.4 (perpend.)			Cyanoacrylate glue, sandpaper
Tóth (Tóth et al., 1998)	1998	Artery (intracranial; cerebral; aneurysm)	1.5			3	2	
Davis (Davis and Wastell, 2000)	2000	Skin (scar; HIV and non-HIV)	1			5	5	Sutures
Elliott (Elliott and Setton, 2001)	2001	Annulus fibrosus (spine; lumbar; disc)	2.86	1.37	3.92			
Clavert (Clavert et al., 2001)	2001	Tendon (arm; biceps muscle, long head)			15.99	68.68		
Pennati (Pennati, 2001)	2001	Umbilical vein and Wharton’s jelly (umbilical cord)	4 (vein)<br>4 (Wharton’s)	1.25 (vein)		8 (vein)<br>8 (Wharton’s)	2 (vein)<br>2 (Wharton’s)	

Wang (Wang et al., 2001)	2001	Aorta (abdominal; aneurysm; intraluminal thrombus)	2	0.6				Cyanoacrylate glue
Egorov (Egorov et al., 2002)	2002	Esophagus (thorax)<br>Stomach (abdomen)<br>Small bowel (abdomen)<br>Large bowel (abdomen)	10 (esophagus)<br>10 (stomach)<br>10 (small bowel)<br>10 (large bowel)			25 (esophagus)<br>25 (stomach)<br>25 (small bowel)<br>25 (large bowel)	2.5 (esophagus)<br>2.5 (stomach)<br>2.5 (small bowel)<br>2.5 (large bowel)	
Schechtman (Schechtman and Bader, 2002)	2002	Tendon (foot; extensor digitorum longus muscle)			2.91	72		Sandblasted grips
Vanags (Vanags et al., 2003)	2003	Esophagus (thorax; wall; esophagitis)		4				
Vorp (Vorp et al., 2003)	2003	Aortic (thoracic, ascending; aneurysm)	3					Cyanoacrylate glue
Butler (Butler and Walsh, 2004)	2004	Ligament (ankle; anterior tibiotalar or ATTL, posterior tibiotalar or PTTL, tibiocalcaneal or TCL, anterior tibiofibular or ATiFL, posterior tibiofibular or PTiFL,anterior talofibular or ATFL, posterior talofibular or PTFL, and calcaneofibular or CFL)	16.7 (ATTL)<br>16.6 (PTTL)<br>19.7 (TCL)<br>16.3 (ATiFL)<br>18.3 (PTiFL)<br>11.1 (ATFL)<br>13.0 (PTFL)<br>10.5 (CFL)	2.5 (ATTL)<br>2.9 (PTTL)<br>2.8 (TCL)<br>1.8 (ATiFL)<br>2.7 (PTiFL)<br>1.8 (ATFL)<br>2.3 (PTFL)<br>1.5 (CFL)	41.75 (ATTL)<br>48.14 (PTTL)<br>55.16 (TCL)<br>29.34 (ATiFL)<br>49.41 (PTiFL)<br>ATFL 19.98<br>29.9 (PTFL)<br>15.75 (CFL)	5	0.30 (ATTL)<br>0.30 (PTTL)<br>0.25 (TCL)<br>0.31 (ATiFL)<br>0.27 (PTiFL)<br>0.45 (ATFL)<br>0.38 (PTFL)<br>0.48 (CFL)	Sandpaper
Holzapfel (Holzapfel et al., 2004)	2004	Atherosclerotic plaque (pelvis; iliac artery)	2.2 to 5.6	0.24 to 1.7				Sandpaper
Oyen (Oyen et al., 2004)	2004	Amniotic membrane (fetal)	6			11	1.83	Sandpaper
Stradins (Stradins et al., 2004)	2004	Aortic and pulmonary valves (thorax)	3	0.61 (aortic)<br>0.4 (pulmonary)	1.83 (aortic)<br>1.2 (pulmonary)			
Vogel (Vogel et al., 2004)	2004	Fetal membrane	4					
Grässel (Grässel et al., 2005)	2005	Fascia (abdominal; linea alba; supra- and infra-umbilical)	1					Sandpaper
Holzapfel (Holzapfel et al., 2005)	2005	Artery (heart; coronary, left anterior descending or LAD)	2.81	0.87		7.21	2.61 (coronary)<br>2.61 (LAD)	
Huang (Huang et al., 2005)	2005	Cartilage (shoulder; glenohumeral)	1.5	0.25	0.38	5	3.33	Sandpaper
Oyen (Oyen et al., 2005)	2005	Amniotic membrane (fetal)	5.5	0.05	0.28	11	2	Sandpaper

Richmon (Richmon et al., 2005)	2005	Aorta (abdominal; aneurysm)	8	1.57	12.56	7.3	0.91	
Defrate (Defrate et al., 2006)	2006	Tendon (foot; Achilles)	11		61 to 65.7			Beads, sandpaper
Di Martino (Di Martino et al., 2006)	2006	Aorta (abdominal; aneurysm; elective repair and ruptured)	2.5		2.5 (elective)<br>3.6 (ruptured)			
Lyu (Lyu et al., 2006)	2006	Plica (knee; mediopatellar)	2.1	1.5	3.15	10	4.76	Paper, adhesive
Raghavan (Raghavan et al., 2006)	2006	Aorta (abdominal; aneurysm)	8	1.48				
Seehra (Seehra and Silver, 2006)	2006	Skin	4	1	4	20	5	
Vande Geest (Vande Geest et al., 2006)	2006	Aorta (abdominal; aneurysm)	5					
Wagner (Wagner et al., 2006)	2006	Annulus fibrosus (spine; lumbar; anterior and contra-lateral)	5	2				Cyanoacrylate glue, sutures
Cheng (Cheng et al., 2007)	2007	Tympanic membrane (ear)	2	0.08				
Lujan (Lujan et al., 2007)	2007	Ligament (knee; medial collateral)	1.81	1.48	2.68	15.06	8.32	
Giannini (Giannini et al., 2008)	2008	Tendon (leg; posterior tibial muscle)			21.9 to 25.6			Cryoclamps
Kureshi (Kureshi et al., 2008)	2008	Fascia (abdominal; transversalis muscle)				5		
Rubod (Rubod et al., 2008)	2008	Vaginal wall (pelvis)	4	1.8		14	3.5	Hook-loop-tape
Smith (Smith et al., 2008)	2008	Labrum (shoulder; glenoid)	1	1	1	6	6	Cryoclamps
Xiong (Xiong et al., 2008)	2008	Aorta (abdominal; aneurysm and non-aneurysm; axial and circumferential)	5	1.5 to 1.9	7.5 to 9.5			Sandpaper
Birch (Birch and Srodon, 2009)	2009	Soft palate (mouth)	5.4	5.2	28.08			Acrylic blocks, cyanoacrylate Glue

Huang (Huang et al., 2009)	2009	Tendon (shoulder; supraspinatus muscle)	6.5	1.5				Sandpaper
Jabareen (Jabareen et al., 2009)	2009	Fetal membrane (amnion and chorion)	15 (amnion)<br>15 (chorion)	0.11 (amnion)<br>0.43 (chorion)	1.65 (amnion)<br>6.45 (chorion)	40	2.67 (amnion)<br>2.67 (chorion)	Sandpaper
Lake (Lake et al., 2009)	2009	Tendon (shoulder; supraspinatous muscle)	5	0.4	2.0			
O'Connell (O'Connell et al., 2009)	2009	Annulus fibrosus (spine; lumbar; circumferential and radial)	2.8 (circ.)<br>2.3 (rad.)	1.9 (circ.)<br>1.8 (rad.)				
Teng (Teng et al., 2009)	2009	Artery (neck; carotid)	2	1.24				Sandpaper, Cyanoacrylate glue,
Atienza (Atienza, 2010)	2010	Artery (heart; coronary; circumferential)	1	0.42 to 1.35	0.42 to 1.35			Cyanoacrylate glue
Claes (Claes et al., 2010)	2010	Artery (heart; coronary)	1	0.42 to 1.35	0.42 to 1.35			Cyanoacrylate glue
Duprey (Duprey et al., 2010)	2010	Aorta (thoracic, ascending; aneurysm)		1.86				Sandpaper
Elsheikh (Elsheikh et al., 2010)	2010	Sclera (eye)	4	1.06	4.24	12.1	3.03	
Hanuza (Hanuza et al., 2010)	2010	Aorta (thoracic)	10			30	3	
Komolafe (Komolafe and Doehring, 2010)	2010	Tendon (foot; Achilles)	4	1				
Lake (Lake et al., 2010)	2010	Tendon (shoulder; supraspinatus muscle)	5	0.5	2.5	13.9	2.78	
Martins (Martins et al., 2010)	2010	Vaginal wall (pelvis)		1.47				
Myers (Myers et al., 2010)	2010	Cervix (pelvis)		2		10		Sandpaper, cyanoacrylate glue
Abraham (Abraham et al., 2011)	2011	Meniscal attachments (knee)			3.7	6.1		
Costalat (Costalat et al., 2011)	2011	Artery (intracranial; aneurysm)		0.37	0.62			
Clay (Clay et al., 2011)	2011	Vaginal wall (pelvis; pelvic organ prolapse)	4			14	3.5	Hook-loop-tape

Gabriel (Gabriel et al., 2011)	2011	Vaginal tissue (pelvis)<br>Skin (abdomen; infraumbilical)<br>Aponeurosis (abdominal; infraumbilical)	4 (vaginal)<br>4 (skin)<br>4 (aponeurosis)	1 to 2	4 to 8	15 (vaginal)<br>15 (skin)<br>15 (aponeurosis)	3.75 (vaginal)<br>3.75 (skin)<br>3.75 (aponeurosis)	Hook-and-loop-tape
Khanafer (Khanafer et al., 2011)	2011	Aorta (thoracic, ascending; aneurysm)	3					Sandpaper
Kirlova (Kirilova et al., 2011)	2011	Fascia (abdominal; umbilical and inguinal; longitudinal and transverse)	10 (umbilical)<br>10 (inguinal)			30 (umbilical)<br>30 (inguinal)	3 (umbilical)<br>3 (inguinal)	
Martins (Martins et al., 2011b)	2011	Vaginal tissue (pelvis)						
Martins (Martins et al., 2011a)	2011	Bladder (pelvis)	10					
Pavan (Pavan et al., 2011)	2011	Aponeurosis (foot; plantar)	5	2.57	12.85	10	2	
Pena (Pena et al., 2011)	2011	Vaginal tissue (pelvis)	6	2.34	14.04	16	2.67	
Raghavan (Raghavan et al., 2011)	2011	Aorta (abdominal; aneurysm)	4	1.6				
Bourgouin (Bourgouin et al., 2012)	2012	Small intestine (abdomen)	25	1	25	32.7	1.31	
Celi (Celi et al., 2012)	2012	Aorta (abdominal; aneurysm, intraluminal and thrombus)	18					
Geraghty (Geraghty et al., 2012)	2012	Sclera (eye)	4	0.8	3.2	12.12	3.03	
Klinich (Klinich et al., 2012)	2012	Placenta tissue	25			60	2.4	Cryoclamps
Lake (Lake et al., 2012)	2012	Tendon (shoulder; supraspinatous muscle)	5	0.4	2.0			
Martins (Martins et al., 2012)	2012	Fascia (abdominal; rectus sheath)		1				Aluminum, sandpaper, hook-and-loop-tape
Rubod (Rubod et al., 2012)	2012	Vagina, bladder, and rectum (pelvis)	4	1.5	6	15	3.75	Hook-and-loop-tape
Teng (Teng et al., 2012)	2012	Muscle (neck; trachealis; mucosa, submucosa, and adventitia layers)						Sandpaper

Trindale (Trindade et al., 2012)	2012	Tendon and fascia (head; temporalis muscle)	2					
Dodson (Dodson et al., 2013)	2013	Umbilical artery (normal, preeclampsia; axial and circumferential)						
Khanafer (Khanafer et al., 2013)	2013	Aorta (thoracic, ascending; aneurysm)	3					
Kirlova (Kirilova et al., 2013)	2013	Fascia (abdominal; umbilical; longitudinal and transverse)	10					
Kobielarz (Kobielarz and Jankowski, 2013)	2013	Aorta (abdominal aneurysm)	5					
Li (Li et al., 2013)	2013	Aorta and artery (pulmonary)	6					Cryoclamps
Martins (Martins et al., 2013a)	2013	Vaginal						
Martins (Martins et al., 2013b)	2013	Ligament (pelvis; uterosacral)	10					
Pichamuthu (Pichamuthu et al., 2013)	2013	Aorta (thoracic, ascending; bicuspid and tricuspid aortic valves)	10			40	4	Sandpaper, cyanoacrylate glue
Reeps (Reeps et al., 2013)	2013	Aorta (abdominal; aneurysm)	5	1.57	7.85	7.3	1.46	
Rivaux (Rivaux et al., 2013)	2013	Ligament (pelvis; uterosacral or USL, round or RL, and broad or BL)	4 (USL)<br>4 (RL)<br>4 (BL)			14 (USL)<br>14 (RL)<br>14 (BL)	3.5 (USL)<br>3.5 (RL)<br>3.5 (BL)	
Trindale (Trindade et al., 2013)	2013	Muscle (head; temporalis)	4					
Faturechi (Faturechi et al., 2014)	2014	Fetal membrane (amnion)	8	0.05				
Isaacs (Isaacs et al., 2014)	2014	Annulus fibrosus (spine; thoracic and lumbar)	2	0.16	0.32	5	2.50	Sandpaper cyanoacrylate glue
Karimi (Karimi et al., 2014)	2014	Artery and vein (umbilical)	2					
Stecco (Stecco et al., 2014)	2014	Fascia (hip/thigh; crural; anterior and posterior)	3			10	3.3	Hook-and-loop tape

Tavares (Tavares Monteiro et al., 2014)	2014	Aorta (abdominal; aneurysm)	5					
Teng (Teng et al., 2014)	2014	Artherosclerotic plaque (neck; carotid, external; media, fibrous cap, lipid, and intraplaque hemorrhage/thrombus or IPH/T)	1.85 (media)<br>0.97(fibrous cap)<br>1.74 (lipid)<br>1.62 (IPH/T)	0.91 (media)<br>0.26 (fibrous cap)<br>1.28 (lipid)<br>1.31 (IPH/T)	1.68 (media)<br>0.25 (fibrous cap)<br>2.23 (lipid)<br>2.12 (IPH/T)			
Voycheck (Voycheck et al., 2014)	2014	Joint capsule (shoulder; glenohumeral)	10	0.4	0.4		2.4	
Chebil (Chebil et al., 2015)	2015	Ligament (intraabdominal; gastrocolic)	25	4.30	107.5	107	4.28	Sandpaper
Faturechi (Faturechi et al., 2015)	2015	Chorioamniotic membrane	8	0.05	0.4			Sandpaper
Ferro (Ferro et al., 2015)	2015	Acetabular labrum (hip)			31.45	30		Wire-wrapped ends, sandpaper
Jankowska (Jankowska et al., 2015)	2015	Artery (heart; coronary; stages of atherosclerosis development or SAD)	5 (SAD I)<br>5 (SAD II)<br>5 (SAD III)	1.95 (SAD I)<br>0.98 (SAD II)<br>2.5 (SAD III)	3.9 (SAD I)<br>1.96 (SAD II)<br>5 (SAD III)	25 (SAD I)<br>25 (SAD II)<br>25 (SAD III)	5 (SAD I)<br>5 (SAD II)<br>5 (SAD III)	Texturized rubber
Kobielarz (Kobielarz et al., 2015)	2015	Aorta (thoracic; atherosclerotic	5					
Lopez (Lopez et al., 2015)	2015	Vaginal tissue (pelvis; anterior wall)						
Mauri (Mauri et al., 2015)	2015	Fetal membrane (amnion)	15			60	4	
Martufi (Martufi et al., 2015)	2015	Aorta (abdominal; wall or W and thrombus or T)	4	1.83 to 3.6 (W)<br>17 to 32 (T)	7.32 to 1.8 (W)<br>68 to 128 (T)			
Ninomiya (Ninomiya et al., 2015)	2015	Aorta (thoracic and abdominal)	4	1.3 (thoracic)<br>1.16 (abdominal)				
O’Leary (O'Leary et al., 2015)	2015	Aorta (abdominal; aneurysm)	4	1.24	4.96	16	4	
Pierce (Pierce et al., 2015)	2015	Aorta (thoracic and abdominal; aneurysm; longitudinal and circumferential)	4	1.98 (thoracic)<br>2.12 (abdominal)	7.92 (thoracic) to 8.48 (abdominal)			Sandpaper, glue
Skelley (Skelley et al.,	2015	Ligament (knee; anterior cruciate)		0.56		11.6		Sandpaper, cyanoacrylate glue

2015)								
Sun (Sun et al., 2015)	2015	Eyelid (eye; tarsus)	5.51	1.60				
Tanios (Tanios et al., 2015)	2015	Aorta (abdominal; aneurysm)	8					
Teng (Teng et al., 2015)	2015	Aorta (abdominal; aneurysm)	1.99	1.24				
Yamada (Yamada et al., 2015)	2015	Aorta (ascending; dissected)	3 (dissected)<br>3 (control)	1.66 (dissected)<br>1.46 (control	4.98 (dissected)<br>4.38 (control)	10	3.33 (dissected)<br>3.33 (control)	
Aisa (Aisa et al., 2016)	2016	Vein (leg; saphenous)						
Barrett (Barrett et al., 2016)	2016	Plaque tissue (neck; carotid)	25.02			5.16	0.20	
Brieu (Brieu et al., 2016)	2016	Vaginal tissue (pelvis; anterior and posterior wall)	4	1.80				
Cooney (Cooney et al., 2016)	2016	Fascia (abdominal; linea alba; transverse and longitudinal)	10.74 (tranverse)<br>11.94 (longitudinal)	1.37 (transverse)<br>1.27 (longitudinal)	14.71 (transverse)<br>15.16 (longitudinal)	20.57 (transverse)<br>25.43 (longitudinal)	1.92 (transverse)<br>2.13 (longitudinal)	Sandpaper
Criscenti (Criscenti et al., 2016)	2016	Tendon (knee; patello-femoral)	8.9 to 25.9	0.9 to 2.1	8.7 to 55.3		4	Sandpaper, cyanoacrylate glue
de Landsheere (de Landsheere et al., 2016)	2016	Vaginal tissue (pelvis)	4					
Gaur (Gaur et al., 2016)	2016	Diaphragm	19	2.25	34.34	34	1.79	Cryoclamps
Kirlova- Doneva (Kirilova-Doneva et al., 2016)	2016	Fascia (abdominal; umbilical)	10					
Liu (Liu et al., 2016)	2016	Skin (breast; stratum corneum)	7			15	2.14	Tape
Massalou (Massalou et al., 2016)	2016	Colon (abdomen)	25			40	1.60	
Morales-Orcajo (Morales-Orcajo et al., 2016)	2016	Tendon (foot; extensor digitorum brevis or EDB and longus or EDL; flexor digitorum brevis or FDB and longus or FDL; extensor hallucis longus or EHL; flexor hallucis longus or FHL; tibialis anterior or TA and			3.09 (EDB)<br>4.81 (EDL)<br>2.72 (FDB)<br>4.87 (FDL)<br>8.53 (EHL)<br>15.77 (FHL)			Steel clamps with milled holes

		posterior or TP; and peroneus brevis or PB and longus or PL muscles)			26.40 (TA)<br>24.02 (TP)<br>11.87 (PB)<br>16.59 (PL)			
Schleifenbaum (Schleifenbaum et al., 2016a)	2016	Ligaments (hip; ilio-, pubo-, and ischio-femoral)						Plastinated ends
Skelley (Skelley et al., 2016)	2016	Ligament (knee; anterior cruciate)		0.60				
Wright (Wright et al., 2016b)	2016	Ligament (knee; posterior cruciate)						Sandpaper
Wright (Wright et al., 2016a)	2016	Disc (jaw; temporomandibular joint; anteroposterior or AP and mediolateral or ML)	2 (AP)<br>2 (AL)	2.2 (AP)<br>2.5 (AL)	4.4 (AP)<br>5.0 (AL)	7.1 (AP)<br>10.6 (ML)	3.55 (AP)<br>5.3 (ML)	
Yan (Yan et al., 2016)	2016	Meniscus (knee)						Collets
Antun (Antun et al., 2017)	2017	Fetal membrane	10					Filter paper
Brunenieks (Brunenieks et al., 2017)	2017	Colon (pelvis; rectum; normal and obstructed defecation syndrome or ODS)	5	2.89 (normal)<br>2.71 (normal)<br>2.28 (ODS, ant.)<br>2.12 (ODS, post.)	14.45 (normal)<br>13.55 (normal)<br>11.4 (ODS, ant.)<br>10.6 (ODS, post.)			
Kumaraswamy (Kumaraswamy et al., 2017)	2017	Skin (breast)	11.97	1.	18.07	43	3.59	Sandpaper
Ollivier (Ollivier et al., 2017)	2017	Aorta (abdominal; aneurysm; fibrous and calcified)	4	1.2		16	4	
Astruc (Astruc et al., 2018)	2018	Rectus sheath (abdominal; anterior and posterior sheath, linea alba)	10 (ARS)<br>10 (PRS)<br>10 (LA)	0.73 (ARS)<br>0.58 (PRS)<br>1.97 (LA)	7.3 (ARS)<br>5.8 (PRS)<br>19.7 (LA)	15 (ARS)<br>15 (PRS)<br>15 (LA)	1.5 (ARS)<br>1.5 (PRS)<br>1.5 (LA)	Grips with stiches and grids
Bai (Bai et al., 2018)	2018	Aorta (thoracic, ascending)	5					
Brunel (Brunel et al., 2018)	2018	Artery (intracranial; cerebral; aneurysm)						
Deveja (Deveja et al., 2018)	2018	Aorta (thoracic; ascending; bicuspid and tricuspid aortic valves)	10					Sandpaper, cyanoacrylate glue
Fatemifar (Fatemifar et al., 2018)	2018	Trabeculae carneae (heart; muscle)	2.06	1.11	2.29			Suture hooks

Guo (Guo et al., 2018)	2018	Fascia (foot; plantar)	2	0.65				Zig-zag grip
Karimi (Karimi and Razaghi, 2018)	2018	Lung (thorax)	15.1	6.46				
Karimi (Karimi and Shojaei, 2018)	2018	Liver (abdomen)	24.35	9.93				Sandpaper, cyanoacrylate glue
Leng (Leng et al., 2018)	2018	Artery (neck; carotid; atherosclerotic media and fibrous cap						
Otsuka (Otsuka et al., 2018)	2018	Fascia lata (hip; longitudinal and transverse)	40	0.19 to 0.8	7.6 to 32.0			Sandpaper, glue
Pham (Pham et al., 2018)	2018	Annulus fibrous (spine; lumbar)	1.75	0.5	0.87	4.37	2.49	Cotton fibre tabs, cyanoacrylate Glue
Wagner (Wagner et al., 2018)	2018	Fetal membrane (amnion)	4					
Xiang (Xiang et al., 2018)	2018	Corneal stroma (eye)	1.2			1.5	1.25	
Xue (Xue et al., 2018)	2018	Corneal stroma (eye)	1	0.1	0.1	1.5	1.5	Roughened-surface grips
Amabili (Amabili et al., 2019)	2019	Aorta (thoracic, descending; intima, media and adventitia layers)	8	0.15 (intima)<br>1.02 (media)<br>0.59 (adventitia)				
Khanafer (Khanafer et al., 2019)	2019	Aorta (thoracic, ascending)	3					
Kochova (Kochova et al., 2019)	2019	Perineal body (pelvis)	14	7	98	10	0.71	
Koh (Koh et al., 2019)	2019	Fetal membrane (amnion)	5					Sandpaper, cyanoacrylate glue
Kozun (Kozun et al., 2019)	2019	Aorta (thoracic, ascending; aneurysm and normal)	5			20	4	
Massalou (Massalou et al., 2019a)	2019	Large intestine (abdomen)	25			40	1.6	
Massalou (Massalou et al., 2019b)	2019	Large intestine (abdomen; ascending, transverse, descending and sigmoid)	25 (ascending)<br>25 (transverse)<br>25 (descending)<br>25 (sigmoid)	1.2 (ascending)<br>1.2 (transverse)<br>1.4 (descending)<br>1.6 (sigmoid)	30 (ascending)<br>30 (transverse)<br>35 (descending)<br>37.6 (sigmoid)	40 (ascending)<br>40 (transverse)<br>40 (descending)<br>40 (sigmoid)	1.6 (ascending)<br>1.6 (transverse)<br>1.6 (descending)<br>1.6 (sigmoid)	
Rotta	2019	Stomach (abdomen)	10 to 30	3.57 to 4.25	35.7 to 127.5			

(Rotta et al., 2019)								
Shan (Shan et al., 2019)	2019	Aponeurosis (leg; triceps surae muscles)						Sandpaper
Sokolis (Sokolis, 2019)	2019	Ureter (abdomen)	3			15	5	Sandpaper
Amabili (Amabili et al., 2020)	2020	Aorta (thoracic, descending; dissected; intima, media and adventitia layers; axial and circumferential)	8	0.2 (intima)<br>0.98 (media)<br>1.9 (adventitia)	1.6 (intima)<br>7.84 (media)<br>15.2 (adventitia)			
Bhat (Bhat et al., 2020)	2020	Artery (neck; carotid; intima layer)	1.5	1	1.5			
Carniel (Carniel et al., 2020)	2020	Stomach (abdomen)	9.93	4.51	44.78	15	1.51	Glue, balsa wood
Fischer (Fischer et al., 2020)	2020	Iliotibial tract (thigh)	11.3	0.9	1.02	50	4.42	Plastination, wooden taps
Gaur (Gaur et al., 2020)	2020	Diaphragm		2.25			1.9	Cryoclamps
Gawargious (Gawargious et al., 2020)	2020	Sclera (eye)	2			5	2.5	
Gomes (Gomes et al., 2020)	2020	Perivascular tissue (periaortic aneurysm)	10	4	40	40	4	
Kriilova-Doneva (Kirilova-Doneva et al., 2020)	2020	Fascia (abdominal; umbilical, inguinal and abdominal; different ages)	10 (<60 years)<br>10 (61-80 years),<br>10 (>80 years)	0.71 (<60 years)<br>1.61 (61-80 years), 1.45 (>80 years)	7.1 (<60 years)<br>16.1 (61-80 years), 14.5 (>80 years)	30	3	Roughened rubber
Kobielarz (Kobielarz, 2020)	2020	Aorta (abdominal; normal and aneurysm)	5			25	5	
Liu (Liu et al., 2020b)	2020	Corneal stroma (eye)		0.13				
Liu (Liu et al., 2020a)	2020	Cornea (eye; lenticule, nasal-tempora or NT and superior-inferior or SI)	1.2 (NT)<br>1.2 (SI)			1.5 (NT)<br>1.5 (SI)	1.25 (NT)<br>1.25 (SI)	
Nguyen (Nguyen et al., 2020)	2020	Sclera (eye)						
Novotny (Novotny et al., 2020)	2020	Artery (pelvis; iliac)	3					
Paritala (Paritala et al., 2020)	2020	Carotid plaque (neck; artery).	3.14	1.17	3.21	5	1.59	Sandpaper, cyanoacrylate glue

Shin<br>(Shin et al., 2020)	2020	Optic nerve sheath (eye; inner and/or outer layers)	2 (inner)<br>2 (outer)<br>2 (both)		0.67 (inner)<br>0.49 (outer)<br>0.92 (both)	4 (inner)<br>4 (outer)<br>4 (both)	2 (inner)<br>2 (outer)<br>2 (both)	Cyanoacrylate glue, cardboard, serrated grips
Franchini<br>(Franchini et al., 2021)	2021	Aorta (thoracic, descending; intima, media and adventitia layers; circumferential and longitudinal)	5	1.67	8.35			
Gomes<br>(Gomes et al., 2021)	2021	Perivascular tissue (periaortic, aneurysm)	10	4	40	40	4	
He<br>(He et al., 2021b)	2021	Retina, choroid and sclera (eye; age-related macular degeneration)	1.5 (retina)<br>1.5 (choroid)<br>0.5 (sclera)			1 (retina)<br>1 (choroid)<br>1 (sclera)	0.67 (retina)<br>0.67 (choroid)<br>2 (sclera)	
Kozun<br>(Kozun et al., 2021)	2021	Aorta (thoracic; atherosclerosis)	5	1.1	5.5	25	5	
Park<br>(Park et al., 2021)	2021	Sclera (eye; anterior, equatorial, posterior, peripapillary)<br>Optic nerve sheath (eye)	0.6 (anterior)<br>0.6 (equatorial)<br>0.7 (posterior)<br>0.8 (peripapillary)<br>1.3 (nerve sheath)				4.9 (anterior)<br>4.7 (equatorial)<br>4.6 (posterior)<br>3.5 (peripapillary)<br>5.6 (nerve sheath)	
Todros<br>(Todros et al., 2021)	2021	Plantar fascia (foot)	5			15	3	
Xue<br>(Xue et al., 2021)	2021	Cornea (eye)	1	0.111	0.11	1.5	1.5	Roughen thread surface
Baldit<br>(Baldit et al., 2022)	2022	Umbilical cord<br>(Wharton's Jelly)	4.69	1.01	4.74	11.98	11.86	Cyanoacrylate glue
Chang<br>(Chang et al., 2022)	2022	Meniscus (knee)	4	1	4	5 to 7	1.25 to 1.75	Aluminum beads, cyanoacrylate glue
Durcan<br>(Durcan et al., 2022)	2022	Esophagus (thorax; muscularis propria and mucosa, and submucosa; axial and circumferential)	4.1				4<br>ASTM E8/E8M-13	
Kirlova-Doneva<br>(Kirilova-Doneva and Pashkouleva, 2022)	2022	Fascia (abdominal; umbilical and transversalis)	10 (umbilical)<br>10 (transversalis)			40 (umbilical)<br>40 (transversalis)	4 (umbilical)<br>4 (transversalis)	
Ma<br>(Ma et al., 2022)	2022	Sclera (eye)<br>Dura matter (cranial)	3					
Metschl<br>(Metschl et al., 2022)	2022	Aorta (abdominal; aneurysm)	8			7.3	0.91	
Song<br>(Song et al., 2022)	2022	Stroma (eye; corneal)	1					
Tokgoz<br>(Tokgoz et al.,	2022	Aorta (thoracic, ascending and descending; aneurysm; intima, media	1.5	0.99 (intima)<br>1.67 (media)	1.49 (intima)<br>2.51 (media)			

2022)		and adventitia layers; axial and circumferential)		0.85 (adventitia)	1.275 (adventitia)			

Supplementary Table 4

Included studies using a tapered sample shape, arranged by publication year and last name of the first author. All retrieved measurements are from the gauge section of the sample. Aspect ratios and clamping modifications are highlighted (light and dark grey respectively).

First Author, Last name	Year	Human Soft-Tissue Sample	Width W_0 [mm]	Thickness [mm]	Cross-sectional Area [mm^2]	Gauge Length L_1 [mm]	Aspect Ratio $L_1 : W_0$	Clamping Modifications
Kempson (Kempson et al., 1968)	1968	Cartilage (knee; femoral)	1.6			6.35	3.98	
Kempson (Kempson et al., 1973)	1973	Cartilage (knee; femoral)	1.6			6.35	3.98	
Weightman (Weightman, 1976)	1976	Cartilage (knee; femoral)		0.2				
Weightman (Weightman et al., 1978)	1978	Cartilage (knee; femoral)		0.2				
Mohan (Mohan and Melvin, 1982)	1982	Aorta (thoracic; longitudinal and transverse)	6.36<br>4.57			19.05<br>7.87	3.00<br>1.72	
Kempson (Kempson, 1991)	1991	Cartilage (knee; femoral)<br>Cartilage (foot; talar)	10 (knee)<br>10 (foot)	0.2 (knee)<br>0.2 (foot)	2 (knee)<br>2 (foot)	30 (knee)<br>30 (foot)	3 (knee)<br>3 (foot)	
Loree (Loree et al., 1994)	1994	Aorta (abdominal; atherosclerotic plaque)	4.8	1.2		7.9	1.64	Sandblasted clamps
Skaggs (Skaggs et al., 1994)	1994	Annulus fibrosis (spine; lumbar)	1.51	0.63		2.9	1.92	
Ebara (Ebara et al., 1996)	1996	Anulus fibrosus (spine; lumbar)	4.4	2.3	10.12	3.7	0.84	Polishing paper
Di Martino (Di Martino et al., 1998)	1998	Aorta (abdominal; thrombus)	4		14.5	22	5.5	Ribbed clamps
Quapp (Quapp and Weiss, 1998)	1998	Ligament (knee; medial collateral)	1.75 (longitudinal)<br>3.56 (transverse)	1.57 (longitudinal)<br>1.29 (transverse)	2.75 (longitudinal)<br>4.59 (transverse)	10 (longitudinal)<br>6 (transverse)	5.71 (longitudinal)<br>1.69 (transverse)	Gauze-wrapped ends
Lechner (Lechner et al., 2000)	2000	Meniscus (knee; medial)	1	0.5 to 3.0	0.5 to 3.0	10	10	Sandpaper, cyanoacrylate glue
Bellucci (Bellucci and Seedhom, 2001)	2001	Cartilage (knee, femoral and tibial)	1	0.2				Plastic blocks, Cyanoacrylate Glue

Moore(Moore et al., 2004)	2004	Joint capsule (shoulder; glenohumeral; posterior; longitudinal and transverse)	2.5 (longitudinal)<br>2.5 (transverse)		5.7 (longitudinal)<br>4.8 (transverse)	12.5 (longitudinal)<br>12.5 (transverse)	5 (longitundinal)<br>5 (transverse)	
Carpenter (Carpenter et al., 2005)	2005	Tendon (arm; biceps, long head; full-thickness tear and no tear)	3		20 (no tear)<br>15 (tear)			Cryoclamps (frozen normal saline block)
Moore (Moore et al., 2005)	2005	Joint capsule (shoulder; glenohumeral, posterior; longitudinal and transverse)	2.5 (longitudinal)<br>2.5 (transverse)		4.0 (longitudinal)<br>3.1 (transverse)	12.5 (longitudinal)<br>12.5 (transverse)	5 (longitundinal)<br>5 (transverse)	
Schmid (Schmid et al., 2005)	2005	Aorta (axial and circumferential)						Sandpaper
Stemper (Stemper et al., 2005)	2005	Artery (neck; internal carotid)	7.9			15.9	2.01	Wire-mesh
Ticker (Ticker et al., 2006)	2006	Ligament (shoulder; inferior glenohumeral)		1				
Temple (Temple et al., 2007)	2007	Cartilage (knee; femoral)	0.8			4	5	
Bae (Bae et al., 2008)	2008	Cartilage (knee; patellar)	0.8	0.3	0.24	4	5	
Craiem (Craiem et al., 2008)	2008	Aorta (thoracic, ascending)	2	2.00 to 2.25				Cyanoacrylate glue
Manoogian (Manoogian et al., 2008)	2008	Chorion and placenta	7	5	42.2 (chorion)<br>27.1 (placenta)	17	2.43 (chorion)<br>2.43 (placenta)	Serrated grip
Walraevens (Walraevens et al., 2008)	2008	Aorta (abdominal; calcified and normal)	4 (calcified)<br>4 (normal)				5 (calcified)<br>5 (normal)	
Iliopoulos (Iliopoulos et al., 2009b)	2009	Aorta (thoracic, ascending; aneurysm and control)	2 (control)<br>2 (aneurysm)	1.73 (control)<br>1.63 (aneurysm)		25 (control)<br>25 (aneurysm)	12.5 (control)<br>12.5 (aneurysm)	Sandpaper
Iliopoulos (Iliopoulos et al., 2009a)	2009	Aorta (thoracic, ascending; aneurysm)	2	1.71		25	12.5	Sandpaper
Maher (Maher et al., 2009)	2009	Atherosclerotic plaques (neck; carotid)	1			4	4	Sandpaper
Manoogian (Manoogian et al., 2009)	2009	Placenta	7		28.4	17	2.43	Sandpaper
Temple-Wong (Temple-Wong et al., 2009)	2009	Cartilage (knee; femoral, lateral condyle)	1.6			6.35	3.98	

Abramowitch (Abramowitch et al., 2010)	2010	Tendon (leg; semitendinosus and gracilis muscles)	3		15.53	30	10	Gauze
Chauvet (Chauvet et al., 2010)	2010	Dura mater (cranial)		1.11				
Guinea (Guinea et al., 2010)	2010	Aorta (thoracic, descending; longitudinal and circumferential)	2	1.9 to 2.1		10	5	Cyanoacrylate glue
Fessel (Fessel et al., 2011)	2011	Tendon (arm; flexor digitorum profundus muscle)						Ends wrapped in cloth
Garcia-Herrera (Garcia-Herrera et al., 2012a)	2012	Aorta (thoracic, ascending; newborn and young adult)	2 (newborn)<br>2 (young adult)	1.5 to 2.4	3.0 to 4.8	10 (newborn)<br>10 (young adult)	5 (newborn)<br>5 (young adult)	
Garcia-Herrera (Garcia-Herrera et al., 2012b)	2012	Aorta (thoracic, descending)	2	1.8	3.6	10	5	
Ni Annaidh (Ní Annaidh et al., 2012)	2012	Skin (back)	6	2.56	15.36	33	5.5	
Weisbecker (Weisbecker et al., 2012)	2012	Aorta (thoracic or T and abdominal or A; intima, media, adventitia layers; circumferential and axial)	4 (intima, T)<br>4 (media, T)<br>4 (adventia, T)<br>4 (intima, A)<br>4 (intima, A)<br>4 (intima, A)	0.48 (intima, T)<br>1.18 (media, T)<br>0.93 (adventia, T)<br>0.68 (intima, A)<br>0.94 (intima, A)<br>1.07 (intima, A)		10 (intima, T)<br>10 (media, T)<br>10 (adventia, T)<br>10 (intima, A)<br>10 (intima, A)<br>10 (intima, A)	2.5 (intima, T)<br>2.5 (media, T)<br>2.5 (adventia, T)<br>2.5 (intima, A)<br>2.5 (intima, A)<br>2.5 (intima, A)	
Iliopoulos (Iliopoulos et al., 2013)	2013	Coronary sinus	2	1.57	3.14	7	3.5	
Ben Abdelounis (Ben Abdelounis et al., 2013)	2013	Rectus sheath (abdomen; anterior)	5	1.2	6	20	4	Sandpaper
Forsell (Forsell et al., 2013)	2013	Aorta (abdominal; aneurysm)	4	15.7				Sandpaper, cyanoacrylate glue
Weisbecker (Weisbecker et al., 2013)	2013	Aorta (thoracic; intima, media, adventia layers; circumferential and axial)	4			10	2.5	
Fields (Fields et al., 2014)	2014	Cartilage (spine; lumbar; endplate)	1.5			5	3.33	
Hammer (Hammer et al., 2014)	2014	Fascia (leg; iliotibial tract)	10					Plastinated ends
Kritharis (Kritharis et al.,	2014	Aorta (ascending; sinus; circumferential or circ. and	2			7	3.5 (circ.)<br>3.5 (long.)	

2014)		longitudinal or long.)							
Nagle (Nagle et al., 2014)	2014	Muscle (pelvis; levator ani)	3	7.55	22.65	39.58	13.19		
Qian (Qian et al., 2014)	2014	Aponeurosis (hand; finger extensor)	2	1.07 to 2.23		10	5	Gauze	
Fischenich (Fischenich et al., 2015)	2015	Meniscus (knee)	1	1	1	5	5	Polishing paper, cyanoacrylate glue	
Freutel (Freutel et al., 2015)	2015	Meniscus (knee)	2.1	1				Sandpaper, cyanoacrylate glue	
Kolz (Kolz et al., 2015)	2015	Tendon (arm; biceps muscle, long head)							
Ottenio (Ottenio et al., 2015)	2015	Skin	5.2 (longitudinal) 3.55 transverse)	2.30	11.96 (longitudinal) 8.17 (transverse)	71 (longitudinal) 12.5 (transverse)	13.65 (longitudinal) 3.52 (transverse)		
Sassani (Sassani et al., 2015)	2015	Aorta (abdominal; aneurysm)	4	2.90	11.6	12	3	Sandpaper	
Schriefl (Schriefl et al., 2015)	2015	Aorta (abdominal)				4			
Utarouiu (Untaroiu et al., 2015)	2015	Liver parenchyma (abdomen)	10	5.00	50	55.5	5.55		
Courtail (Courtial et al., 2016)	2016	Aorta (abdominal)	4			17	4.25		
Ferrara (Ferrara et al., 2016)	2016	Aorta (thoracic, ascending; dilated)						Sandpaper, cyanoacrylate glue	
Schleifenbaum (Schleifenbaum et al., 2016b)	2016	Muscle (thigh; quadriceps femoris)	>14					Suture, plastinated ends	
Smeets (Smeets et al., 2017b)	2017	Ligaments (knee)						Sandpaper, cyanoacrylate glue	
Smeets (Smeets et al., 2017c)	2017	Ligament (knee; anterolateral) Ligament (shoulder; glenohumeral)						Cyanoacrylate glue	
Smeets (Smeets et al., 2017a)	2017	Tendon (leg; gracilis, semitendinosus and quadriceps muscles) Fascia (leg; iliotibial tract)						Sandpaper, cyanoacrylate glue	
Smoljkic (Smoljkic et al., 2017)	2017	Aorta (thoracic, ascending; aneurysm)	2	2.72	5.44			Sandpaper	

Tenorio (Tenorio et al., 2017)	2017	Parietal pleura (thorax)	2					
Falland-Cheung (Falland-Cheung et al., 2018)	2018	Scalp (head)	4			16	4<br>ISO 527-2	
Ferrara (Ferrara et al., 2018)	2018	Aorta (thoracic, ascending; dilated)						
Manopoulos (Manopoulos et al., 2018)	2018	Aorta (thoracic, ascending; inner and outer layers; circumferential or circ. and longitudinal or long.)	3 (circ., in.)<br>3 (circ., out.)<br>3 (long., in.)<br>3 (long., out.)	1.89 (circ., in.)<br>0.25 (circ., out.)<br>1.28 (long., in.)<br>0.26 (long., out.)	5.67 (circ., in.)<br>0.75 (circ., out.)<br>3.84 (long., in.)<br>10.26 (long., out.)	8 (circ., in.)<br>8 (circ., out.)<br>8 (long., in.)<br>8 (long., out.)	2.67 (circ., in.)<br>2.67 (circ., out.)<br>2.67 (long., in.)<br>2.67 (long., out.)	Sandpaper, cyanoacrylate glue
Scholze (Scholze et al., 2018)	2018	Scalp (head)<br>Fascia (leg; iliotibial band)<br>Tendon (forearm; flexor digitorum superficialis and profundus muscles)						3D-printed pyramids
Aydin (Aydin et al., 2019)	2019	Dura mater (cranial)	6	0.48 to 0.65		25	4.17<br>JISK6251-5	
Gremare (Gremare et al., 2019)	2019	Fetal membrane (amnion)	2.5	0.0045	0.01125	6	2.40	
Kizmazoglu (Kizmazoglu et al., 2019)	2019	Dura mater (cranial)	5			20	4<br>JISK6251-5-2004	
Lozano (Lozano et al., 2019)	2019	Fascia (iliotibial tract)						3D printed clamps
Sherifova (Sherifova et al., 2019)	2019	Aorta (thoracic, descending; aneurysmal and non-aneurysmal)	4			5	1.25 (aneurysm)<br>1.25 (non-aneurysm)	
Zwirner (Zwirner et al., 2019a)	2019	Fascia (leg; iliotibial tract)	12					Plastinated ends
Zwirner (Zwirner et al., 2019b)	2019	Dura mater (cranial)	5		2.84	10	2	3D-printed pyramids
Choi (Choi et al., 2020)	2020	Mucosa (mouth; buccal, hard palate, gingiva)	5	2	10	58	11.6<br>ISO 527-2	
Zwirner (Zwirner et al., 2020a)	2020	Muscle (head; temporalis)	5			10	2	3D-printed pyramids
Zwirner (Zwirner et al., 2020c)	2020	Fascia (head; temporalis muscle)	5		4	10	2	3D-printed pyramids
Zwirner	2020	Fascia (leg; iliotibial tract)					5	

(Zwirner et al., 2020b)							ISO 527-1	
Zwirner (Zwirner et al., 2019b; Zwirner et al., 2020d)	2020	Dura mater (cranial)	5		2.84	10	2	3D-printed pyramids
Grgic (Grgić et al., 2021)	2021	Tendon (leg; gracillus and quadricep muscles)	9.12 (gracillus)<br>9.91 (quadriceps)	2.73 (gracillus)<br>2.46 (quadriceps)	10.65 (gracillus)<br>19.36 (quadriceps)	25<br>12	6.25 (gracillus)<br>6 (quadricep)<br>ISO 527-1:1993	
He (He et al., 2021a)	2021	Aorta (thoracic, ascending; aneurysm; circumferential and longitudinal)					4	Sandpaper, cyanoacrylate glue
Nesbitt (Nesbitt et al., 2021)	2021	Meniscus (knee; lateral; younger or Y versus older or O; longitudinal and transverse)	1.0 (longitudinal)<br>1.3 (transverse)		0.79 and 0.76 (Y and O, longitudinal)<br>1.15 and 1.18 (Y and O, transverse)	5 (longitudinal)<br>3 (transverse)	5 (longitudinal)<br>2.31 (transverse)	
Polzer (Polzer et al., 2021)	2021	Aorta (abdominal; aneurysm)	2	1.48	2.96	4	2	
Wertheimer (Wertheimer et al., 2021)	2021	Tendon (leg; gracillus and quadriceps muscles)	5.19 (gracillus)<br>9.61 (quadriceps)	2.64 (gracillus)<br>2.45 (quadriceps)	10.51 (gracillus)<br>19.28 (quadriceps)		5 (gracilus)<br>5 (quadriceps)<br>ISO 527-1:1993 5A and 5B	
Zwirner (Zwirner et al., 2021b)	2021	Fascia (face; temporal muscle orTMF)<br>Dura mater (cranial)	5	1.46 (TMF)<br>0.63 (dura)	7.3 (TMF)<br>3.15 (dura)	10	2	3D-printed pyramids
Henderson (Henderson et al., 2022)	2022	Meniscus (knee; medial)	1	0.9	0.9	5	5	Cloth, cyanoacrylate glue
Zwirner (Zwirner et al., 2022)	2022	Scalp (head, temporal cutis and subcutis)<br>Fascia (head; temporalis muscle)<br>Muscle (head; temporalis)<br>Dura mater (cranial; temporal)					5 (scalp)<br>5 (fascia)<br>5 (muscle)<br>5 (dura mater)<br>ISO 527-2	

Supplementary Table 5

Included studies using both a non-tapered and tapered sample shape, arranged by publication year and last name of the first author. All retrieved measurements are from the gauge section of the sample. Aspect ratios and clamping modifications are highlighted (light and dark grey respectively).

First Author, Last name	Year	Human Soft-Tissue Sample	Width W_0 [mm]	Thickness [mm]	Cross-sectional Area [mm²]	Gauge Length L_1 [mm]	Aspect Ratio $L_1 : W_0$	Clamping Modifications
Stabile (Stabile et al., 2004)	2004	Membrane/ligament (forearm; interosseous membrane; transverse and longitudinal)	4 (transverse)<br>2 (longitudinal)	5.7 (transverse)<br>2.5 (longitudinal)		6 (transverse)<br>10 (longitudinal)	1.5 (non-tapered, transverse)<br>5 (tapered, longitudinal)	Cotton gauze
Sokolis (Sokolis et al., 2012b)	2012	Aorta (thoracic, ascending; aneurysm; tricuspid aortic valve dysfunction)	10 (non-tapered)<br>2 (tapered)			25 (tapered)	Non-tapered<br>12.5 (tapered)	Sandpaper, cyanoacrylate glue
Sokolis (Sokolis et al., 2012a)	2012	Aorta (thoracic, ascending; aneurysm; tricuspid aortic valve dysfunction)	10 (non-tapered)<br>2 (tapered)			25 (tapered)	Non-tapered<br>12.5 (tapered)	Sandpaper, cyanoacrylate glue
Sokolis (Sokolis and Iliopoulos, 2014)	2014	Aorta (thoracic, ascending; aneurysm)	10 (non-tapered)<br>2 (tapered)			7 (tapered)	Non-tapered<br>3.5 (tapered)	Sandpaper, cyanoacrylate glue
Sang (Sang et al., 2018)	2018	Artery (neck; carotid)	2.4 (non-tapered)<br>1.2 (tapered)	0.32	0.77 (non-tapered)<br>0.38 (tapered)			Sandpaper, foam tape
Sokolis (Sokolis and Angouras, 2021)	2021	Aorta (thoracic; ascending, aneurysm; aortic stenosis, aortic insufficiency, tricuspid aortic valve dysfunction)	10 (non-tapered)<br>2 (tapered)			7	Non-tapered<br>3.5 (tapered)	Sandpaper, cyanoacrylate glue
Zwirner (Zwirner et al., 2021a)	2021	Tendon (foot; plantaris muscle and peroneus tertius muscles)<br>Ligament (ankle; medial or ML and lateral LL)			2.7 (plantaris)<br>5.0 (peroneus),<br>10.9 (medial)<br>12.7 (lateral)	21 (non-tapered)<br>21 (tapered, ligament)	Non-tapered<br>4 (tapered, ML)<br>4 (tapered, LL)<br>!SO 527-2:1996	3D-printed pyramids

Supplementary Table 6

Included studies using sample shapes other than tapered or non-tapered, arranged by publication year and last name of the first author. All retrieved measurements are from the gauge section of the sample. Clamping modifications are highlighted (dark grey respectively).

Reference	Year	Human Soft-tissue Sample	Width, Diameter, or Circumference [mm]	Thickness [mm]	Cross-sectional Area [mm²]	Gauge Length L_1 [mm]	Sample Shape	Clamping Modifications
Lee (Lee and Haut, 1989)	1989	Vein (intracranial; parasagittal)	4.4 (circumference)	0.05 (wall)		5	Tube	Sandpaper
Bilston (Bilston and Thibault, 1996)	1996	Spinal cord (cervical and thoracic)	10.58 (diameter)		87.93	35.71	Cylinder	Cyanoacrylate glue, plastic mounting plates
Zarzur (Zarzur, 1996)	1996	Dura matter (vertebral; lumbar)	20				Square	
Schechtman (Schechtman and Bader, 1997)	1997	Tendon (forearm; extensor digitorum longus muscle)			2.91	72	Cylinder	
Monson (Monson et al., 2003)	2003	Artery and vein (intracranial; cerebral; temporal lobe)	0.52 (artery diameter)<br>0.71 (vein diameter)	0.10 (artery)<br>0.12 (vein)	0.169 (artery)<br>0.335 (vein)		Cylinder	Sandpaper
DeFrate (DeFrate et al., 2004)	2004	Tendon (leg; Achilles)	11 (diameter)				Cylinder	Sandpaper
Haraldsson (Haraldsson et al., 2005)	2005	Tendon (knee; patellar)	0.3 to 0.4 (diameter)				Cylinder	Cyanoacrylate glue
Haraldsson (Haraldsson et al., 2008)	2008	Ligament (knee; anterior cruciate or ACL)<br>Tendon (leg; Achilles)	0.3 to 0.76 (diameter)				Cylinder (ACL)<br>Cylinder (Achilles)	Cyanoacrylate glue
Hansen (Hansen et al., 2010)	2010	Tendon (knee; patellar)	0.3 to 0.4 (diameter)			15<br>10	Cylinder	Cyanoacrylate glue
Oxlund (Oxlund et al., 2010a)	2010	Cervix (pelvis)	2 (diameter)			4	Cylinder	
Oxlund (Oxlund et al., 2010b)	2010	Cervix (pelvis)	2 (diameter)			4	Cylinder	
Hanson (Hanson et al., 2012)	2012	Tendon (leg; iliopsoas muscle and Achilles)	0.7 (diameter)			10	Cylinder	Cyanoacrylate glue
Hou (Hou et al., 2013)	2013	Vein (fetal; umbilical)<br>Artery (intracranial; middle cerebral)	2.92 to 3.06 (vein diameter)<br>3.02 to 3.10 (artery diameter)	0.198 to 0.206 (vein)<br>0.201 to 0.212 (artery)			Cylinder	

Davis (Davis et al., 2016)	2016	Artery (neck; carotid; atherosclerotic fibrous cap)		5			Single-notched tensile bar	Sandpaper
Fomikina (Fomkina et al., 2016)	2016	Artery (intracranial; cerebellar)					Cylinder	
Hohmann (Hohmann et al., 2019)	2019	Tendon (arm; biceps, long head)	8.7 (fresh, diameter)<br>5.6 (fresh-frozen, diamater)<br>5.1 (Thiel, diameter)<br>4.6 (formalin, diameter)		4.0 (fresh)<br>2.3 (fresh-frozen)<br>2.2 (Thiel)<br>2.6 (formalin)	20	Cylinder	
Jacquet (Jacquet et al., 2019)	2019	Meniscus (knee; lateral)					Wedge	
Stouthandel (Stouthandel et al., 2020)	2020	Nerve (arm; median)					Cylinder	
Gaecia-Vilana (García-Vilana and Sánchez-Molina, 2022)	2022	Vein (intercranial; cerebral bridging)					Cylinder	
Scholze (Scholze et al., 2022)	2022	Tendon (arm; flexor digitorum profundus and flexor digitorum superficialis muscles)			13.42	40	Cylinder	

Supplementary Table 7

Included studies with no sample shape specified, arranged by publication year and last name of the first author. All retrieved measurements are from the gauge section of the sample. Clamping modifications are highlighted (dark grey).

Reference	Year	Human Soft-Tissue Sample	Width W_0 [mm]	Thickness [mm]	Cross sectional Area [mm²]	Gauge Length L_1 [mm]	Aspect Ratio $L_1 : W_0$	Clamping Modifications
Engin (Engin and Akkas, 1983)	1983	Peritoneum (intraabdominal)						
Breault-Janicki (Breault-Janicki et al., 1998)	1998	Ligament (forearm; extensor carpi radialis longus or brevis, or extensor pollicis longus; rheumatoid and 6 non-rheumatoid arthritis;)				15		
Tanaka (Tanaka et al., 2000)	2000	Intraarticular disc (jaw; (temporomandibular)						
Barber (Barber et al., 2001)	2001	Mitral valve leaflet (heart; myxomatous and normal; radial and circumferential)	10					Sandpaper
Kang (Kang et al., 2006)	2006	Intraarticular disc (jaw; (temporomandibular)	2					
Bursac (Bursac et al., 2009)	2009	Meniscus (knee; lateral and medial)	6.2	3.7		30		
Zimmern (Zimmern et al., 2009)	2009	Ligament (knee, anterolateral ligament			1.54	33.1		
Lawlor (Lawlor et al., 2011)	2010	Joint capsule (shoulder; glenohumeral)						
Trabelsi (Trabelsi et al., 2010)	2010	Trachea (neck)	6	2.1				
Wang (Wang et al., 2010)	2010	Tendon (leg; semitendinosus muscle)			20.44	30		
Svensson (Svensson et al., 2011)	2011	Tendon (knee; patellar)						
Voycheck (Voycheck et al.,	2011	Artery (neck; carotid; plaques)						

2010)								
Brandt<br>(Brandt et al., 2012)	2012	Ligament (facial; retaining; orbital, zygomatic, maxillary, and mandibular)	6.7 (orbital)<br>6.8 (zygomatic)<br>7.0 (maxillary)<br>6.3 (mandibular)	2.0 (orbital)<br>3.1 (zygomatic)<br>2.7 (maxillary)<br>2.5 (mandibular)	13.4 (orbital)<br>21.08 (zygomatic)<br>14.7 (maxillary)<br>15.75 (mandibular)	10.9, (orbital)<br>12.2 (zygomatic)<br>12.7 (maxillary)<br>10.6 (mandibular)	1.63 (orbital<br>1.79 (zygomatic)<br>1.81 (maxillary)<br>1.68 (mandibular)	Suture
Casado<br>(Casado et al., 2012)	2012	Mitral chordae tendineae (heart)			0.35	11		
Rainis<br>(Rainis et al., 2012)	2012	Joint capsule (knee)						
Chebil<br>(Chebil et al., 2013)	2013	Gastrocolic ligament (abdomen)	25					Sandpaper
Gultova<br>(Gultova et al., 2013)	2013	Pericardium (heart)						
Jayyosi<br>(Jayyosi et al., 2013)	2013	Liver (abdomen; Glisson capsule)						
Karimi<br>(Karimi et al., 2013)	2013	Artery (heart; coronary)						
Ma<br>(Ma et al., 2013)	2013	Nerve (arm; ulnar and median)				22 (median)<br>44 (ulnar)		
Chantereau<br>(Chantereau et al., 2014)	2014	Pelvic floor (pelvis)						
Monea<br>(Monea et al., 2014)	2014	Vein (intracranial; bridging vein-superior sagittal sinus complex)		0.04				Laparoscopic grasps
Swank<br>(Swank et al., 2014)	2014	Tendon (leg; tibialis posterior)			22.4	32		
Tantius<br>(Tantius et al., 2014)	2014	Umbilical cord				15.8 (5 to 35)		
Karimi<br>(Karimi et al., 2015b)	2015	Artery (heart; coronary)						
Karimi<br>(Karimi et al., 2015a)	2015	Vein (leg; saphenous)						
Liao<br>(Liao et al., 2015)	2015	Tendon (leg; peroneus longus and brevis muscles; calcaneal)						Tissue paper

		Ligament (calcaneofibular)						
Rassoli (Rassoli et al., 2015)	2015	Vein (leg; saphenous)		0.50				
Weber (Weber et al., 2015)	2015	Tendon (forearm; numerous muscles)			0.45 to 20.61			Cryoclamp
Abdalbary (Abdalbary et al., 2016)	2016	Ligament (foot; deep transverse metatarsal; hallux valgus and normal)			3.0 (normal)<br>1.6 (hallux valgus)			
Karimi (Karimi et al., 2016)	2016	Artery (heart; coronary; atherosclerotic and normal)						Sandpaper
Safshekan (Safshekan et al., 2016)	2016	Trachea (neck; cartilage, smooth muscle or SM, connective tissue or CT)				4.13 (cartilage)<br>6.37 (SM)<br>1.60 (CT)		
Karimi (Karimi et al., 2017)	2017	Gallbladder (abdomen)						
Signorelli (Signorelli et al., 2018)	2018	Aneurysm (intracranial; ruptured and unruptured)				3		
Brečs (Brečs et al., 2019)	2019	Aorta (thoracic, ascending; dilated; longitudinal and circumferential)	5	2.35 (longitudinal)<br>2.34 (circumferential)				
Smith (Smith et al., 2019)	2019	Ligament (forearm; ulnar collateral, anterior and posterior bundles)		1.1				Sandpaper, cyanoacrylate glue
Castile (Castile et al., 2020)	2020	Ligament (knee; lateral collateral or LCL; anterolateral or ALL, anterolateral capsule or ALC)	9.5 to 11 (ALL)<br>18.4 to 20.8 (ALC)	1				Aluminum beads, cyanoacrylate glue
Chivot (Chivot et al., 2020)	2020	Tendon (leg; quadriceps)		1.80 to12.3				
Jaquet (Jacquet et al., 2020)	2020	Tendon (leg; semitendinosus muscle)						
Kadlub (Kadlub et al., 2020)	2020	Periosteum (head/jaw; mandibular, calvarial)	10					
Thomas (Thomas et al., 2020)	2020	Tendon (foot/ankle; flexor hallucis capsularis Interphalangeus)			2	10		Sandpaper
Ekiert (Ekiert et al.,	2021	Tendon (leg; Achilles; soleus and gastrocnemius				80		Sandpaper, cellulose wadding

2021)		muscles)						swab
Gatt<br>(Gatt et al., 2021)	2021	Tendon (leg; Achilles, tibialis posterior and peroneus longus muscles)						Cloth
Kholinne<br>(Kholinne et al., 2021)	2021	Interosseous membrane (forearm)						Toothed-clamps
Lisicky<br>(Lisický et al., 2021)	2021	Atheroma (neck; carotid artery)		0.87 (women)<br>0.98 (men)				
Zellers<br>(Zellers et al., 2021)	2021	Tendon (leg; Achilles)		1	19.14			Sandpaper
Strauss<br>(Strauss et al., 2022)	2022	Tendon (leg; quadriceps)	10 to 12			25		Nylon tubing

Supplementary Table 8

International Organization for Standardization (ISO) standards of sample shape and dimensions used for mechanical testing of engineering materials. All retrieved measurements are from the gauge section of the sample.

ISO Standard	Material	Sample Shape	Width W_0 [mm]	Thickness [mm]	Cross sectional Area [mm²]	Gauge Length L_1 [mm]	Overall Length [mm]	Aspect Ratio $L_1 : W_0$
ISO 527-2 (Standardization, 2012)	Polymer	Tapered	10	4	40	75	170	7.50
	Polymer	Tapered	10	4	40	50	150	5.00
	Polymer	Tapered	5	2	10	25	75	5.00
	Polymer	Tapered	2	2	4	10	30	5.00
	Polymer	Tapered	4	2	8	20	75	5.00
	Polymer	Tapered	2	1	2	10	35	5.00
ISO 527-3 (Standardization, 2018)	Polymer	Tapered	6	1	6	25	115	4.17
	Polymer	Tapered	10	1	10	50	150	5.00
	Polymer	Tapered	25.4	1	25.4	50	152	1.97
	Polymer	Non-tapered	10	1	10	50	150	5.00
	Polymer	Non-tapered	15	1	15	50	150	3.33
ISO 1421 (Standardization, 2016)	Rubber- or plastic-coated fabrics	Non-tapered	10			100	220	10.00
	Rubber- or plastic-coated fabrics	Non-tapered	30			150	260	5.00

	Rubber- or plastic-coated fabrics	Non-tapered	50			200	300	4.00
ISO 527-4 (Standardization, 2021a)	Composite	Non-tapered	25	2 to 10	50 to 250	150	250	6.00
	Composite	Non-tapered	50	2 to 10	100 to 500	150	250	3.00
ISO 527-5 (Standardization, 2021b)	Composite	Non-tapered	15	1	15	150	250	10.00
	Composite	Non-tapered	25	2	50	150	250	6.00
ISO 3376 (Standardization, 2020)	Leather	Tapered	10			50	110	5.00
	Leather	Tapered	20			100	190	5.00
ISO 13061-6 (Standardization, 2014)	Wood	Tapered	20	4	80	90	350	4.50

SUPPLEMENTARY REFERENCES

Abdalbary, S.A., Elshaarawy, E.A.A., and Khalid, B.E.A. (2016). Tensile Properties of the Deep Transverse Metatarsal Ligament in Hallux Valgus: A CONSORT-Compliant Article. *Medicine (Baltimore)* 95(8)**,** e2843. doi: 10.1097/md.0000000000002843.

Abraham, A.C., Moyer, J.T., Villegas, D.F., Odegard, G.M., and Haut Donahue, T.L. (2011). Hyperelastic properties of human meniscal attachments. *J Biomech* 44(3)**,** 413-418. doi: 10.1016/j.jbiomech.2010.10.001.

Abramowitch, S.D., Zhang, X., Curran, M., and Kilger, R. (2010). A comparison of the quasi-static mechanical and non-linear viscoelastic properties of the human semitendinosus and gracilis tendons. *Clin Biomech (Bristol, Avon)* 25(4)**,** 325-331. doi: 10.1016/j.clinbiomech.2009.12.007.

Afoke, A., Meagher, P.J., Starley, I., McGrouther, D.A., Bailey, A.J., and Brown, R.A. (1998). Biomechanical Characterization of Tissues in Dupuytren's Disease. *J Hand Surg Br* 23(3)**,** 291-296. doi: 10.1016/s0266-7681(98)80043-0.

Aisa, R., Arezu, A.M., Nasser, F., Saeed, S.A., and Mohammad, S. (2016). A Structural Constitutive Model For Viscoelastic Rheological Behavior Of Human Saphenous Vein Using Experimental Assays. *International Journal of Biology and Biomedical Engineering* 10(3)**,** 170-173. doi: 10.5281/zenodo.1130397.

Akizuki, S., Mow, V.C., Muller, F., Pita, J.C., and Howell, D.S. (1987). Tensile properties of human knee joint cartilage. II. Correlations between weight bearing and tissue pathology and the kinetics of swelling. *J Orthop Res* 5(2)**,** 173-186. doi: 10.1002/jor.1100050204.

Akizuki, S., Mow, V.C., Müller, F., Pita, J.C., Howell, D.S., and Manicourt, D.H. (1986). Tensile properties of human knee joint cartilage: I. Influence of ionic conditions, weight bearing, and fibrillation on the tensile modulus. *J Orthop Res* 4(4)**,** 379-392. doi: 10.1002/jor.1100040401.

Amabili, M., Arena, G.O., Balasubramanian, P., Breslavsky, I.D., Cartier, R., Ferrari, G., et al. (2020). Biomechanical characterization of a chronic type a dissected human aorta. *Journal of Biomechanics* 110. doi: 10.1016/j.jbiomech.2020.109978.

Amabili, M., Balasubramanian, P., Bozzo, I., Breslaysky, I.D., and Ferrari, G. (2019). Layer-specific hyperelastic and viscoelastic characterization of human descending thoracic aortas. *Journal of the Mechanical Behavior of Biomedical Materials* 99**,** 27-46. doi: 10.1016/j.jmbbm.2019.07.008.

Andreassen, T.T., Simonsen, A.H., and Oxlund, H. (1980). Biomechanical properties of keratoconus and normal corneas. *Exp Eye Res* 31(4)**,** 435-441. doi: 10.1016/s0014-4835(80)80027-3.

Antun, S., Tomislav, B., Tomislav, S., and Robert, S. (2017). Biomechanics of fetal membranes - relation with newborn and maternal anthropometric data. *Periodicum Biologorum* 119(2)**,** 119-123. doi: 10.18054/pb.v119i2.4660.

Astruc, L., De Meulaere, M., Witz, J.F., Nováček, V., Turquier, F., Hoc, T., et al. (2018). Characterization of the anisotropic mechanical behavior of human abdominal wall connective tissues. *J Mech Behav Biomed Mater* 82**,** 45-50. doi: 10.1016/j.jmbbm.2018.03.012.

Atienza, J.M. (2010). Response of human coronary arteries at different mechanical conditions. *Annu Int Conf IEEE Eng Med Biol Soc* 2010**,** 3585-3588. doi: 10.1109/iembs.2010.5627456.

Aydin, H., Kizmazoglu, C., Kaya, I., Husemoglu, B., Sozer, G., Havitcioglu, H., et al. (2019). Biomechanical Properties of the Cranial Dura Mater with Puncture Defects : An In Vitro Study. *JOURNAL OF KOREAN NEUROSURGICAL SOCIETY* 62(4)**,** 382-388.

Bae, W.C., Wong, V.W., Hwang, J., Antonacci, J.M., Nugent-Derfus, G.E., Blewis, M.E., et al. (2008). Wear-lines and split-lines of human patellar cartilage: relation to tensile biomechanical properties. *Osteoarthritis Cartilage* 16(7)**,** 841-845. doi: 10.1016/j.joca.2007.11.015.

Bai, Z., Gu, J., Shi, Y., and Meng, W. (2018). Effect of inflammation on the biomechanical strength of involved aorta in type A aortic dissection and ascending thoracic aortic aneurysm: An initial research. *Anatol J Cardiol* 20(2)**,** 85-92. doi: 10.14744/AnatolJCardiol.2018.49344.

Baldit, A., Dubus, M., Sergheraert, J., Kerdjoudj, H., Mauprivez, C., and Rahouadj, R. (2022). Biomechanical tensile behavior of human Wharton's jelly. *Journal of the Mechanical Behavior of Biomedical Materials* 126**,** 104981. doi: 10.1016/j.jmbbm.2021.104981.

Banks, J., Booth, F.V., MacKay, E.H., Rajagopalan, B., and Lee, G.D. (1978). The physcial properties of human pulmonary arteries and veins. *Clin Sci Mol Med* 55(5)**,** 477-484. doi: 10.1042/cs0550477.

Barber, J.E., Kasper, F.K., Ratliff, N.B., Cosgrove, D.M., Griffin, B.P., and Vesely, I. (2001). Mechanical properties of myxomatous mitral valves. *J Thorac Cardiovasc Surg* 122(5)**,** 955-962. doi: 10.1067/mtc.2001.117621.

Barrett, H.E., Cunnane, E.M., Kavanagh, E.G., and Walsh, M.T. (2016). On the effect of calcification volume and configuration on the mechanical behaviour of carotid plaque tissue. *Journal of the Mechanical Behavior of Biomedical Materials* 56**,** 45-56. doi: 10.1016/j.jmbbm.2015.11.001.

Bellucci, G., and Seedhom, B.B. (2001). Mechanical behaviour of articular cartilage under tensile cyclic load. *Rheumatology* 40(12)**,** 1337-1345. doi: 10.1093/rheumatology/40.12.1337.

Ben Abdelounis, H., Nicolle, S., Ottenio, M., Beillas, P., and Mitton, D. (2013). Effect of two loading rates on the elasticity of the human anterior rectus sheath. *J Mech Behav Biomed Mater* 20**,** 1-5. doi: 10.1016/j.jmbbm.2012.12.002.

Bhat, S.K., Sakata, N., and Yamada, H. (2020). Identification of uniaxial deformation behavior and its initial tangent modulus for atheromatous intima in the human carotid artery and thoracic aorta using three-parameter isotropic hyperelastic models. *Journal of Mechanics in Medicine and Biology* 20(3)**,** 25. doi: 10.1142/s0219519420500141.

Bilston, L.E., and Thibault, L.E. (1996). The mechanical properties of the human cervical spinal cord in vitro. *Annals of Biomedical Engineering* 24(1)**,** 67-74.

Birch, M., and Srodon, P. (2009). Biomechanical Properties of the Human Soft Palate. *Cleft Palate Craniofacial Journal* 46(3)**,** 268-274. doi: 10.1597/08-012.1.

Bourgouin, S., Bege, T., Masson, C., Arnoux, P.J., Mancini, J., Garcia, S., et al. (2012). Biomechanical characterisation of fresh and cadaverous human small intestine: applications for abdominal trauma. *Medical & Biological Engineering & Computing* 50(12)**,** 1279-1288. doi: 10.1007/s11517-012-0964-y.

Brandt, M.G., Hassa, A., Roth, K., Wehrli, B., and Moore, C.C. (2012). Biomechanical properties of the facial retaining ligaments. *Arch Facial Plast Surg* 14(4)**,** 289-294. doi: 10.1001/archfacial.2011.1533.

Breault-Janicki, M.J., Small, C.F., Bryant, J.T., Dwosh, I.L., Lee, J.M., and Pichora, D.R. (1998). Mechanical properties of wrist extensor tendons are altered by the presence of rheumatoid arthritis. *J Orthop Res* 16(4)**,** 472-474. doi: 10.1002/jor.1100160412.

Brečs, I., Stradiņš, P., Kalējs, M., Strazdiņš, U., Ozolanta, I., and Kasyanov, V. (2019). Biomechanical Properties of Human Dilated Ascending Aorta. *Proceedings of the Latvian Academy of Sciences. Section B. Natural, Exact, and Applied Sciences* 73(2 (719))**,** 101-111. doi: 10.2478/prolas-2019-0017.

Brieu, M., Chantereau, P., Gillibert, J., de Landsheere, L., Lecomte, P., and Cosson, M. (2016). A nonlinear-elastic constitutive model for soft connective tissue based on a histologic description: Application to female pelvic soft tissue. *J Mech Behav Biomed Mater* 58**,** 65-74. doi: 10.1016/j.jmbbm.2015.09.023.

Brunel, H., Ambard, D., Dufour, H., Roche, P.H., Costalat, V., and Jourdan, F. (2018). Rupture limit evaluation of human cerebral aneurysms wall: Experimental study. *Journal of Biomechanics* 77**,** 76-82. doi: 10.1016/j.jbiomech.2018.06.016.

Brunenieks, I., Pekarska, K., Kasyanov, V., and Groma, V. (2017). Biomechanical and morphological peculiarities of the rectum in patients with obstructed defecation syndrome. *Romanian Journal of Morphology and Embryology* 58(4)**,** 1193-1200.

Bursac, P., York, A., Kuznia, P., Brown, L.M., and Arnoczky, S.P. (2009). Influence of Donor Age on the Biomechanical and Biochemical Properties of Human Meniscal Allografts. *Am J Sports Med* 37(5)**,** 884-889. doi: 10.1177/0363546508330140.

Butler, A.M., and Walsh, W.R. (2004). Mechanical response of ankle ligaments at low loads. *Foot & Ankle International* 25(1)**,** 8-12. doi: 10.1177/107110070402500103.

Carniel, E.L., Albanese, A., Fontanella, C.G., Pavan, P.G., Prevedello, L., Salmaso, C., et al. (2020). Biomechanics of stomach tissues and structure in patients with obesity. *Journal of the Mechanical Behavior of Biomedical Materials* 110. doi: 10.1016/j.jmbbm.2020.103883.

Carpenter, J.E., Wening, J.D., Mell, A.G., Langenderfer, J.E., Kuhn, J.E., and Hughes, R.E. (2005). Changes in the long head of the biceps tendon in rotator cuff tear shoulders. *Clin Biomech (Bristol, Avon)* 20(2)**,** 162-165. doi: 10.1016/j.clinbiomech.2004.09.009.

Casado, J.A., Diego, S., Ferreno, D., Ruiz, E., Carrascal, I., Mendez, D., et al. (2012). Determination of the mechanical properties of normal and calcified human mitral chordae tendineae. *J Mech Behav Biomed Mater* 13**,** 1-13. doi: 10.1016/j.jmbbm.2012.03.016.

Castile, R., Lake, S., Brophy, R., and Patel, R. (2020). Microstructural and Mechanical Properties of the Anterolateral Ligament (ALL) of the Knee.

Celi, S., Losi, P., and Berti, S. (2012). Investigation on regional variation of intraluminal thrombus: A mechanical and histological study. *Bioinspired Biomimetic and Nanobiomaterials* 1(3)**,** 183-194. doi: 10.1680/bbn.12.00006.

Chang, P.S., Solon, L.F., Lake, S.P., Castile, R.M., Hill, J.R., and Brophy, R.H. (2022). Mechanical and Microstructural Properties of Meniscus Roots Vary by Location. *Am J Sports Med* 50(10)**,** 2733-2739. doi: 10.1177/03635465221106746.

Chantereau, P., Brieu, M., Kammal, M., Farthmann, J., Gabriel, B., and Cosson, M. (2014). Mechanical properties of pelvic soft tissue of young women and impact of aging. *Int Urogynecol J* 25(11)**,** 1547-1553. doi: 10.1007/s00192-014-2439-1.

Chauvet, D., Carpentier, A., Allain, J.M., Polivka, M., Crepin, J., and George, B. (2010). Histological and biomechanical study of dura mater applied to the technique of dura splitting decompression in Chiari type I malformation. *Neurosurgical Review* 33(3)**,** 287-294. doi: 10.1007/s10143-010-0261-x.

Chebil, O., Arnoux, P.J., and Behr, M. (2015). Mechanical characterization of human gastrocolic ligament until failure. *J Appl Biomater Funct Mater* 13(2)**,** e106-115. doi: 10.5301/jabfm.5000193.

Chebil, O., Behr, M., and Arnoux, P.-J. (2013). Quasi-static failure properties of the human gastrocolic ligament. *Computer Methods in Biomechanics and Biomedical Engineering in Medicine* 15**,** 283–284. doi: 10.1080/10255842.2012.713631.

Cheng, T., Dai, C., and Gan, R.Z. (2007). Viscoelastic properties of human tympanic membrane. *Ann Biomed Eng* 35(2)**,** 305-314. doi: 10.1007/s10439-006-9227-0.

Chivot, M., Pioger, C., Cognault, J., Sharma, A., Pailhé, R., Cavaignac, E., et al. (2020). Every layer of quadriceps tendon’s central and medial portion offers similar tensile properties than Hamstrings or Ilio-Tibial Band Grafts. *Experimental Orthopaedics* 7(1)**,** 50-57.

Choi, J.J.E., Zwirner, J., Ramani, R.S., Ma, S., Hussaini, H.M., Waddell, J.N., et al. (2020). Mechanical properties of human oral mucosa tissues are site dependent: A combined biomechanical, histological and ultrastructural approach. *Clin Exp Dent Res* 6(6)**,** 602-611. doi: 10.1002/cre2.305.

Claes, E., Atienza, J.M., Guinea, G.V., Rojo, F.J., Bernal, J.M., Revuelta, J.M., et al. (2010). Mechanical properties of human coronary arteries. *Annu Int Conf IEEE Eng Med Biol Soc* 2010**,** 3792-3795. doi: 10.1109/IEMBS.2010.5627560.

Clavert, P., Kempf, J.F., Bonnomet, F., Boutemy, P., Marcelin, L., and Kahn, J.L. (2001). Effects of freezing/thawing on the biomechanical properties of human tendons. *Surg Radiol Anat* 23(4)**,** 259-262. doi: 10.1007/s00276-001-0259-8.

Clay, J.-C., Rubod, C., Brieu, M., Boukerrou, M., Fasel, J., and Cosson, M. (2011). Biomechanical properties of prolapsed or non-prolapsed vaginal tissue: impact on genital prolapse surgery. *Int Urogynecol J*. doi: 10.1007/s00192-010-1208-z.

Cooney, G.M., Lake, S.P., Thompson, D.M., Castile, R.M., Winter, D.C., and Simms, C.K. (2016). Uniaxial and biaxial tensile stress-stretch response of human linea alba. *J Mech Behav Biomed Mater* 63**,** 134-140. doi: 10.1016/j.jmbbm.2016.06.015.

Costalat, V., Sanchez, M., Ambard, D., Thines, L., Lonjon, N., Nicoud, F., et al. (2011). Biomechanical wall properties of human intracranial aneurysms resected following surgical clipping (IRRAs Project). *Journal of Biomechanics* 44(15)**,** 2685-2691. doi: 10.1016/j.jbiomech.2011.07.026.

Courtial, E.J., Fanton, L., Orkisz, M., Douek, P.C., Huet, L., and Fulchiron, R. (2016). Hyper-Viscoelastic Behavior of Healthy Abdominal Aorta. *Irbm* 37(3)**,** 158-164. doi: 10.1016/j.irbm.2016.03.007.

Craiem, D., Rojo, F.J., Atienza, J.M., Armentano, R.L., and Guinea, G.V. (2008). Fractional-order viscoelasticity applied to describe uniaxial stress relaxation of human arteries. *Physics in Medicine and Biology* 53(17)**,** 4543-4554. doi: 10.1088/0031-9155/53/17/006.

Criscenti, G., De Maria, C., Sebastiani, E., Tei, M., Placella, G., Speziali, A., et al. (2016). Material and structural tensile properties of the human medial patello-femoral ligament. *Journal of the Mechanical Behavior of Biomedical Materials* 54**,** 141-148. doi: 10.1016/j.jmbbm.2015.09.030.

Daly, C.H. (1982). Biomechanical properties of dermis. *J Invest Dermatol* 79 Suppl 1**,** 17s-20s. doi: 10.1111/1523-1747.ep12544620.

Davis, L.A., Stewart, S.E., Carsten, C.G., 3rd, Snyder, B.A., Sutton, M.A., and Lessner, S.M. (2016). Characterization of fracture behavior of human atherosclerotic fibrous caps using a miniature single edge notched tensile test. *Acta Biomater* 43**,** 101-111. doi: 10.1016/j.actbio.2016.07.027.

Davis, P.A., and Wastell, C. (2000). A comparison of biomechanical properties of excised mature scars from HIV patients and non-HIV controls. *Am J Surg* 180(3)**,** 217-222. doi: 10.1016/s0002-9610(00)00468-2.

de Landsheere, L., Brieu, M., Blacher, S., Munaut, C., Nusgens, B., Rubod, C., et al. (2016). Elastin density: Link between histological and biomechanical properties of vaginal tissue in women with pelvic organ prolapse? *Int Urogynecol J* 27(4)**,** 629-635. doi: 10.1007/s00192-015-2901-8.

Defrate, L.E., van der Ven, A., Boyer, P.J., Gill, T.J., and Li, G. (2006). The measurement of the variation in the surface strains of Achilles tendon grafts using imaging techniques. *J Biomech* 39(3)**,** 399-405. doi: 10.1016/j.jbiomech.2004.12.021.

DeFrate, L.E., van der Ven, A., Gill, T.J., and Li, G. (2004). The effect of length on the structural properties of an Achilles tendon graft as used in posterior cruciate ligament reconstruction. *Am J Sports Med* 32(4)**,** 993-997. doi: 10.1177/0363546503261739.

Deveja, R.P., Iliopoulos, D.C., Kritharis, E.P., Angouras, D.C., Sfyris, D., Papadodima, S.A., et al. (2018). Effect of Aneurysm and Bicuspid Aortic Valve on Layer-Specific Ascending Aorta Mechanics. *Ann Thorac Surg* 106(6)**,** 1692-1701. doi: 10.1016/j.athoracsur.2018.05.071.

Di Martino, E., Mantero, S., Inzoli, F., Melissano, G., Astore, D., Chiesa, R., et al. (1998). Biomechanics of abdominal aortic aneurysm in the presence of endoluminal thrombus: experimental characterisation and structural static computational analysis. *Eur J Vasc Endovasc Surg* 15(4)**,** 290-299. doi: 10.1016/s1078-5884(98)80031-2.

Di Martino, E.S., Bohra, A., Vande Geest, J.P., Gupta, N., Makaroun, M.S., and Vorp, D.A. (2006). Biomechanical properties of ruptured versus electively repaired abdominal aortic aneurysm wall tissue. *J Vasc Surg* 43(3)**,** 570-576; discussion 576. doi: 10.1016/j.jvs.2005.10.072.

Dodson, R.B., Martin, J.T., Hunter, K.S., and Ferguson, V.L. (2013). Determination of hyperelastic properties for umbilical artery in preeclampsia from uniaxial extension tests. *Eur J Obstet Gynecol Reprod Biol* 169(2)**,** 207-212. doi: 10.1016/j.ejogrb.2013.03.010.

Donovan, D.L., Schmidt, S.P., Townshend, S.P., Njus, G.O., and Sharp, W.V. (1990). Material and structural characterization of human saphenous vein. *J Vasc Surg* 12(5)**,** 531-537. doi: 10.1016/0741-5214(90)90005-U.

Duprey, A., Khanafer, K., Schlicht, M., Avril, S., Williams, D., and Berguer, R. (2010). In Vitro Characterisation of Physiological and Maximum Elastic Modulus of Ascending Thoracic Aortic Aneurysms Using Uniaxial Tensile Testing. *Eur J Vasc Endovasc Surg* 39**,** 700-707. doi: 10.1016/j.ejvs.2010.02.015.

Durcan, C., Hossain, M., Chagnon, G., Perić, D., Karam, G., Bsiesy, L., et al. (2022). Experimental investigations of the human oesophagus: anisotropic properties of the embalmed mucosa-submucosa layer under large deformation. *Biomech Model Mechanobiol* 21(6)**,** 1685-1702. doi: 10.1007/s10237-022-01613-1.

Ebara, S., Iatridis, J.C., Setton, L.A., Foster, R.J., Mow, V.C., and Weidenbaum, M. (1996). Tensile properties of nondegenerate human lumbar anulus fibrosus. *Spine* 21(4)**,** 452-461. doi: 10.1097/00007632-199602150-00009.

Egorov, V.I., Schastlivtsev, I.V., Prut, E.V., Baranov, A.O., and Turusov, R.A. (2002). Mechanical properties of the human gastrointestinal tract. *Journal of Biomechanics* 35(10)**,** 1417-1425. doi: 10.1016/s0021-9290(02)00084-2.

Ekiert, M., Tomaszewski, K.A., and Mlyniec, A. (2021). The differences in viscoelastic properties of subtendons result from the anatomical tripartite structure of human Achilles tendon - ex vivo experimental study and modeling. *Acta Biomater* 125**,** 138-153. doi: 10.1016/j.actbio.2021.02.041.

Elliott, D.M., and Setton, L.A. (2001). Anisotropic and inhomogeneous tensile behavior of the human anulus fibrosus: experimental measurement and material model predictions. *J Biomech Eng* 123(3)**,** 256-263. doi: 10.1115/1.1374202.

Elsheikh, A., Geraghty, B., Alhasso, D., Knappett, J., Campanelli, M., and Rama, P. (2010). Regional variation in the biomechanical properties of the human sclera. *Exp Eye Res* 90(5)**,** 624-633. doi: 10.1016/j.exer.2010.02.010.

Engin, A.E., and Akkas, N. (1983). Etiology and biomechanics of hernial sac formation. *J Biomed Eng* 5(4)**,** 329-335. doi: 10.1016/0141-5425(83)90009-2.

Falland-Cheung, L., Scholze, M., Lozano, P.F., Ondruschka, B., Tong, D.C., Brunton, P.A., et al. (2018). Mechanical properties of the human scalp in tension. *Journal of the Mechanical Behavior of Biomedical Materials* 84**,** 188-197. doi: 10.1016/j.jmbbm.2018.05.024.

Fatemifar, F., Feldman, M., Oglesby, M., and Han, H.C. (2018). Comparison of Biomechanical Properties and Microstructure of Trabeculae Carneae, Papillary muscles, and Myocardium in Human Heart. *J Biomech Eng* 141(2)**,** 210071-2100710. doi: 10.1115/1.4041966.

Faturechi, R., Hashemi, A., and Abolfathi, N. (2014). A tensile machine with a novel optical load cell for soft biological tissues application. *J Med Eng Technol* 38(8)**,** 411-415. doi: 10.3109/03091902.2014.968677.

Faturechi, R., Hashemi, A., and Fatouraee, N. (2015). Do mechanical properties of human fetal membrane depend on strain rate? *Journal of Obstetrics and Gynaecology Research* 41(1)**,** 84-91. doi: 10.1111/jog.12498.

Ferrara, A., Morganti, S., Totaro, P., Mazzola, A., and Auricchio, F. (2016). Human dilated ascending aorta: Mechanical characterization via uniaxial tensile tests. *Journal of the Mechanical Behavior of Biomedical Materials* 53**,** 257-271. doi: 10.1016/j.jmbbm.2015.08.021.

Ferrara, A., Totaro, P., Morganti, S., and Auricchio, F. (2018). Effects of clinico-pathological risk factors on in-vitro mechanical properties of human dilated ascending aorta. *Journal of the Mechanical Behavior of Biomedical Materials* 77**,** 1-11. doi: 10.1016/j.jmbbm.2017.08.032.

Ferro, F.P., Philippon, M.J., Rasmussen, M.T., Smith, S.D., LaPrade, R.F., and Wijdicks, C.A. (2015). Tensile properties of the human acetabular labrum and hip labral reconstruction grafts. *Am J Sports Med* 43(5)**,** 1222-1227. doi: 10.1177/0363546514568086.

Fessel, G., Frey, K., Schweizer, A., Calcagni, M., Ullrich, O., and Snedeker, J. (2011). Suitability of Thiel embalmed tendons for biomechanical investigation. *Annals of Anatomy - Anatomischer Anzeiger* 193(3), 237-241. doi: 10.1016/j.aanat.2011.03.007.

Fields, A.J., Rodriguez, D., Gary, K.N., Liebenberg, E.C., and Lotz, J.C. (2014). Influence of biochemical composition on endplate cartilage tensile properties in the human lumbar spine. *J Orthop Res* 32(2), 245-252. doi: 10.1002/jor.22516.

Fischenich, K., Lewis, J., Kindsfater, K., Bailey, T., and Donahue, T. (2015). Effects of degeneration on the compressive and tensile properties of human meniscus. *Journal of Biomechanics* 48(8), 1407-1411. doi: 10.1016/j.jbiomech.2015.02.042.

Fischer, B., Kurz, S., Hoch, A., and Schleifenbaum, S. (2020). The influence of different sample preparation on mechanical properties of human iliotibial tract. *Scientific Reports* 10(1). doi: 10.1038/s41598-020-71790-5.

Fomkina, O.A., Nikolenko, V.N., and Chernyshkova, E.V. (2016). Morphology and biomechanical properties of cerebellar arteries in adults. *Russian Open Medical Journal* 5(2), 4. doi: 10.15275/rusomj.2016.0205.

Forsell, C., Swedenborg, J., Roy, J., and Gasser, T.C. (2013). The quasi-static failure properties of the abdominal aortic aneurysm wall estimated by a mixed experimental-numerical approach. *Ann Biomed Eng* 41(7), 1554-1566. doi: 10.1007/s10439-012-0711-4.

Franchini, G., Breslavsky, I.D., Holzapfel, G.A., and Amabili, M. (2021). Viscoelastic characterization of human descending thoracic aortas under cyclic load. *Acta Biomaterialia* 130, 291-307. doi: 10.1016/j.actbio.2021.05.025.

Freutel, M., Scholz, N.B., Seitz, A.M., Ignatius, A., and Durselen, L. (2015). Mechanical properties and morphological analysis of the transitional zone between meniscal body and ligamentous meniscal attachments. *Journal of Biomechanics* 48(8), 1350-1355. doi: 10.1016/j.jbiomech.2015.03.003.

Gabriel, B., Rubod, C., Brieu, M., Dedet, B., de Landsheere, L., Delmas, V., et al. (2011). Vagina, abdominal skin, and aponeurosis: do they have similar biomechanical properties? *Int Urogynecol J* 22(1), 23-27. doi: 10.1007/s00192-010-1237-7.

Garcia-Herrera, C.M., Atienza, J.M., Rojo, F.J., Claes, E., Guinea, G.V., Celentano, D.J., et al. (2012a). Mechanical behaviour and rupture of normal and pathological human ascending aortic wall. *Medical & Biological Engineering & Computing* 50(6), 559-566. doi: 10.1007/s11517-012-0876-x.

Garcia-Herrera, C.M., Celentano, D.J., Cruchaga, M.A., Rojo, F.J., Atienza, J.M., Guinea, G.V., et al. (2012b). Mechanical characterisation of the human thoracic descending aorta: experiments and modelling. *Computer Methods in Biomechanics and Biomedical Engineering* 15(2), 185-193. doi: 10.1080/10255842.2010.520704.

García-Vilana, S., and Sánchez-Molina, D. (2022). Age effects on the mechanical behavior of human cerebral bridging veins. *Clin Biomech (Bristol, Avon)* 100**,** 105792. doi: 10.1016/j.clinbiomech.2022.105792.

Gatt, A., Chockalingam, N., Chatzistergos, P., Gatt, R., Schembri-Wismayer, P., Grima, J.N., et al. (2021). The biomechanical properties of human fresh-frozen vs thiel embalmed foot tendons. *Acta Scientific Orthopaedics* 4(11)**,** 19-23.

Gaur, P., Chawla, A., Verma, K., Mukherjee, S., Lalvani, S., Malhotra, R., et al. (2016). Characterisation of human diaphragm at high strain rate loading. *J Mech Behav Biomed Mater* 60**,** 603-616. doi: 10.1016/j.jmbbm.2016.02.031.

Gaur, P., Verma, K., Chawla, A., Mukherjee, S., Jain, M., Mayer, C., et al. (2020). A bilinear structural constitutive model for strain rate-dependent behaviour of human diaphragm tissue. *International Journal of Crashworthiness* 25(3)**,** 284-298. doi: 10.1080/13588265.2019.1583423.

Gawargious, B.A., Le, A., Lesgart, M., Ugardar, S., and Demer, J.L. (2020). Differential Regional Stiffening of Sclera by Collagen Cross-linking. *Current Eye Research* 45(6)**,** 718-725. doi: 10.1080/02713683.2019.1694157.

Geraghty, B., Jones, S.W., Rama, P., Akhtar, R., and Elsheikh, A. (2012). Age-related variations in the biomechanical properties of human sclera. *J Mech Behav Biomed Mater* 16**,** 181-191. doi: 10.1016/j.jmbbm.2012.10.011.

Giannini, S., Buda, R., Di Caprio, F., Agati, P., Bigi, A., De Pasquale, V., et al. (2008). Effects of freezing on the biomechanical and structural properties of human posterior tibial tendons. *Int Orthop* 32(2)**,** 145-151. doi: 10.1007/s00264-006-0297-2.

Gomes, V.C., da Silva, L.F.F., Silvestre, G.C., Queiroz, A., Marques, M.A., Zyngier, S.P., et al. (2021). Biomechanical Properties of the Periaortic Abdominal Tissue: It is Not as Fragile as It Seems. *Ann Vasc Surg* 72**,** 571-577. doi: 10.1016/j.avsg.2020.11.028.

Gomes, V.C., Gomes, J., Silvestre, G.C., Queiroz, A., Marques, M.A., and Silva, E.S.d. (2020). Biomechanics data of human supra-aortic trunks and abdominal visceral arteries harvested during autopsy. *Data in Brief* 33**,** 106569. doi: 10.1016/j.dib.2020.106569.

Grässel, D., Prescher, A., Fitzek, S., Keyserlingk, D.G., and Axer, H. (2005). Anisotropy of human linea alba: a biomechanical study. *J Surg Res* 124(1)**,** 118-125. doi: 10.1016/j.jss.2004.10.010.

Gremare, A., Jean-Gilles, S., Musqui, P., Magnan, L., Torres, Y., Fenelon, M., et al. (2019). Cartography of the mechanical properties of the human amniotic membrane. *Journal of the Mechanical Behavior of Biomedical Materials* 99**,** 18-26. doi: 10.1016/j.jmbbm.2019.07.007.

Grgić, I., Karakašić, M., Ivandić, Z., and Lulić, T.J. (2021). The Development of a Gracilis and Quadriceps Tendons Calibration Device for Uniaxial Tensile Tests. *Machines* 9(12), 364. doi: 10.3390/machines9120364.

Guinea, G.V., Atienza, J.M., Rojo, F.J., Garcia-Herrera, C.M., Li, Y.Q., Claes, E., et al. (2010). Factors influencing the mechanical behaviour of healthy human descending thoracic aorta. *Physiological Measurement* 31(12), 1553-1565. doi: 10.1088/0967-3334/31/12/001.

Gultova, E., Horny, L., Chlup, H., Zitny, R., Adamek, T., and Kulvajtova, M. (2013). Preservation of tangent modulus of pericardial tissue during cold storage. *Comput Methods Biomech Biomed Engin* 16 Suppl 1, 318-319. doi: 10.1080/10255842.2013.815932.

Guo, J.C., Liu, X.Y., Ding, X.L., Wang, L.Z., and Fan, Y.B. (2018). Biomechanical and mechanical behavior of the plantar fascia in macro and micro structures. *Journal of Biomechanics* 76, 160-166. doi: 10.1016/j.jbiomech.2018.05.032.

Hammer, N., Huster, D., Fritsch, S., Hadrich, C., Koch, H., Schmidt, P., et al. (2014). Do Cells Contribute to Tendon and Ligament Biomechanics? *Plos One* 9(8), 14. doi: 10.1371/journal.pone.0105037.

Hansen, P., Haraldsson, B.T., Aagaard, P., Kovanen, V., Avery, N.C., Qvortrup, K., et al. (2010). Lower strength of the human posterior patellar tendon seems unrelated to mature collagen cross-linking and fibril morphology. *J Appl Physiol (1985)* 108(1), 47-52. doi: 10.1152/japplphysiol.00944.2009.

Hanson, P., Aagaard, P., and Magnusson, S.P. (2012). Biomechanical properties of isolated fascicles of the Iliopsoas and Achilles tendons in African American and Caucasian men. *Ann Anat* 194(5), 457-460. doi: 10.1016/j.aanat.2012.03.007.

Hanuza, J., Maczka, M., Gasior-Glogowska, M., Komorowska, M., Kobielarz, M., Bedzinski, R., et al. (2010). FT-Raman spectroscopic study of thoracic aortic wall subjected to uniaxial stress. *Journal of Raman Spectroscopy* 41(10), 1163-1169. doi: 10.1002/jrs.2554.

Har-Shai, Y., Bodner, S.R., Egozy-Golan, D., Lindenbaum, E.S., Ben-Izhak, O., Mitz, V., et al. (1997). Viscoelastic properties of the superficial musculoaponeurotic system (SMAS): a microscopic and mechanical study. *Aesthetic Plast Surg* 21(4), 219-224. doi: 10.1007/s002669900113.

Haraldsson, B.T., Aagaard, P., Krogsgaard, M., Alkjaer, T., Kjaer, M., and Magnusson, S.P. (2005). Region-specific mechanical properties of the human patella tendon. *Journal of Applied Physiology* 98(3), 1006-1012. doi: 10.1152/japplphysiol.00482.2004.

Haraldsson, B.T., Aagaard, P., Qvortrup, K., Bojsen-Moller, J., Krogsgaard, M., Koskinen, S., et al. (2008). Lateral force transmission between human tendon fascicles. *Matrix Biology* 27(2), 86-95. doi: 10.1016/j.matbio.2007.09.001.

He, C.M., and Roach, M.R. (1994). The composition and mechanical properties of abdominal aortic aneurysms. *J Vasc Surg* 20(1)**,** 6-13. doi: 10.1016/0741-5214(94)90169-4.

He, X., Auricchio, F., Morganti, S., and Lu, J. (2021a). Uniaxial properties of ascending aortic aneurysms in light of effective stretch. *Acta Biomater* 136**,** 306-313. doi: 10.1016/j.actbio.2021.09.029.

He, Y., Guo, Y., Wang, J., Lv, W., Li, X., and Chen, K. (2021b). The posterior eye with age-related macular degeneration has isotropic and nonlinear viscoelastic properties. *J Mech Behav Biomed Mater* 114**,** 104207. doi: 10.1016/j.jmbbm.2020.104207.

Helmig, R., Oxlund, H., Petersen, L.K., and Uldbjerg, N. (1993). Different Biomechanical Properties of Human Fetal Membranes Obtained before and after Delivery. *European Journal of Obstetrics Gynecology and Reproductive Biology* 48(3)**,** 183-189. doi: 10.1016/0028-2243(93)90086-R.

Henderson, B.S., Cudworth, K.F., Wale, M.E., Siegel, D.N., and Lujan, T.J. (2022). Tensile fatigue strength and endurance limit of human meniscus. *Journal of the Mechanical Behavior of Biomedical Materials* 127**,** 10. doi: 10.1016/j.jmbbm.2021.105057.

Hoffman, A.S., Grande, L.A., and Park, J.B. (1977). Sequential enzymolysis of human aorta and resultant stress-strain behavior. *Biomater Med Devices Artif Organs* 5(2)**,** 121-145. doi: 10.3109/10731197709118669.

Hohmann, E., Keough, N., Glatt, V., Tetsworth, K., Putz, R., and Imhoff, A. (2019). The mechanical properties of fresh versus fresh/frozen and preserved (Thiel and Formalin) long head of biceps tendons: A cadaveric investigation. *Annals of Anatomy-Anatomischer Anzeiger* 221**,** 186-191. doi: 10.1016/j.aanat.2018.05.002.

Holzapfel, G.A., Sommer, G., Gasser, C.T., and Regitnig, P. (2005). Determination of layer-specific mechanical properties of human coronary arteries with nonatherosclerotic intimal thickening and related constitutive modeling. *American Journal of Physiology-Heart and Circulatory Physiology* 289(5)**,** H2048-H2058. doi: 10.1152/ajpheart.00934.2004.

Holzapfel, G.A., Sommer, G., and Regitnig, P. (2004). Anisotropic mechanical properties of tissue components in human atherosclerotic plaques. *J Biomech Eng* 126(5)**,** 657-665. doi: 10.1115/1.1800557.

Hou, X.H., Yuan, Y., Yin, J., Yang, S.B., Xie, L.L., and Wang, S.J. (2013). Fetal umbilical vein transplantation for the repair of middle cerebral artery injury. *Neural Regeneration Research* 8(34)**,** 3249-3254. doi: 10.3969/j.issn.1673-5374.2013.34.009.

Huang, C.Y., Stankiewicz, A., Ateshian, G.A., and Mow, V.C. (2005). Anisotropy, inhomogeneity, and tension-compression nonlinearity of human glenohumeral cartilage in finite deformation. *J Biomech* 38(4)**,** 799-809. doi: 10.1016/j.jbiomech.2004.05.006.

Huang, C.Y., Wang, V.M., Flatow, E.L., and Mow, V.C. (2009). Temperature-dependent viscoelastic properties of the human supraspinatus tendon. *Journal of Biomechanics* 42(4)**,** 546-549. doi: 10.1016/j.jbiomech.2008.11.013.

Iliopoulos, D.C., Deveja, R.P., Kritharis, E.P., Perrea, D., Sionis, G.D., Toutouzas, K., et al. (2009a). #Regional and directional variations in the mechanical properties of ascending thoracic aortic aneurysms. *Med Eng Phys* 31(1)**,** 1-9. doi: 10.1016/j.medengphy.2008.03.002.

Iliopoulos, D.C., Kritharis, E.P., Boussias, S., Demis, A., Iliopoulos, C.D., and Sokolis, D.P. (2013). #Biomechanical properties and histological structure of sinus of Valsalva aneurysms in relation to age and region. *J Biomech* 46(5)**,** 931-940. doi: 10.1016/j.jbiomech.2012.12.004.

Iliopoulos, D.C., Kritharis, E.P., Giagini, A.T., Papadodima, S.A., and Sokolis, D.P. (2009b). #Ascending thoracic aortic aneurysms are associated with compositional remodeling and vessel stiffening but not weakening in age-matched subjects. *Journal of Thoracic and Cardiovascular Surgery* 137(1)**,** 101-109. doi: 10.1016/j.jtcvs.2008.07.023.

Isaacs, J.L., Vresilovic, E., Sarkar, S., and Marcolongo, M. (2014). Role of biomolecules on annulus fibrosus micromechanics: Effect of enzymatic digestion on elastic and failure properties. *Journal of the Mechanical Behavior of Biomedical Materials* 40**,** 75-84. doi: 10.1016/j.jmbbm.2014.08.012.

Jabareen, M., Mallik, A.S., Bilic, G., Zisch, A.H., and Mazza, E. (2009). Relation between mechanical properties and microstructure of human fetal membranes: an attempt towards a quantitative analysis. *Eur J Obstet Gynecol Reprod Biol* 144 Suppl 1**,** S134-141. doi: 10.1016/j.ejogrb.2009.02.032.

Jacquet, C., Erivan, R., Sharma, A., Pithioux, M., Parratte, S., Argenson, J.-N., et al. (2019). Preservation Methods Influence the Biomechanical Properties of Human Lateral Menisci: An Ex Vivo Comparative Study of 3 Methods. *Orthop J Sports Med* 7(7)**,** 2325967119841622. doi: 10.1177/2325967119841622.

Jacquet, C., Jaubert, M., Pioger, C., Sbihi, A., Pithioux, M., Le Baron, M., et al. (2020). Presoaking of Semitendinosus Graft With Vancomycin Does Not Alter Its Biomechanical Properties: A Biomechanical In Vitro-Controlled Study Using Graft From Living Donors. *Arthroscopy-the Journal of Arthroscopic and Related Surgery* 36(8)**,** 2231-2236. doi: 10.1016/j.arthro.2020.03.037.

Jankowska, M.A., Bartkowiak-Jowsa, M., and Bedzinski, R. (2015). Experimental and constitutive modeling approaches for a study of biomechanical properties of human coronary arteries. *J Mech Behav Biomed Mater* 50**,** 1-12. doi: 10.1016/j.jmbbm.2015.05.021.

Jayyosi, C., Coret, M., and Bruyere-Garnier, K. (2013). Imaging of the human Glisson's capsule by two-photon excitation microscopy and mechanical characterisation by uniaxial tensile tests. *Computer Methods in Biomechanics and Biomedical Engineering* 16**,** 282-283. doi: 10.1080/10255842.2013.815869.

Kadlub, N., Debelmas, A., Dallard, J., Picard, A., and Boisson, J. (2020). Modeling of the human mandibular periosteum material properties and comparison with the calvarial periosteum. *Biomechanics and Modeling in Mechanobiology* 19(2)**,** 461-470. doi: 10.1007/s10237-019-01221-6.

Kang, H., Bao, G.J., and Qi, S.N. (2006). Biomechanical responses of human temporomandibular joint disc under tension and compression. *Int J Oral Maxillofac Surg* 35(9)**,** 817-821. doi: 10.1016/j.ijom.2006.03.005.

Karimi, A., Navidbakhsh, M., Alizadeh, M., and Shojaei, A. (2014). A comparative study on the mechanical properties of the umbilical vein and umbilical artery under uniaxial loading. *Australas Phys Eng Sci Med* 37**,** 645–654. doi: 10.1016/j.artres.2014.02.001.

Karimi, A., Navidbakhsh, M., and Kudo, S. (2015a). A comparative study on the mechanical properties of the healthy and varicose human saphenous vein under uniaxial loading. *J Med Eng Technol* 39(8)**,** 490-497. doi: 10.3109/03091902.2015.1086030.

Karimi, A., Navidbakhsh, M., and Shojaei, A. (2015b). A combination of histological analyses and uniaxial tensile tests to determine the material coefficients of the healthy and atherosclerotic human coronary arteries. *Tissue Cell* 47(2)**,** 152-158. doi: 10.1016/j.tice.2015.01.004.

Karimi, A., Navidbakhsh, M., Shojaei, A., and Faghihi, S. (2013). Measurement of the uniaxial mechanical properties of healthy and atherosclerotic human coronary arteries. *Materials Science & Engineering C-Materials for Biological Applications* 33(5)**,** 2550-2554. doi: 10.1016/j.msec.2013.02.016.

Karimi, A., and Razaghi, R. (2018). The role of smoking on the mechanical properties of the human lung. *Technol Health Care* 26(6)**,** 963-972. doi: 10.3233/THC-181340.

Karimi, A., Sera, T., Kudo, S., and Navidbakhsh, M. (2016). Experimental verification of the healthy and atherosclerotic coronary arteries incompressibility via Digital Image Correlation. *Artery Research* 16**,** 1-7. doi: 10.1016/j.artres.2016.08.002.

Karimi, A., and Shojaei, A. (2018). An Experimental Study to Measure the Mechanical Properties of the Human Liver. *Digestive Diseases* 36(2)**,** 150-155. doi: 10.1159/000481344.

Karimi, A., Shojaei, A., and Tehrani, P. (2017). Measurement of the mechanical properties of the human gallbladder. *J Med Eng Technol* 41(7)**,** 541-545. doi: 10.1080/03091902.2017.1366561.

Kempson, G.E. (1991). Age-related changes in the tensile properties of human articular cartilage: a comparative study between the femoral head of the hip joint and the talus of the ankle joint. *Biochim Biophys Acta* 1075(3)**,** 223-230. doi: 10.1016/0304-4165(91)90270-q.

Kempson, G.E., Freeman, M.A., and Swanson, S.A. (1968). Tensile properties of articular cartilage. *Nature* 220(5172)**,** 1127-1128. doi: 10.1038/2201127b0.

Kempson, G.E., Muir, H., Pollard, C., and Tuke, M. (1973). The tensile properties of the cartilage of human femoral condyles related to the content of collagen and glycosaminoglycans. *Biochim Biophys Acta* 297(2)**,** 456-472. doi: 10.1016/0304-4165(73)90093-7.

Khanafer, K., Duprey, A., Zainal, M., Schlicht, M., Williams, D., and Berguer, R. (2011). Determination of the elastic modulus of ascending thoracic aortic aneurysm at different ranges of pressure using uniaxial tensile testing. *J Thorac Cardiovasc Surg* 142(3)**,** 682-686. doi: 10.1016/j.jtcvs.2010.09.068.

Khanafer, K., Ghosh, A., and Vafai, K. (2019). Correlation between MMP and TIMP levels and elastic moduli of ascending thoracic aortic aneurysms. *Cardiovascular Revascularization Medicine* 20(4)**,** 324-327. doi: 10.1016/j.carrev.2018.07.006.

Khanafer, K., Schlicht, M.S., and Berguer, R. (2013). How should we measure and report elasticity in aortic tissue? *Eur J Vasc Endovasc Surg* 45(4)**,** 332-339. doi: 10.1016/j.ejvs.2012.12.015.

Kholinne, E., Kwak, J.M., Sun, Y., Koh, K.H., and Jeon, I.H. (2021). The forearm interosseous ligament: comparative mechanical properties of the proximal, central, and distal bands. *J Hand Surg Eur Vol* 46(2)**,** 184-187. doi: 10.1177/1753193420939497.

Kim, S.M., McCulloch, T., and Rim, K. (1998). Evaluation of the viscoelastic properties of pharyngeal tissue. *Tissue Eng* 4(4)**,** 389-401. doi: 10.1089/ten.1998.4.389.

Kirilova, M., Pashkouleva, D., and Kavardzhikov, V. (2013). The influence of gender on the elastic mechanical properties of human abdominal fascia. *Comptes Rendus De L Academie Bulgare Des Sciences* 66(6)**,** 871-876. doi: 10.7546/CR-2013-66-6-13101331-13.

Kirilova, M., Stoytchev, S., Pashkouleva, D., and Kavardzhikov, V. (2011). Experimental study of the mechanical properties of human abdominal fascia. *Medical Engineering & Physics* 33(1)**,** 1-6. doi: 10.1016/j.medengphy.2010.07.017.

Kirilova-Doneva, M., and Pashkouleva, D. (2022). The effects of age and sex on the elastic mechanical properties of human abdominal fascia. *Clinical Biomechanics* 92**,** 6. doi: 10.1016/j.clinbiomech.2022.105591.

Kirilova-Doneva, M., Pashkouleva, D., and Kavardzhikov, V. (2016). The effects of strain amplitude and localization on viscoelastic mechanical behaviour of human abdominal fascia. *Acta Bioeng Biomech* 18(4)**,** 127-133. doi: 10.5277/ABB-00358-2015-02.

Kirilova-Doneva, M., Pashkouleva, D., and Stoytchev, S. (2020). Age-related changes in mechanical properties of human abdominal fascia. *Medical & Biological Engineering & Computing* 58(7)**,** 1565-1573. doi: 10.1007/s11517-020-02172-2.

Kizmazoglu, C., Aydin, H.E., Kaya, I., Atar, M., Husemoglu, B., Kalemci, O., et al. (2019). Comparison of Biomechanical Properties of Dura Mater Substitutes and Cranial Human Dura Mater : An In Vitro Study. *J Korean Neurosurg Soc* 62 (6)**,** 635-642. doi: 10.3340/jkns.2019.0122.

Klinich, K.D., Miller, C.S., Hu, J.W., Nazmi, G.M., Pearlman, M.D., Schneider, L.W., et al. (2012). Effect of Frozen Storage on Dynamic Tensile Properties of Human Placenta. *Journal of Biomechanical Engineering-Transactions of the Asme* 134(3)**,** 034501. doi: 10.1115/1.4006025.

Kobielarz, M. (2020). Effect of collagen fibres and elastic lamellae content on the mechanical behaviour of abdominal aortic aneurysms. *Acta of Bioengineering and Biomechanics* 22(3)**,** 9-21. doi: 10.37190/abb-01580-2020-02.

Kobielarz, M., Chwilkowska, A., Turek, A., Maksymowicz, K., and Marciniak, M. (2015). Influence of selective digestion of elastin and collagen on mechanical properties of human aortas. *Acta of Bioengineering and Biomechanics* 17(2)**,** 55-62. doi: 10.5277/Abb-00184-2014-02.

Kobielarz, M., and Jankowski, L.J. (2013). Experimental Characterization of the Mechanical Properties of the Abdominal Aortic Aneurysm Wall under Uniaxial Tension. *Journal of Theoretical and Applied Mechanics* 51(4)**,** 949-958.

Kochova, P., Cimrman, R., Jansova, M., Michalova, K., Kalis, V., Kubikova, T., et al. (2019). The histological microstructure and in vitro mechanical properties of the human female postmenopausal perineal body. *Menopause-the Journal of the North American Menopause Society* 26(1)**,** 66-77. doi: 10.1097/gme.0000000000001166.

Koh, C.T., Tonsomboon, K., and Oyen, M.L. (2019). Fracture toughness of human amniotic membranes. *Interface Focus* 9**,** 20190012. doi: doi: 10.1098/rsfs.2019.0012.

Kolz, C.W., Suter, T., and Henninger, H.B. (2015). Regional mechanical properties of the long head of the biceps tendon. *Clin Biomech (Bristol, Avon)* 30(9)**,** 940-945. doi: 10.1016/j.clinbiomech.2015.07.005.

Komolafe, O.A., and Doehring, T.C. (2010). Fascicle-Scale Loading and Failure Behavior of the Achilles Tendon. *Journal of Biomechanical Engineering-Transactions of the Asme* 132(2)**,** 5. doi: 10.1115/1.4000696.

Kozun, M., Chwilkowska, A., Pezowicz, C., and Kobielarz, M. (2021). Influence of atherosclerosis on anisotropy and incompressibility of the human thoracic aortic wall. *Biocybernetics and Biomedical Engineering* 41(1)**,** 15-27. doi: 10.1016/j.bbe.2020.11.004.

Kozun, M., Plonek, T., Jasinski, M., and Filipiak, J. (2019). Effect of dissection on the mechanical properties of human ascending aorta and human ascending aorta aneurysm. *Acta of Bioengineering and Biomechanics* 21(2)**,** 127-134. doi: 10.5277/abb-01376-2019-01.

Kritharis, E.P., Iliopoulos, D.C., Papadodima, S.A., and Sokolis, D.P. (2014). Effects of aneurysm on the mechanical properties and histologic structure of aortic sinuses. *Ann Thorac Surg* 98(1)**,** 72-79. doi: 10.1016/j.athoracsur.2014.03.016.

Kumaraswamy, N., Khatam, H., Reece, G.P., Fingeret, M.C., Markey, M.K., and Ravi-Chandar, K. (2017). Mechanical response of human female breast skin under uniaxial stretching. *J Mech Behav Biomed Mater* 74**,** 164-175. doi: 10.1016/j.jmbbm.2017.05.027.

Kureshi, A., Vaiudea, P., Nazhat, S.N., Petrie, A., and Brown, R.A. (2008). Matrix mechanical properties of transversalis fascia in inguinal herniation as a model for tissue expansion. *Journal of Biomechanics* 41(16)**,** 3462-3468. doi: 10.1016/j.jbiomech.2008.08.018.

Lake, S.P., Cortes, D.H., Kadlowec, J.A., Soslowsky, L.J., and Elliott, D.M. (2012). Evaluation of affine fiber kinematics in human supraspinatus tendon using quantitative projection plot analysis. *Biomechanics and Modeling in Mechanobiology* 11(1-2), 197-205. doi: 10.1007/s10237-011-0303-5.

Lake, S.P., Miller, K.S., Elliott, D.M., and Soslowsky, L.J. (2009). Effect of Fiber Distribution and Realignment on the Nonlinear and Inhomogeneous Mechanical Properties of Human Supraspinatus Tendon under Longitudinal Tensile Loading. *Journal of Orthopaedic Research* 27(12), 1596-1602. doi: 10.1002/jor.20938.

Lake, S.P., Miller, K.S., Elliott, D.M., and Soslowsky, L.J. (2010). Tensile properties and fiber alignment of human supraspinatus tendon in the transverse direction demonstrate inhomogeneity, nonlinearity, and regional isotropy. *Journal of Biomechanics* 43(4), 727-732. doi: 10.1016/j.jbiomech.2009.10.017.

Lawlor, M.G., O'Donnell, M.R., O'Connell, B.M., and Walsh, M.T. (2011). Experimental determination of circumferential properties of fresh carotid artery plaques. *Journal of Biomechanics* 44(9), 1709-1715. doi: 10.1016/j.jbiomech.2011.03.033.

Lechner, K., Hull, M.L., and Howell, S.M. (2000). Is the circumferential tensile modulus within a human medial meniscus affected by the test sample location and cross-sectional area? *Journal of Orthopaedic Research* 18(6), 945-951. doi: 10.1002/jor.1100180614.

Lee, M.C., and Haut, R.C. (1989). Insensitivity of tensile failure properties of human bridging veins to strain rate: implications in biomechanics of subdural hematoma. *J Biomech* 22(6-7), 537-542. doi: 10.1016/0021-9290(89)90005-5.

Leng, X., Zhou, B., Deng, X., Davis, L., Sutton, M.A., Shazly, T., et al. (2018). Determination of Viscoelastic Properties of human Carotid Atherosclerotic Plaque by Inverse Boundary Value Analysis. *IOP Conf Ser Mater Sci Eng* 381. doi: 10.1088/1757-899X/381/1/012171.

Li, L., Qian, X., Yan, S., Lei, J., Wang, X., Zhang, H., et al. (2013). Determination of material parameters of the two-dimensional Holzapfel-Weizsacker type model based on uniaxial extension data of arterial walls. *Comput Methods Biomech Biomed Engin* 16(4), 358-367. doi: 10.1080/10255842.2011.621121.

Liao, X., Kemp, S., Corner, G., Eisma, R., and Huang, Z. (2015). Elastic properties of Thiel-embalmed human ankle tendon and ligament. *Clin Anat* 28(7), 917-924. doi: 10.1002/ca.22512.

Lisický, O., Hrubanová, A., Staffa, R., Vlachovský, R., and Burša, J. (2021). Constitutive models and failure properties of fibrous tissues of carotid artery atheroma based on their uniaxial testing. *J Biomech* 129, 110861. doi: 10.1016/j.jbiomech.2021.110861.

Liu, T., Shen, M., Huang, L., Xiang, Y., Li, H., Zhang, Y., et al. (2020a). Characterization of hyperelastic mechanical properties for youth corneal anterior central stroma based on collagen fibril crimping constitutive model. *J Mech Behav Biomed Mater* 103, 103575. doi: 10.1016/j.jmbbm.2019.103575.

Liu, T.W., Shen, M., Li, H.X., Zhang, Y., Mu, B.K., Zhao, X.H., et al. (2020b). Changes and quantitative characterization of hyper-viscoelastic biomechanical properties for young corneal stroma after standard corneal cross-linking treatment with different ultraviolet-A energies. *Acta Biomaterialia* 113**,** 438-451. doi: 10.1016/j.actbio.2020.06.005.

Liu, X., Cleary, J., and German, G.K. (2016). The global mechanical properties and multi-scale failure mechanics of heterogeneous human stratum corneum. *Acta Biomaterialia* 43**,** 78-87. doi: 10.1016/j.actbio.2016.07.028.

Lopez, S.O., Eberhart, R.C., Zimmern, P.E., and Chuong, C.J. (2015). Influence of body mass index on the biomechanical properties of the human prolapsed anterior vaginal wall. *International Urogynecology Journal* 26(4)**,** 519-525. doi: 10.1007/s00192-014-2525-4.

Loree, H.M., Grodzinsky, A.J., Park, S.Y., Gibson, L.J., and Lee, R.T. (1994). Static circumferential tangential modulus of human atherosclerotic tissue. *J Biomech* 27(2)**,** 195-204. doi: 10.1016/0021-9290(94)90209-7.

Lozano, P.F., Scholze, M., Babian, C., Scheidt, H., Vielmuth, F., Waschke, J., et al. (2019). Water-content related alterations in macro and micro scale tendon biomechanics. *Scientific Reports* 9**,** 12. doi: 10.1038/s41598-019-44306-z.

Lujan, T.J., Underwood, C.J., Henninger, H.B., Thompson, B.M., and Weiss, J.A. (2007). Effect of dermatan sulfate glycosaminoglycans on the quasi-static material properties of the human medial collateral ligament. *J Orthop Res* 25(7)**,** 894-903. doi: 10.1002/jor.20351.

Lyu, S.R., Tzeng, J.E., Kuo, C.Y., Jian, A.R., and Liu, D.S. (2006). Mechanical strength of mediopatellar plica--the influence of its fiber content. *Clin Biomech (Bristol, Avon)* 21(8)**,** 860-863. doi: 10.1016/j.clinbiomech.2006.03.010.

Ma, J.L., Wu, F.Y., Liu, Z.Y., Fang, Y.J., Chu, X., Zheng, L.Y., et al. (2022). Biomechanical Considerations of Patching Material for Posterior Scleral Reinforcement Surgery. *Frontiers in Medicine* 9**,** 10. doi: 10.3389/fmed.2022.888542.

Ma, Z., Hu, S., Tan, J.S., Myer, C., Njus, N.M., and Xia, Z. (2013). In vitro and in vivo mechanical properties of human ulnar and median nerves. *J Biomed Mater Res A* 101(9)**,** 2718-2725. doi: 10.1002/jbm.a.34573.

Maher, E., Creane, A., Sultan, S., Hynes, N., Lally, C., and Kelly, D.J. (2009). Tensile and compressive properties of fresh human carotid atherosclerotic plaques. *Journal of Biomechanics* 42(16)**,** 2760-2767. doi: 10.1016/j.jbiomech.2009.07.032.

Manoogian, S.J., Bisplinghoff, J.A., McNally, C., Kemper, A.R., Santago, A.C., and Duma, S.M. (2008). Dynamic tensile properties of human placenta. *J Biomech* 41**,** 3436-3440. doi: 10.1016/j.jbiomech.2008.09.020.

Manoogian, S.J., Bisplinghoff, J.A., McNally, C., Kemper, A.R., Santago, A.C., and Duma, S.M. (2009). Effect of Strain Rate on the Tensile Material Properties of Human Placenta. *Journal of Biomechanical Engineering-Transactions of the Asme* 131(9)**,** 6. doi: 10.1115/1.3194694.

Manopoulos, C., Karathanasis, I., Kouerinis, I., Angouras, D.C., Lazaris, A., Tsangaris, S., et al. (2018). Identification of regional/layer differences in failure properties and thickness as important biomechanical factors responsible for the initiation of aortic dissections. *J Biomech* 80**,** 102-110. doi: 10.1016/j.jbiomech.2018.08.024.

Martins, P., Lopes Silva-Filho, A., Rodrigues Maciel da Fonseca, A.M., Santos, A., Santos, L., Mascarenhas, T., et al. (2013a). Biomechanical properties of vaginal tissue in women with pelvic organ prolapse. *Gynecol Obstet Invest* 75(2)**,** 85-92. doi: 10.1159/000343230.

Martins, P., Pena, E., Calvo, B., Doblare, M., Mascarenhas, T., Natal Jorge, R., et al. (2010). Prediction of nonlinear elastic behaviour of vaginal tissue: experimental results and model formulation. *Comput Methods Biomech Biomed Engin* 13(3)**,** 327-337. doi: 10.1080/10255840903208197.

Martins, P., Pena, E., Jorge, R.M., Santos, A., Santos, L., Mascarenhas, T., et al. (2012). Mechanical characterization and constitutive modelling of the damage process in rectus sheath. *J Mech Behav Biomed Mater* 8**,** 111-122. doi: 10.1016/j.jmbbm.2011.12.005.

Martins, P., Silva-Filho, A.L., Fonseca, A.M., Santos, A., Santos, L., Mascarenhas, T., et al. (2013b). Strength of round and uterosacral ligaments: a biomechanical study. *Arch Gynecol Obstet* 287(2)**,** 313-318. doi: 10.1007/s00404-012-2564-3.

Martins, P.A., Filho, A.L., Fonseca, A.M., Santos, A., Santos, L., Mascarenhas, T., et al. (2011a). Uniaxial mechanical behavior of the human female bladder. *Int Urogynecol J* 22(8)**,** 991-995. doi: 10.1007/s00192-011-1409-0.

Martins, P.A.L.S., Jorge, R.M.N., Ferreia, A.J.M., Saleme, C.S., Roza, T., Parente, M.M.P., et al. (2011b). Vaginal Tissue Properties versus Increased Intra-Abdominal Pressure: A Preliminary Biomechanical Study. *Gynecologic and Obstetric Investigation* 71(3)**,** 145-150. doi: 10.1159/000315160.

Martufi, G., Satriano, A., Moore, R.D., Vorp, D.A., and Di Martino, E.S. (2015). Local Quantification of Wall Thickness and Intraluminal Thrombus Offer Insight into the Mechanical Properties of the Aneurysmal Aorta. *Annals of Biomedical Engineering* 43(8)**,** 1759-1771. doi: 10.1007/s10439-014-1222-2.

Massalou, D., Masson, C., Afquir, S., Baque, P., Arnoux, P.J., and Bege, T. (2019a). Influence of gender, age, shelf-life, and conservation method on the biomechanical behavior of colon tissue under dynamic solicitation. *Clinical Biomechanics* 65**,** 34-40. doi: 10.1016/j.clinbiomech.2019.03.017.

Massalou, D., Masson, C., Afquir, S., Baque, P., Arnoux, P.J., and Bege, T. (2019b). Mechanical effects of load speed on the human colon. *J Biomech* 91**,** 102-108. doi: 10.1016/j.jbiomech.2019.05.012.

Massalou, D., Masson, C., Foti, P., Afquir, S., Baque, P., Berdah, S.V., et al. (2016). Dynamic biomechanical characterization of colon tissue according to anatomical factors. *Journal of Biomechanics* 49(16), 3861-3867. doi: 10.1016/j.jbiomech.2016.10.023.

Mauri, A., Perrini, M., Ehret, A.E., De Focatiis, D.S., and Mazza, E. (2015). Time-dependent mechanical behavior of human amnion: macroscopic and microscopic characterization. *Acta Biomater* 11, 314-323. doi: 10.1016/j.actbio.2014.09.012.

Metschl, S., Bruder, L., Paloschi, V., Jakob, K., Reutersberg, B., Reeps, C., et al. (2022). Changes in Endocan and Dermatan Sulfate Are Associated with Biomechanical Properties of Abdominal Aortic Wall during Aneurysm Expansion and Rupture. *Thromb Haemost* 122(9), 1513-1523. doi: 10.1055/a-1772-0574.

Mohan, D., and Melvin, J.W. (1982). Failure properties of passive human aortic tissue.1. Uniaxial tension tests. *Journal of Biomechanics* 15(11), 887-&. doi: 10.1016/0021-9290(82)90055-0.

Monea, A.G., Baeck, K., Verbeken, E., Verpoest, I., Sloten, J.V., Goffin, J., et al. (2014). The biomechanical behaviour of the bridging vein-superior sagittal sinus complex with implications for the mechanopathology of acute subdural haematoma. *J Mech Behav Biomed Mater* 32, 155-165. doi: 10.1016/j.jmbbm.2013.12.007.

Monson, K.L., Goldsmith, W., Barbaro, N.M., and Manley, G.T. (2003). Axial mechanical properties of fresh human cerebral blood vessels. *Journal of Biomechanical Engineering-Transactions of the Asme* 125(2), 288-294. doi: 10.1115/1.1554412.

Moore, S.M., McMahon, P.J., Azemi, E., and Debski, R.E. (2005). Bi-directional mechanical properties of the posterior region of the glenohumeral capsule. *J Biomech* 38(6), 1365-1369. doi: 10.1016/j.jbiomech.2004.06.005.

Moore, S.M., McMahon, P.J., and Debski, R.E. (2004). Bi-directional mechanical properties of the axillary pouch of the glenohumeral capsule: implications for modeling and surgical repair. *Journal of Biomechanical Engineering-Transactions of the Asme* 126(2), 284-288. doi: 10.1115/1.1695574.

Morales-Orcajo, E., Becerro de Bengoa Vallejo, R., Losa Iglesias, M., and Bayod, J. (2016). Structural and material properties of human foot tendons. *Clin Biomech (Bristol, Avon)* 37, 1-6. doi: 10.1016/j.clinbiomech.2016.05.014.

Myers, K.M., Socrate, S., Paskaleva, A., and House, M. (2010). A study of the anisotropy and tension/compression behavior of human cervical tissue. *J Biomech Eng* 132(2), 021003. doi: 10.1115/1.3197847.

Nagle, A.S., Barker, M.A., Kleeman, S.D., Haridas, B., and Mast, T.D. (2014). Passive biomechanical properties of human cadaveric levator ani muscle at low strains. *Journal of Biomechanics* 47(2), 583-586. doi: 10.1016/j.jbiomech.2013.11.033.

Nesbitt, D.Q., Siegel, D.N., Nelson, S.J., and Lujan, T.J. (2021). Effect of age on the failure properties of human meniscus: High-speed strain mapping of tissue tears. *J Biomech* 115, 110126. doi: 10.1016/j.jbiomech.2020.110126.

Nguyen, B.A., Reilly, M.A., and Roberts, C.J. (2020). Biomechanical contribution of the sclera to dynamic corneal response in air-puff induced deformation in human donor eyes. *Experimental Eye Research* 191, 5. doi: 10.1016/j.exer.2019.107904.

Ní Annaidh, A., Bruyère, K., Destrade, M., Gilchrist, M.D., and Otténio, M. (2012). Characterization of the anisotropic mechanical properties of excised human skin. *J Mech Behav Biomed Mater* 5(1), 139-148. doi: 10.1016/j.jmbbm.2011.08.016.

Ninomiya, O.H., Tavares Monteiro, J.A., Higuchi Mde, L., Puech-Leao, P., de Luccia, N., Raghavan, M.L., et al. (2015). Biomechanical Properties and Microstructural Analysis of the Human Nonaneurysmal Aorta as a Function of Age, Gender and Location: An Autopsy Study. *J Vasc Res* 52(4), 257-264. doi: 10.1159/000442979.

Novotny, R., Mericka, P., Chlupac, J., Matejka, R., Kristek, J., Marada, T., et al. (2020). The Effect of Different Thawing Rates on Cryopreserved Human Iliac Arteries Allograft's Structural Damage and Mechanical Properties. *Biomed Research International* 2020. doi: 10.1155/2020/6545190.

O'Connell, G.D., Guerin, H.L., and Elliott, D.M. (2009). Theoretical and uniaxial experimental evaluation of human annulus fibrosus degeneration. *J Biomech Eng* 131(11), 111007. doi: 10.1115/1.3212104.

O'Leary, S.A., Mulvihill, J.J., Barrett, H.E., Kavanagh, E.G., Walsh, M.T., McGloughlin, T.M., et al. (2015). Determining the influence of calcification on the failure properties of abdominal aortic aneurysm (AAA) tissue. *J Mech Behav Biomed Mater* 42, 154-167. doi: 10.1016/j.jmbbm.2014.11.005.

Ollivier, M., Sbihi, J., Sbihi, A., Pithioux, M., Parratte, S., and Argenson, J.N. (2017). Ropivacaine alters the mechanical properties of hamstring tendons: In vitro controlled mechanical testing of tendons from living donors. *Orthop Traumatol Surg Res* 103(7), 1027-1030. doi: 10.1016/j.otsr.2017.05.024.

Otsuka, S., Yakura, T., Ohmichi, Y., Ohmichi, M., Naito, M., Nakano, T., et al. (2018). Site specificity of mechanical and structural properties of human fascia lata and their gender differences: A cadaveric study. *J Biomech* 77, 69-75. doi: 10.1016/j.jbiomech.2018.06.018.

Ottenio, M., Tran, D., Ni Annaidh, A., Gilchrist, M.D., and Bruyere, K. (2015). Strain rate and anisotropy effects on the tensile failure characteristics of human skin. *J Mech Behav Biomed Mater* 41, 241-250. doi: 10.1016/j.jmbbm.2014.10.006.

Oxlund, B.S., Ørtoft, G., Brüel, A., Danielsen, C.C., Bor, P., Oxlund, H., et al. (2010a). Collagen concentration and biomechanical properties of samples from the lower uterine cervix in relation to age and parity in non-pregnant women. *Reprod Biol Endocrinol* 8, 82. doi: 10.1186/1477-7827-8-82.

Oxlund, B.S., Ørtoft, G., Brüel, A., Danielsen, C.C., Oxlund, H., and Uldbjerg, N. (2010b). Cervical collagen and biomechanical strength in non-pregnant women with a history of cervical insufficiency. *Reprod Biol Endocrinol* 8**,** 92. doi: 10.1186/1477-7827-8-92.

Oxlund, H., Helmig, R., Halaburt, J.T., and Uldbjerg, N. (1990). Biomechanical Analysis of Human Chorioamniotic Membranes. *European Journal of Obstetrics Gynecology and Reproductive Biology* 34(3)**,** 247-255. doi: 10.1016/0028-2243(90)90078-f.

Oyen, M.L., Calvin, S.E., and Cook, R.F. (2004). Uniaxial stress-relaxation and stress-strain responses of human amnion. *Journal of Materials Science-Materials in Medicine* 15(5)**,** 619-624. doi: 10.1023/B:JMSM.0000026102.85071.1f.

Oyen, M.L., Cook, R.F., Stylianopoulos, T., Barocas, V.H., Calvin, S.E., and Landers, D.V. (2005). Uniaxial and biaxial mechanical behavior of human amnion. *Journal of Materials Research* 20(11)**,** 2902-2909. doi: Doi 10.1557/Jmr.2005.0382.

Paritala, P.K., Yarlagadda, P.K.D.V., Kansky, R., Wang, J., Mendieta, J.B., Gu, Y., et al. (2020). Stress-Relaxation and Cyclic Behavior of Human Carotid Plaque Tissue. *Front. Bioeng. Biotechnol.* 8.

Park, J., Shin, A., Jafari, S., and Demer, J.L. (2021). Material properties and effect of preconditioning of human sclera, optic nerve, and optic nerve sheath. *Biomechanics and Modeling in Mechanobiology* 20(4)**,** 1353-1363. doi: 10.1007/s10237-021-01448-2.

Pavan, P.G., Stecco, C., Darwish, S., Natali, A.N., and De Caro, R. (2011). Investigation of the mechanical properties of the plantar aponeurosis. *Surgical and Radiologic Anatomy* 33(10)**,** 905-911. doi: 10.1007/s00276-011-0873-z.

Pena, E., Martins, P., Mascarenhas, T., Natal Jorge, R.M., Ferreira, A., Doblare, M., et al. (2011). Mechanical characterization of the softening behavior of human vaginal tissue. *J Mech Behav Biomed Mater* 4(3)**,** 275-283. doi: 10.1016/j.jmbbm.2010.10.006.

Pennati, G. (2001). Biomechanical properties of the human umbilical cord. *Biorheology* 38(5-6)**,** 355-366.

Pfaeffle, H.J., Tomaino, M.M., Grewal, R., Xu, J., Boardman, N.D., Woo, S.L., et al. (1996). Tensile properties of the interosseous membrane of the human forearm. *J Orthop Res* 14(5)**,** 842-845. doi: 10.1002/jor.1100140525.

Pham, D.T., Shapter, J.G., and Costi, J.J. (2018). Tensile behaviour of individual fibre bundles in the human lumbar anulus fibrosus. *Journal of Biomechanics* 67**,** 24-31. doi: 10.1016/j.jbiomech.2017.11.016.

Pichamuthu, J.E., Phillippi, J.A., Cleary, D.A., Chew, D.W., Hempel, J., Vorp, D.A., et al. (2013). Differential Tensile Strength and Collagen Composition in Ascending Aortic Aneurysms by Aortic Valve Phenotype. *Annals of Thoracic Surgery* 96(6)**,** 2147-2154. doi: 10.1016/j.athoracsur.2013.07.001.

Pierce, D.M., Maier, F., Weisbecker, H., Viertler, C., Verbrugghe, P., Famaey, N., et al. (2015). Human thoracic and abdominal aortic aneurysmal tissues: Damage experiments, statistical analysis and constitutive modeling. *Journal of the Mechanical Behavior of Biomedical Materials* 41**,** 92-107. doi: 10.1016/j.jmbbm.2014.10.003.

Polzer, S., Man, V., Vlachovský, R., Kubíček, L., Kracík, J., Staffa, R., et al. (2021). Failure properties of abdominal aortic aneurysm tissue are orientation dependent. *J Mech Behav Biomed Mater* 114**,** 104181. doi: 10.1016/j.jmbbm.2020.104181.

Qian, K., Traylor, K., Lee, S.W., Ellis, B., Weiss, J., and Kamper, D. (2014). Mechanical properties vary for different regions of the finger extensor apparatus. *Journal of Biomechanics* 47(12)**,** 3094-3099. doi: 10.1016/j.jbiomech.2014.06.035.

Quapp, K.M., and Weiss, J.A. (1998). Material characterization of human medial collateral ligament. *Journal of Biomechanical Engineering-Transactions of the Asme* 120(6)**,** 757-763. doi: 10.1115/1.2834890.

Raghavan, M.L., Hanaoka, M.M., Kratzberg, J.A., de Lourdes Higuchi, M., and da Silva, E.S. (2011). Biomechanical failure properties and microstructural content of ruptured and unruptured abdominal aortic aneurysms. *J Biomech* 44(13)**,** 2501-2507. doi: 10.1016/j.jbiomech.2011.06.004.

Raghavan, M.L., Kratzberg, J., Castro de Tolosa, E.M., Hanaoka, M.M., Walker, P., and da Silva, E.S. (2006). Regional distribution of wall thickness and failure properties of human abdominal aortic aneurysm. *J Biomech* 39(16)**,** 3010-3016. doi: 10.1016/j.jbiomech.2005.10.021.

Raghavan, M.L., Webster, M.W., and Vorp, D.A. (1996). Ex vivo biomechanical behavior of abdominal aortic aneurysm: assessment using a new mathematical model. *Ann Biomed Eng* 24(5)**,** 573-582. doi: 10.1007/BF02684226.

Rainis, C.A., Brown, A.J., McMahon, P.J., and Debski, R.E. (2012). Effects of simulated injury on the anteroinferior glenohumeral capsule. *Medical & Biological Engineering & Computing* 50(12)**,** 1299-1307. doi: 10.1007/s11517-012-0961-1.

Rains, J.K., Bert, J.L., Roberts, C.R., and Paré, P.D. (1992). Mechanical properties of human tracheal cartilage. *J Appl Physiol (1985)* 72(1)**,** 219-225. doi: 10.1152/jappl.1992.72.1.219.

Rassoli, A., Fatouraee, N., and Shafigh, M. (2015). Uniaxial and biaxial mechanical properties of the human saphenous vein. *Biomedical Engineering-Applications Basis Communications* 27(5). doi: 10.4015/S1016237215500507.

Reeps, C., Maier, A., Pelisek, J., Härtl, F., Grabher-Meier, V., Wall, W.A., et al. (2013). Measuring and modeling patient-specific distributions of material properties in abdominal aortic aneurysm wall. *Biomech Model Mechanobiol* 12(4)**,** 717-733. doi: 10.1007/s10237-012-0436-1.

Richmon, J.D., Sage, A.B., Wong, V.W., Chen, A.C., Pan, C., Sah, R.L., et al. (2005). Tensile biomechanical properties of human nasal septal cartilage. *American Journal of Rhinology* 19(6)**,** 617-622. doi: 10.1177/194589240501900616.

Rivaux, G., Rubod, C., Dedet, B., Brieu, M., Gabriel, B., and Cosson, M. (2013). Comparative analysis of pelvic ligaments: a biomechanics study. *International Urogynecology Journal* 24(1)**,** 135-139. doi: 10.1007/s00192-012-1861-5.

Roberts, C.R., Rains, J.K., Paré, P.D., Walker, D.C., Wiggs, B., and Bert, J.L. (1997). Ultrastructure and tensile properties of human tracheal cartilage. *J Biomech* 81**,** 6.

Rotta, G., Kobiela, J., Grymek, S., and Karczewska, M. (2019). Mechanical properties of the human stomach under uniaxial stress action. *Current Science* 116(11)**,** 1886-1893. doi: 10.18520/cs/v116/i11/1886-1893.

Rubod, C., Boukerrou, M., Brieu, M., Clay, J.-C., Dubois, P., and Cosson, M. (2008). Biomechanical properties of vaginal tissue: preliminary results. *Int Urogynecol J* 19**,** 811–816. doi: 10.1007/s00192-007-0533-3.

Rubod, C., Brieu, M., Cosson, M., Rivaux, G., Clay, J.-C., De Landsheere, L., et al. (2012). Biomechanical Properties of Human Pelvic Organs. *Urology* 79(4)**,** 968.e917-922. doi: 10.1016/j.urology.2011.11.010.

Sacks, M.S., Jimenez Hamann, M.C., Otano-Lata, S.E., and Malinin, T.I. (1998). Local mechanical anisotropy in human cranial dura mater allografts. *J Biomech Eng* 120(4)**,** 541-544. doi: 10.1115/1.2798027.

Safshekan, F., Tafazzoli-Shadpour, M., Abdouss, M., and Shadmehr, M.B. (2016). Mechanical Characterization and Constitutive Modeling of Human Trachea: Age and Gender Dependency. *Materials (Basel)* 9(6). doi: 10.3390/ma9060456.

Sang, C., Maiti, S., Fortunato, R.N., Kofler, J., and Robertson, A.M. (2018). A Uniaxial Testing Approach for Consistent Failure in Vascular Tissues. *J Biomech Eng* 140(6)**,** 0610101-06101010. doi: 10.1115/1.4039577.

Sassani, S.G., Kakisis, J., Tsangaris, S., and Sokolis, D.P. (2015). Layer-dependent wall properties of abdominal aortic aneurysms: Experimental study and material characterization. *J Mech Behav Biomed Mater* 49**,** 141-161. doi: 10.1016/j.jmbbm.2015.04.027.

Schechtman, H., and Bader, D.L. (1997). In vitro fatigue of human tendons. *Journal of Biomechanics* 30(8)**,** 829-835. doi: 10.1016/s0021-9290(97)00033-x.

Schechtman, H., and Bader, D.L. (2002). Fatigue damage of human tendons. *J Biomech* 35(3)**,** 347-353. doi: 10.1016/s0021-9290(01)00177-4.

Schleifenbaum, S., Prietzel, T., Hadrich, C., Mobius, R., Sichting, F., and Hammer, N. (2016a). Tensile properties of the hip joint ligaments are largely variable and age-dependent - An in-vitro analysis in an age range of 14-93 years. *Journal of Biomechanics* 49(14)**,** 3437-3443. doi: 10.1016/j.jbiomech.2016.09.001.

Schleifenbaum, S., Schmidt, M., Möbius, R., Wolfskämpf, T., Schröder, C., Grunert, R., et al. (2016b). Load and failure behavior of human muscle samples in the context of proximal femur replacement. *BMC Musculoskeletal Disorders* 17**,** 149. doi: 10.1186/s12891-016-0998-7.

Schmid, F., Sommer, G., Rappolt, M., Schulze-Bauer, C.A., Regitnig, P., Holzapfel, G.A., et al. (2005). In situ tensile testing of human aortas by time-resolved small-angle X-ray scattering. *J Synchrotron Radiat* 12(Pt 6)**,** 727-733. doi: 10.1107/S0909049505012549.

Scholze, M., Safavi, S., Ramezani, M., Ondruschka, B., and Hammer, N. (2022). Fatigue Testing of Human Flexor Tendons Using a Customized 3D-Printed Clamping System. *Applied Sciences-Basel* 12(15). doi: 10.3390/app12157836.

Scholze, M., Singh, A., Lozano, P.F., Ondruschka, B., Ramezani, M., Werner, M., et al. (2018). Utilization of 3D printing technology to facilitate and standardize soft tissue testing. *Scientific Reports* 8**,** 13. doi: 10.1038/s41598-018-29583-4.

Schriefl, A.J., Schmidt, T., Balzani, D., Sommer, G., and Holzapfel, G.A. (2015). Selective enzymatic removal of elastin and collagen from human abdominal aortas: Uniaxial mechanical response and constitutive modeling. *Acta Biomaterialia* 17**,** 125-136. doi: 10.1016/j.actbio.2015.01.003.

Seehra, G.P., and Silver, F.H. (2006). Viscoelastic properties of acid- and alkaline-treated human dermis: a correlation between total surface charge and elastic modulus. *Skin Res Technol* 12(3)**,** 190-198. doi: 10.1111/j.0909-752X.2006.00150.x.

Shan, X., Otsuka, S., Yakura, T., Naito, M., Nakano, T., and Kawakami, Y. (2019). Morphological and mechanical properties of the human triceps surae aponeuroses taken from elderly cadavers: Implications for muscle-tendon interactions. *PLoS One* 14(2)**,** e0211485. doi: 10.1371/journal.pone.0211485.

Sherebrin, M.H., Hegney, J.E., and Roach, M.R. (1989). Effects of age on the anisotropy of the descending human thoracic aorta determined by uniaxial tensile testing and digestion by NaOH under load. *Can J Physiol Pharmacol* 67(8)**,** 871-878. doi: 10.1139/y89-136.

Sherifova, S., Sommer, G., Viertler, C., Regitnig, P., Caranasos, T., Smith, M., et al. (2019). Failure properties and microstructure of healthy and aneurysmatic human thoracic aortas subjected to uniaxial extension with a focus on the media. *Acta Biomaterialia* 99**,** 443-456. doi: 10.1016/j.actbio.2019.08.038.

Shin, A., Park, J., Le, A., Poukens, V., and Demer, J.L. (2020). Bilaminar Mechanics of the Human Optic Nerve Sheath. *Curr Eye Res* 45(7)**,** 854-863. doi: 10.1080/02713683.2019.1701689.

Signorelli, F., Pailler-Mattei, C., Gory, B., Larquet, P., Robinson, P., Vargiolu, R., et al. (2018). Biomechanical Characterization of Intracranial Aneurysm Wall: A Multiscale Study. *World Neurosurgery* 119**,** E882-E889. doi: 10.1016/j.wneu.2018.07.290.

Skaggs, D.L., Weidenbaum, M., Iatridis, J.C., Ratcliffe, A., and Mow, V.C. (1994). Regional variation in tensile properties and biochemical-composition of the human lumbar anulus fibrosus. *Spine* 19(12)**,** 1310-1319. doi: 10.1097/00007632-199406000-00002.

Skelley, N.W., Castile, R.M., Cannon, P.C., Weber, C.I., Brophy, R.H., and Lake, S.P. (2016). Regional Variation in the Mechanical and Microstructural Properties of the Human Anterior Cruciate Ligament. *American Journal of Sports Medicine* 44(11)**,** 2892-2899. doi: 10.1177/0363546516654480.

Skelley, N.W., Castile, R.M., York, T.E., Gruev, V., Lake, S.P., and Brophy, R.H. (2015). Differences in the microstructural properties of the anteromedial and posterolateral bundles of the anterior cruciate ligament. *Am J Sports Med* 43(4)**,** 928-936. doi: 10.1177/0363546514566192.

Smeets, K., Bellemans, J., Scheys, L., Eijnde, B.O., Slane, J., and Claes, S. (2017a). Mechanical Analysis of Extra-Articular Knee Ligaments. Part two: Tendon grafts used for knee ligament reconstruction. *Knee* 24(5)**,** 957-964. doi: 10.1016/j.knee.2017.07.011.

Smeets, K., Slane, J., Scheys, L., Claes, S., and Bellemans, J. (2017b). Mechanical Analysis of Extra-Articular Knee Ligaments. Part One: Native knee ligaments. *Knee* 24(5)**,** 949-956. doi: 10.1016/j.knee.2017.07.013.

Smeets, K., Slane, J., Scheys, L., Forsyth, R., Claes, S., and Bellemans, J. (2017c). The Anterolateral Ligament Has Similar Biomechanical and Histologic Properties to the Inferior Glenohumeral Ligament. *Arthroscopy-the Journal of Arthroscopic and Related Surgery* 33(5)**,** 9. doi: 10.1016/j.arthro.2017.01.038.

Smith, C.D., Masouros, S.D., Hill, A.M., Wallace, A.L., Amis, A.A., and Bull, A.M. (2008). Tensile properties of the human glenoid labrum. *J Anat* 212(1)**,** 49-54. doi: 10.1111/j.1469-7580.2007.00832.x.

Smith, M.V., Castile, R.M., Brophy, R.H., Dewan, A., Bernholt, D., and Lake, S.P. (2019). Mechanical Properties and Microstructural Collagen Alignment of the Ulnar Collateral Ligament During Dynamic Loading. *American Journal of Sports Medicine* 47(1)**,** 151-157. doi: 10.1177/0363546518812416.

Smoljkic, M., Fehervary, H., Van den Bergh, P., Jorge Peñas, A., Kluyskens, L., Dymarkowski, S., et al. (2017). Biomechanical characterization of ascending aortic aneurysms. *Biomech Model Mechanobiol* 16(2)**,** 705-720. doi: doi: 10.1007/s10237-016-0848-4.

Sokolis, D.P. (2019). In vitro study of age-related changes in human ureteral failure properties according to region, direction, and layer. *Proceedings of the Institution of Mechanical Engineers Part H-Journal of Engineering in Medicine* 233(5)**,** 570-583. doi: 10.1177/0954411919839891.

Sokolis, D.P., and Angouras, D.C. (2021). Failure properties of ascending thoracic aortic aneurysms with dysfunctional tricuspid aortic valves. *Interact Cardiovasc Thorac Surg* 33(6)**,** 949-958. doi: 10.1093/icvts/ivab184.

Sokolis, D.P., and Iliopoulos, D.C. (2014). Impaired mechanics and matrix metalloproteinases/inhibitors expression in female ascending thoracic aortic aneurysms. *Journal of the Mechanical Behavior of Biomedical Materials* 34**,** 154-164. doi: 10.1016/j.jmbbm.2014.02.015.

Sokolis, D.P., Kritharis, E.P., Giagini, A.T., Lampropoulos, K.M., Papadodima, S.A., and Iliopoulos, D.C. (2012a). Biomechanical response of ascending thoracic aortic aneurysms: association with structural remodelling. *Computer Methods in Biomechanics and Biomedical Engineering* 15(3)**,** 231-248. doi: 10.1080/10255842.2010.522186.

Sokolis, D.P., Kritharis, E.P., and Iliopoulos, D.C. (2012b). Effect of layer heterogeneity on the biomechanical properties of ascending thoracic aortic aneurysms. *Medical & Biological Engineering & Computing* 50(12)**,** 1227-1237. doi: 10.1007/s11517-012-0949-x.

Song, Y., Wu, D., Shen, M., Wang, L.K., Wang, C.Z., Cai, Y., et al. (2022). Measuring Human Corneal Stromal Biomechanical Properties Using Tensile Testing Combined With Optical Coherence Tomography. *Frontiers in Bioengineering and Biotechnology* 10**,** 9. doi: 10.3389/fbioe.2022.882392.

Stabile, K.J., Pfaeffle, J., Weiss, J.A., Fischer, K., and Tomaino, M.M. (2004). Bi-directional mechanical properties of the human forearm interosseous ligament. *Journal of Orthopaedic Research* 22(3)**,** 607-612. doi: 10.1016/j.orthres.2003.05.002.

Standardization, I.O.f. (2012). "ISO 527-2:2012 Plastics — Determination of tensile properties — Part 2: Test conditions for moulding and extrusion plastics". (Geneva, Switzerland).

Standardization, I.O.f. (2014). "ISO 13061-6:2014 Physical and mechanical properties of wood — Test methods for small clear wood specimens — Part 6: Determination of ultimate tensile stress parallel to grain". (Geneva, Switzerland).

Standardization, I.O.f. (2016). "ISO 1421:2016 Rubber- or plastics-coated fabrics — Determination of tensile strength and elongation at break". (Geneva, Switzerland).

Standardization, I.O.f. (2018). "ISO 527-3:2018 Plastics — Determination of tensile properties — Part 3: Test conditions for films and sheets". (Geneva, Switzerland).

Standardization, I.O.f. (2020). "ISO 3376:2020 Leather — Physical and mechanical tests — Determination of tensile strength and percentage elongation". (Geneva, Switzerland).

Standardization, I.O.f. (2021a). "ISO 527-4:2021 Plastics — Determination of tensile properties — Part 4: Test conditions for isotropic and orthotropic fiber-reinforced plastic composites". (Geneva, Switzerland).

Standardization, I.O.f. (2021b). "ISO 527-5:2021 Plastics — Determination of tensile properties — Part 5: Test conditions for unidirectional fiber-reinforced plastic composites". (Geneva, Switzerland).

Stecco, C., Pavan, P., Pachera, P., De Caro, R., and Natali, A. (2014). Investigation of the mechanical properties of the human crural fascia and their possible clinical implications. *Surgical and Radiologic Anatomy* 36(1)**,** 25-32. doi: 10.1007/s00276-013-1152-y.

Steiger, H.J., Aaslid, R., Keller, S., and Reulen, H.J. (1989). Strength, elasticity and viscoelastic properties of cerebral aneurysms. *Heart Vessels* 5(1)**,** 41-46. doi: 10.1007/bf02058357.

Stemper, B.D., Yoganandan, N., and Pintar, F.A. (2005). Methodology to study intimal failure mechanics in human internal carotid arteries. *J Biomech* 38(12)**,** 2491-2496. doi: 10.1016/j.jbiomech.2004.10.021.

Stouthandel, M., Vanhove, C., Devriendt, W., De Bock, S., Debbaut, C., Vangestel, C., et al. (2020). Biomechanical comparison of Thiel embalmed and fresh frozen nerve tissue. *Anatomical Science International* (95)**,** 399–407. doi: 10.1007/s12565-020-00535-1.

Stradins, P., Lacis, R., Ozolanta, I., Purina, B., Ose, V., Feldmane, L., et al. (2004). Comparison of biomechanical and structural properties between human aortic and pulmonary valve. *European Journal of Cardio-Thoracic Surgery* 26(3)**,** 634-639. doi: 10.1016/j.ejcts.2004.05.043.

Strauss, M.J., Miles, J.W., Kennedy, M.L., Dornan, G.J., Moatshe, G., Lind, M., et al. (2022). Full thickness quadriceps tendon grafts with bone had similar material properties to bone-patellar tendon-bone and a four-strand semitendinosus grafts: a biomechanical study. *Knee Surg Sports Traumatol Arthrosc* 30(5)**,** 1786-1794. doi: 10.1007/s00167-021-06738-x.

Sun, M.T., Pham, D.T., O'Connor, A.J., Wood, J., Casson, R., Selva, D., et al. (2015). The biomechanics of eyelid tarsus tissue. *J Biomech* 48(12)**,** 3455-3459. doi: 10.1016/j.jbiomech.2015.05.037.

Svensson, R.B., Hassenkam, T., Hansen, P., Kjaer, M., and Magnusson, S.P. (2011). Tensile force transmission in human patellar tendon fascicles is not mediated by glycosaminoglycans. *Connect Tissue Res* 52(5)**,** 415-421. doi: 10.3109/03008207.2010.551569.

Swank, K.R., Behn, A.W., and Dragoo, J.L. (2014). The Effect of Donor Age on Structural and Mechanical Properties of Allograft Tendons. *American Journal of Sports Medicine* 43**,** 453-459. doi: 10.1177/0363546514557246.

Tanaka, E., Shibaguchi, T., Tanaka, M., and Tanne, K. (2000). Viscoelastic properties of the human temporomandibular joint disc in patients with internal derangement. *J Oral Maxillofac Surg* 58(9)**,** 997-1002. doi: 10.1053/joms.2000.8743.

Tanios, F., Gee, M.W., Pelisek, J., Kehl, S., Biehler, J., Grabher-Meier, V., et al. (2015). Interaction of biomechanics with extracellular matrix components in abdominal aortic aneurysm wall. *Eur J Vasc Endovasc Surg* 50(2)**,** 167-174. doi: 10.1016/j.ejvs.2015.03.021.

Tantius, B., Rothschild, M.A., Valter, M., Michael, J., and Banaschak, S. (2014). Experimental studies on the tensile properties of human umbilical cords. *Forensic Science International* 236**,** 16-21. doi: 10.1016/j.forsciint.2013.12.002.

Tavares Monteiro, J.A., da Silva, E.S., Raghavan, M.L., Puech-Leão, P., de Lourdes Higuchi, M., and Otoch, J.P. (2014). Histologic, histochemical, and biomechanical properties of fragments isolated from the anterior wall of abdominal aortic aneurysms. *J Vasc Surg* 59(5)**,** 1393-1401.e1391-1392. doi: 10.1016/j.jvs.2013.04.064.

Temple, M.M., Bae, W.C., Chen, M.Q., Lotz, M., Amiel, D., Coutts, R.D., et al. (2007). Age- and site-associated biomechanical weakening of human articular cartilage of the femoral condyle. *Osteoarthritis Cartilage* 15(9)**,** 1042-1052. doi: 10.1016/j.joca.2007.03.005.

Temple-Wong, M.M., Bae, W.C., Chen, M.Q., Bugbee, W.D., Amiel, D., Coutts, R.D., et al. (2009). Biomechanical, structural, and biochemical indices of degenerative and osteoarthritic deterioration of adult human articular cartilage of the femoral condyle. *Osteoarthritis and Cartilage* 17(11)**,** 1469-1476. doi: 10.1016/j.joca.2009.04.017.

Teng, Z., Feng, J., Zhang, Y., Huang, Y., Sutcliffe, M.P., Brown, A.J., et al. (2015). Layer- and Direction-Specific Material Properties, Extreme Extensibility and Ultimate Material Strength of Human Abdominal Aorta and Aneurysm: A Uniaxial Extension Study. *Ann Biomed Eng* 43(11)**,** 2745-2759. doi: 10.1007/s10439-015-1323-6.

Teng, Z., Tang, D., Zheng, J., Woodard, P.K., and Hoffman, A.H. (2009). An experimental study on the ultimate strength of the adventitia and media of human atherosclerotic carotid arteries in circumferential and axial directions. *J Biomech* 42(15)**,** 2535-2539. doi: 10.1016/j.jbiomech.2009.07.009.

Teng, Z., Trabelsi, O., Ochoa, I., He, J., Gillard, J.H., and Doblare, M. (2012). Anisotropic material behaviours of soft tissues in human trachea: an experimental study. *J Biomech* 45(9)**,** 1717-1723. doi: 10.1016/j.jbiomech.2012.04.002.

Teng, Z.Z., Zhang, Y.X., Huang, Y., Feng, J.X., Yuan, J.M., Lu, Q.S., et al. (2014). Material properties of components in human carotid atherosclerotic plaques: A uniaxial extension study. *Acta Biomaterialia* 10(12)**,** 5055-5063. doi: 10.1016/j.actbio.2014.09.001.

Tenorio, L.E.M., Devine, K.J., Lee, J., Kowalewski, T.M., and Barocas, V.H. (2017). Biomechanics of human parietal pleura in uniaxial extension. *Journal of the Mechanical Behavior of Biomedical Materials* 75**,** 330-335. doi: 10.1016/j.jmbbm.2017.07.044.

Thomas, R.L., Kuiper, J.H., and Knight, T.P. (2020). Properties and Function of the Flexor Hallucis Capsularis Interphalangeus Tendon. *Clin Anat* 33(5)**,** 689-695. doi: 10.1002/ca.23490.

Ticker, J.B., Flatow, E.L., Pawluk, R.J., Soslowsky, L.J., Ratcliffe, A., Arnoczky, S.P., et al. (2006). The inferior glenohumeral ligament: A correlative investigation. *Journal of Shoulder and Elbow Surgery* 15(6)**,** 665-674. doi: 10.1016/j.jse.2005.11.006.

Tissakht, M., and Ahmed, A.M. (1995). Tensile stress-strain characteristics of the human meniscal material. *Journal of Biomechanics* 28(4)**,** 411-422. doi: 10.1016/0021-9290(94)00081-e.

Todros, S., Biz, C., Ruggieri, P., and Pavan, P.G. (2021). Experimental Analysis of Plantar Fascia Mechanical Properties in Subjects with Foot Pathologies. *Applied Sciences-Basel* 11(4)**,** 11. doi: 10.3390/app11041517.

Tokgoz, A., Wang, S., Sastry, P., Sun, C., Figg, N.L., Huang, Y., et al. (2022). Association of Collagen, Elastin, Glycosaminoglycans, and Macrophages With Tissue Ultimate Material Strength and Stretch in Human Thoracic Aortic Aneurysms: A Uniaxial Tension Study. *J Biomech Eng* 144(10). doi: 10.1115/1.4054060.

Tóth, M., Nádasy, G.L., Nyáry, I., Kerényi, T., Orosz, M., Molnárka, G., et al. (1998). Sterically inhomogenous viscoelastic behavior of human saccular cerebral aneurysms. *J Vasc Res* 35(5)**,** 345-355. doi: 10.1159/000025604.

Trabelsi, O., del Palomar, A.P., Lopez-villalobos, J.L., Ginel, A., and Doblare, M. (2010). Experimental characterization and constitutive modeling of the mechanical behavior of the human trachea. *Medical Engineering & Physics* 32(1)**,** 76-82. doi: 10.1016/j.medengphy.2009.10.010.

Trindade, V.L., Martins, P.A., Parente, M.P., Natal Jorge, R.M., Santos, A., Santos, L., et al. (2013). The influence of regional profiles and senescence on the biomechanical properties of the temporalis muscle. *J Biomech* 46(9)**,** 1592-1595. doi: 10.1016/j.jbiomech.2013.03.015.

Trindade, V.L.A., Martins, P.A.L.S., Santos, S., Parente, M.P.L., Jorge, R.M.N., Santos, A., et al. (2012). Experimental study of the influence of senescence in the biomechanical properties of the temporal tendon and deep temporal fascia based on uniaxial tension tests. *Journal of Biomechanics* 45(1)**,** 199-201. doi: 10.1016/j.jbiomech.2011.09.018.

Untaroiu, C.D., Lu, Y.C., Siripurapu, S.K., and Kemper, A.R. (2015). Modeling the biomechanical and injury response of human liver parenchyma under tensile loading. *J Mech Behav Biomed Mater* 41**,** 280-291. doi: 10.1016/j.jmbbm.2014.07.006.

van Alphen, G.W., and Graebel, W.P. (1991). Elasticity of tissues involved in accommodation. *Vision Res* 31(7-8)**,** 1417-1438. doi: 10.1016/0042-6989(91)90061-9.

van Noort, R., Martin, T.R., Black, M.M., Barker, A.T., and Montero, C.G. (1981). The mechanical properties of human dura mater and the effects of storage media. *Clin Phys Physiol Meas* 2(3)**,** 197-203. doi: 10.1088/0143-0815/2/3/003.

Vanags, I., Petersons, A., Ose, V., Ozolanta, I., Kasyanov, V., Laizans, J., et al. (2003). Biomechanical properties of oesophagus wall under loading. *Journal of Biomechanics* 36(9)**,** 1387-1390. doi: 10.1016/s0021-9290(03)00160-x.

Vande Geest, J.P., Dillavou, E.D., Di Martino, E.S., Oberdier, M., Bohra, A., Makaroun, M.S., et al. (2006). Gender-related differences in the tensile strength of abdominal aortic aneurysm. *Ann N Y Acad Sci* 1085**,** 400-402. doi: 10.1196/annals.1383.048.

Vogel, I., Petersen, A., Petersen, L.K., Helmig, R.B., Oxlund, H., and Uldbjerg, N. (2004). Biphasic effect of relaxin, inhibitable by a collagenase inhibitor, on the strength of human fetal membranes. *In Vivo* 18(5)**,** 581-584.

Vorp, D.A., Schiro, B.J., Ehrlich, M.P., Juvonen, T.S., Ergin, M.A., and Griffith, B.P. (2003). Effect of aneurysm on the tensile strength and biomechanical behavior of the ascending thoracic aorta. *Annals of Thoracic Surgery* 75(4)**,** 1210-1214. doi: 10.1016/s0003-4975(02)04711-2.

Voycheck, C.A., Luu, K., McMahon, P.J., and Debski, R.E. (2014). Collagen fiber alignment and maximum principal strain in the glenohumeral capsule predict location of failure during uniaxial extension. *Biomech Model Mechanobiol* 13(2)**,** 379-385. doi: 10.1007/s10237-013-0503-2.

Voycheck, C.A., Rainis, E.J., McMahon, P.J., Weiss, J.A., and Debski, R.E. (2010). Effects of region and sex on the mechanical properties of the glenohumeral capsule during uniaxial extension. *J Appl Physiol (1985)* 108(6)**,** 1711-1718. doi: 10.1152/japplphysiol.01175.2009.

Wagner, D.R., Reiser, K.M., and Lotz, J.C. (2006). Glycation increases human annulus fibrosus stiffness in both experimental measurements and theoretical predictions. *Journal of Biomechanics* 39(6)**,** 1021-1029. doi: 10.1016/j.jbiomech.2005.02.013.

Wagner, M., Walter, P., Salla, S., Johnen, S., Plange, N., Rutten, S., et al. (2018). Cryopreservation of amniotic membrane with and without glycerol additive. *Graefes Archive for Clinical and Experimental Ophthalmology* 256(6)**,** 1117-1126. doi: 10.1007/s00417-018-3973-1.

Walraevens, J., Willaert, B., De Win, G., Ranftl, A., De Schutter, J., and Vander Sloten, J. (2008). Correlation between compression, tensile and tearing tests on healthy and calcified aortic tissues. *Medical Engineering & Physics* 30 1098–1104. doi: 10.1016/j.medengphy.2008.01.006.

Wang, D.H., Makaroun, M., Webster, M.W., and Vorp, D.A. (2001). Mechanical properties and microstructure of intraluminal thrombus from abdominal aortic aneurysm. *J Biomech Eng* 123(6)**,** 536-539. doi: 10.1115/1.1411971.

Wang, R.Y., Arciero, R.A., Obopilwe, E., and Mazzocca, A.D. (2010). A comparison of structural and mechanical properties of tubularized and native semitendinosus graft. *Am J Sports Med* 38(6)**,** 1246-1249.

Weber, J., Agur, A., Fattah, A., Gordon, K., and Oliver, M. (2015). Tensile mechanical properties of human forearm tendons. *JOURNAL OF HAND SURGERY-EUROPEAN VOLUME* 40(7)**,** 711-719. doi: 10.1177/1753193415584715.

Weightman, B. (1976). Tensile fatigue of human articular cartilage. *J Biomech* 9(4)**,** 193-200. doi: 10.1016/0021-9290(76)90004-x.

Weightman, B., Chappell, D.J., and Jenkins, E.A. (1978). A second study of tensile fatigue properties of human articular cartilage. *Ann Rheum Dis* 37(1)**,** 58-63. doi: 10.1136/ard.37.1.58.

Weisbecker, H., Pierce, D.M., Regitnig, P., and Holzapfel, G.A. (2012). Layer-specific damage experiments and modeling of human thoracic and abdominal aortas with non-atherosclerotic intimal thickening. *J Mech Behav Biomed Mater* 12**,** 93-106. doi: 10.1016/j.jmbbm.2012.03.012.

Weisbecker, H., Viertler, C., Pierce, D.M., and Holzapfel, G.A. (2013). The role of elastin and collagen in the softening behavior of the human thoracic aortic media. *Journal of Biomechanics* 46(11)**,** 1859-1865. doi: 10.1016/j.jbiomech.2013.04.025.

Wertheimer, V., Grgic, I., Zelic, Z., Ivandic, Z., Koprivcic, I., Zelenic, M., et al. (2021). Biomechanical Analysis of the Gracilis and Superficial Third of the Quadriceps Tendons Concerning MPFL Biomechanics. *Tehnicki Vjesnik-Technical Gazette* 28(5)**,** 1575-1581. doi: 10.17559/tv-20210204092703.

Wolfinbarger, L., Zhang, Y.X., Adam, B.L.T., Homsi, D., Gates, K., and Sutherland, V. (1994). Biomechanical Aspects on Rehydrated Freeze-Dried Human Allograft Dura-Mater Tissues. *Journal of Applied Biomaterials* 5(3)**,** 265-270. doi: 10.1002/jab.770050313.

Wright, G.J., Coombs, M.C., Hepfer, R.G., Damon, B.J., Bacro, T.H., Lecholop, M.K., et al. (2016a). Tensile biomechanical properties of human temporomandibular joint disc: Effects of direction, region and sex. *Journal of Biomechanics* 49(16)**,** 3762-3769. doi: 10.1016/j.jbiomech.2016.09.033.

Wright, J.O., Skelley, N.W., Schur, R.P., Castile, R.M., Lake, S.P., and Brophy, R.H. (2016b). Microstructural and Mechanical Properties of the Posterior Cruciate Ligament A Comparison of the Anterolateral and Posteromedial Bundles. *Journal of Bone and Joint Surgery-American Volume* 98(19)**,** 1656-1664. doi: 10.2106/jbjs.16.00032.

Xiang, Y., Shen, M., Xue, C., Wu, D., and Wang, Y. (2018). Tensile biomechanical properties and constitutive parameters of human corneal stroma extracted by SMILE procedure. *J Mech Behav Biomed Mater* 85**,** 102-108. doi: 10.1016/j.jmbbm.2018.05.042.

Xiong, J., Wang, S.M., Zhou, W., and Wu, J.G. (2008). Measurement and analysis of ultimate mechanical properties, stress-strain curve fit, and elastic modulus formula of human abdominal aortic aneurysm and nonaneurysmal abdominal aorta. *Journal of Vascular Surgery* 48(1)**,** 189-195. doi: 10.1016/j.jvs.2007.12.053.

Xue, C., Xiang, Y.Q., Shen, M., Wu, D., and Wang, Y. (2018). Preliminary Investigation of the Mechanical Anisotropy of the Normal Human Corneal Stroma. *Journal of Ophthalmology* 2018**,** 7. doi: 10.1155/2018/5392041.

Xue, C., Xiang, Y.Q., Song, Y., Shen, M., Wu, D., and Wang, Y. (2021). Direct Evidence of Symmetry between Bilateral Human Corneas in Biomechanical Properties: A Comparison Study with Fresh Corneal Tissue. *Journal of Ophthalmology* 2021**,** 7. doi: 10.1155/2021/8891412.

Yamada, H., Sakata, N., Wada, H., Tashiro, T., and Tayama, E. (2015). Age-related distensibility and histology of the ascending aorta in elderly patients with acute aortic dissection. *J Biomech* 48(12)**,** 3267-3273. doi: 10.1016/j.jbiomech.2015.06.025.

Yan, S.H., Ou-Yang, H.K., Shan, Y.L., Luo, D.Z., Wang, H., and Zhang, K. (2016). Tensile biomechanical characteristics of human meniscus. *Emerging Materials Research* 5(1)**,** 44-49. doi: 10.1680/jemmr.15.00031.

Yoganandan, N., Pintar, F., Butler, J., Reinartz, J., Sances, A., Jr., and Larson, S.J. (1989). Dynamic response of human cervical spine ligaments. *Spine (Phila Pa 1976)* 14(10)**,** 1102-1110. doi: 10.1097/00007632-198910000-00013.

Zarzur, E. (1996). Mechanical properties of the human lumbar dura mater. *Arq Neuropsiquiatr* 54(3)**,** 455-460. doi: 10.1590/s0004-282x1996000300015.

Zellers, J.A., Eekhoff, J.D., Walk, R.E., Hastings, M.K., Tang, S.Y., and Lake, S.P. (2021). Human Achilles tendon mechanical behavior is more strongly related to collagen disorganization than advanced glycation end-products content. *Sci Rep* 11(1)**,** 24147. doi: 10.1038/s41598-021-03574-4.

Zimmern, P.E., Eberhart, R.C., and Bhatt, A. (2009). Methodology for Biomechanical Testing of Fresh Anterior Wall Vaginal Samples From Postmenopausal Women Undergoing Cystocele Repair. *Neurourology and Urodynamics* 28(4)**,** 325-329. doi: 10.1002/nau.20657.

Zwirner, J., Babian, C., Ondruschka, B., Schleifenbaum, S., Scholze, M., Waddell, N., et al. (2019a). Tensile properties of the human iliotibial tract depend on height and weight. *MEDICAL ENGINEERING & PHYSICS* 69**,** 85-91.

Zwirner, J., Koutp, A., Vidakovic, H., Ondruschka, B., Kieser, D.C., and Hammer, N. (2021a). Assessment of plantaris and peroneus tertius tendons as graft materials for ankle ligament reconstructions - A cadaveric biomechanical study. *Journal of the Mechanical Behavior of Biomedical Materials* 115**,** 8. doi: 10.1016/j.jmbbm.2020.104244.

Zwirner, J., Ondruschka, B., Pregartner, G., Berghold, A., Scholze, M., and Hammer, N. (2022). On the correlations of biomechanical properties of super-imposed temporal tissue layers and their age-, sex-, side- and post-mortem interval dependence. *Journal of Biomechanics* 130**,** 6. doi: 10.1016/j.jbiomech.2021.110847.

Zwirner, J., Ondruschka, B., Scholze, M., and Hammer, N. (2020a). Passive load-deformation properties of human temporal muscle. *Journal of Biomechanics* 106**,** 6. doi: 10.1016/j.jbiomech.2020.109829.

Zwirner, J., Ondruschka, B., Scholze, M., and Hammer, N. (2020b). Surface coating and speckling of the human iliotibial tract does not affect its load-deformation properties. *Scientific Reports* 10(1)**,** 20747. doi: 10.1038/s41598-020-77299-1.

Zwirner, J., Ondruschka, B., Scholze, M., Schulze-Tanzil, G., and Hammer, N. (2020c). Mechanical properties of native and acellular temporal muscle fascia for surgical reconstruction and computational modelling purposes. *J Mech Behav Biomed Mater* (108)**,** 103833. doi: 10.1016/j.jmbbm.2020.103833.

Zwirner, J., Ondruschka, B., Scholze, M., Schulze-Tanzil, G., and Hammer, N. (2021b). Biomechanical characterization of human temporal muscle fascia in uniaxial tensile tests for graft purposes in duraplasty. *Scientific Reports* 11(1)**,** 11. doi: 10.1038/s41598-020-80448-1.

Zwirner, J., Scholze, M., Ondruschka, B., and Hammer, N. (2020d). What is Considered a Variation of Biomechanical Parameters in Tensile Tests of Collagen-Rich Human Soft Tissues? - Critical Considerations Using the Human Cranial Dura Mater as a Representative Morpho-Mechanic Model. *Medicina (Kaunas)* 56(10). doi: 10.3390/medicina56100520.

Zwirner, J., Scholze, M., Waddell, J.N., Ondruschka, B., and Hammer, N. (2019b). Mechanical Properties of Human Dura Mater in Tension - An Analysis at an Age Range of 2 to 94 Years. *Sci Rep* 9(1)**,** 16655. doi: 10.1038/s41598-019-52836-9.
